# Comparison of C. elegans and C. briggsae Genome Sequences Reveals Extensive Conservation of Chromosome Organization and Synteny

**DOI:** 10.1371/journal.pbio.0050167

**Published:** 2007-07-03

**Authors:** LaDeana W Hillier, Raymond D Miller, Scott E Baird, Asif Chinwalla, Lucinda A Fulton, Daniel C Koboldt, Robert H Waterston

**Affiliations:** 1 Genome Sequencing Center, Washington University School of Medicine, Saint Louis, Missouri, United States of America; 2 Department of Genetics, Washington University School of Medicine, Saint Louis, Missouri, United States of America; 3 Department of Biological Sciences, Wright State University, Dayton, Ohio, United States of America; 4 Department of Genome Sciences, University of Washington, Seattle, Washington, United States of America; University of California Davis, United States of America

## Abstract

To determine whether the distinctive features of Caenorhabditis elegans chromosomal organization are shared with the C. briggsae genome, we constructed a single nucleotide polymorphism–based genetic map to order and orient the whole genome shotgun assembly along the six C. briggsae chromosomes. Although these species are of the same genus, their most recent common ancestor existed 80–110 million years ago, and thus they are more evolutionarily distant than, for example, human and mouse. We found that, like *C. elegans* chromosomes, *C. briggsae* chromosomes exhibit high levels of recombination on the arms along with higher repeat density, a higher fraction of intronic sequence, and a lower fraction of exonic sequence compared with chromosome centers. Despite extensive intrachromosomal rearrangements, 1:1 orthologs tend to remain in the same region of the chromosome, and colinear blocks of orthologs tend to be longer in chromosome centers compared with arms. More strikingly, the two species show an almost complete conservation of synteny, with 1:1 orthologs present on a single chromosome in one species also found on a single chromosome in the other. The conservation of both chromosomal organization and synteny between these two distantly related species suggests roles for chromosome organization in the fitness of an organism that are only poorly understood presently.

## Author Summary

The importance of chromosomal organization in the fitness of a species is only poorly understood. The publication of the C. elegans genome sequence in 1998 revealed features of higher level organization that suggested its chromosomes were organized into distinct domains. Chromosome arms were accumulating changes more rapidly than the centers of chromosomes. In this paper, we have compared the organization of the nematode C. briggsae genome with that of C. elegans. By building a genetic map based on DNA variations between two strains of C. briggsae, and by using that map to organize the draft genome sequence of C. briggsae published in 2003, we found the following: (1) Intrachromosomal rearrangements are frequent within and even between arms but are less common within central regions and between arms and centers. (2) Genes have remained overwhelmingly on the same chromosomes. (3) The distinctive features that distinguish C. elegans arms from centers also are seen in C. briggsae chromosomes. The conservation of these features between these two species, despite the approximately 100 million years since their most recent common ancestor, provides clear evidence of the selective advantages of the domain architecture of chromosomes. The continuing association of genes on the same chromosomes suggests that this may also be advantageous.

## Abstract 3

The conservation of both chromosomal organization and synteny between two distantly related species suggests roles for chromosome organization in the fitness of an organism.

## Introduction

The comparative analysis of the related nematodes Caenorhabditis elegans and C. briggsae offers a powerful approach toward understanding the genetic basis for the form and function of these simple animals. Studies to date have already yielded valuable insights into the evolution and role of particular sequences, genes, and pathways [1,2]. Morphologically, the two species are almost indistinguishable, despite the fact that their most recent common ancestor (MRCA) existed about 100 million years ago (Mya). Both are soil-dwelling, self-fertilizing hermaphrodites, with facultative males. Both have a ~100-megabase (Mb) genome apportioned into six chromosomes. Genes isolated in one species will frequently rescue mutants in the other [3,4]. Despite these similarities, nucleotide alignments (using the wobble-aware bulk aligner [WABA] algorithm [5]) of the complete genome sequence of C. elegans [6,7] with the draft sequence of C. briggsae strain AF16 reveals that 52.3% of the C. elegans genome and 50.1% of the C. briggsae genome aligns between the two species with the bulk of this in coding sequence [8]. The substantial body of knowledge accrued about C. elegans over the past few decades will help interpret the sequence similarities and differences. Much less is known about C. briggsae.

To facilitate the molecular genetic study of C. briggsae and thus enhance its utility for further comparative analysis, we sought to convert the whole genome sequence assembly into a genome map, in which the genome sequence and genetic maps are linked to each other through common markers across the chromosomes. Before our present work, the draft whole genome assembly contained 102 Mb of sequence in 142 physical map–based contigs (fpc contigs), with the remaining 6 Mb in 463 supercontigs (see Materials and Methods). The classical genetic map (Bhagwati Gupta, personal communication) has fewer than 40 mutants placed on the six linkage groups and only ten of these have a molecular assignment. The large number of contigs and the paucity of genetic mapping data did not allow meaningful merging of the two maps.

We undertook the construction of a genome map by first generating a genetic map using molecularly based single nucleotide polymorphism (SNP) markers. This more detailed genetic map based on SNPs would be of use in its own right, for example, simplifying positional cloning of genetically defined genes. But it would also provide long-range continuity, which would in turn allow the placement of much of the assembled sequence along the chromosomes. This long-range map of the genome would in turn allow a direct comparison of chromosomal organization in C. briggsae to the distinctive features of C. elegans organization [6,9]. Using other wild isolates of *C. briggsae,* we discovered thousands of SNPs. By genotyping selected SNPs across recombinant inbred (RI) lines between the sequenced strain (AF16) and the SNP source strains, we generated a genetic map. We then combined the resultant genetic map and the sequence assembly information to place 91.2 Mb of sequence onto the six linkage groups, with another 9.9 Mb tentatively associated (but not ordered) with chromosomes.

The integrated map allowed us to correct several misassemblies in the initial C. briggsae sequence. Of broader interest, we were also able to explore chromosomal scale phenomena. Like in *C. elegans,* rates of recombination appear much higher on arms than in central regions for the autosomes. Autosome arms and centers also differ in their repeat content, coding density, and fraction of highly conserved genes, as is seen in C. elegans. Unexpectedly, the comparison also revealed an extensive conservation of synteny between the two organisms, with the vast majority of genes with 1:1 orthologs that reside on one chromosome in one species lying on a single chromosome in the other. Long-range gene order within the chromosomes has been substantially altered in the 100 million years (Myr) since their MRCA, but despite these rearrangements, sequences tend to remain in their respective domains of arm or center. Our findings support the emerging recognition of the importance of overall chromosomal organization in metazoans.

## Results

### SNP Discovery

To find a suitable strain for SNP discovery, we investigated four independent wild isolates that grow well in culture, are interfertile with the sequenced AF16 strain, and represent both tropical and temperate groups [10,11] (Table 1). We initially aligned a small number of random genomic sequences against the AF16 assembled sequence to ascertain the approximate incidence of single nucleotide variation. Two temperate strains of Japanese origin (HK104 and HK105) both had relatively high rates of difference (~1 SNP/110 bases) while the Hawaiian (VT847) and the Ohio (PB800) strains (tropical and temperate respectively) had apparently lower rates (see Materials and Methods for details of SNP detection). We selected one strain of each level (HK104 and VT847) for more extensive SNP discovery. From 8,405 and 9,970 aligned sequence reads from whole genome shotgun libraries from each strain we identified respectively 32,246 and 14,183 substitutions with Phred [12] quality scores of greater than 35, giving overall rates of 8.7 and 3.2 per kilobase. We also identified a number of candidate small insertion/deletion differences (7,118 events affecting 18,196 bases and 3,575 events affecting 8,315 bases, respectively).

**Table 1:**
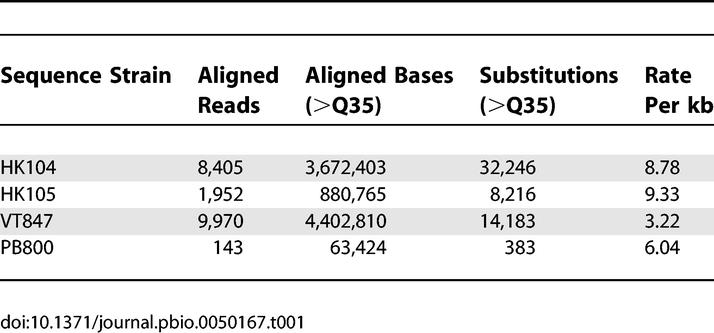
Variations Detected in Four C. briggsae Strains

### Construction of the Genetic Map

To construct a genetic map, 390 SNPs distributed across the sequence were tested against 93 AF16 × HK104 RI lines and the parental strains [10] using the fluorescent polarization detection (FP-TDI) assay (Vieux et al. 2002; see Materials and Methods for details of SNP assay). To maximize the amount of sequence mapped and to provide an independent check of the assembly, the 390 SNPs were selected from the larger supercontigs, thus ensuring that the larger physical map–based contigs (called fpc contigs for simplicity, after the program used to assemble the physical map [13]) would contain multiple markers and thus serve to check the assembly. In about a quarter of the cases, a second SNP was selected within a single supercontig to test the assembly at this level. A SNP was declared as mapped when the assays were successful on between 80% and 100% of the 95 strains tested, with a total of 248 SNPs (64%) meeting this criterion. Some 84 SNPs (22%) had success rates between 0% and 40% and were deemed failures. The high rate of failures was likely caused by PCR problems due to unaccounted SNPs in primer sites, a problem faced by all investigated genotyping platforms [14]. Some five SNPs (1.3%) were monomorphic and likely due to false SNP calls. Other SNPs failed quality control tests or had success rates of 40–80%.

These same SNP assays were also tested against the VT847 strain, for which RI lines were also available. Relatively few of the AF16/HK104 SNPs were polymorphic between AF16 and VT847, suggesting that the overlap in variation between the HK104 and VT847 is very limited. This meant that genotyping of these additional RI lines with these markers added little new map information.

We tested several different parameters for map construction, using the program Map Manager QTXb20 (http://www.mapmanager.org/ [15]). The different versions varied in map length per chromosome, total map length, and in the local order of markers within a chromosome, but assignment of markers to common linkage groups was a robust feature of the maps. The latter was due in part to the large number of nonrecombinant chromosomes in the RI lines (35–60% per chromosome), which allowed ready assignment to linkage groups. Based on these experiences, we adopted the following strategy to build version 3.0 of the genetic map: we used an initial set of 115 very high quality markers (>95% call rates) and a second set of slightly lower quality (80–95% call rates). We used the Haldane function and an initial probability of incorporation of a SNP into the map of 10^−6^. Seven linkage groups were formed, one with only two SNPs (cb23233 and cb23314). We reduced the probability required for incorporation to 10^−3^, and the latter group was incorporated into the end of chromosome CbIV. Thus the number of linkage groups matched the observed number of chromosomes (Table 2). The program provided map positions in centimorgans (cM) for each of the incorporated markers, with each of the chromosomes approximately 50 cM in length.

**Table 2:**
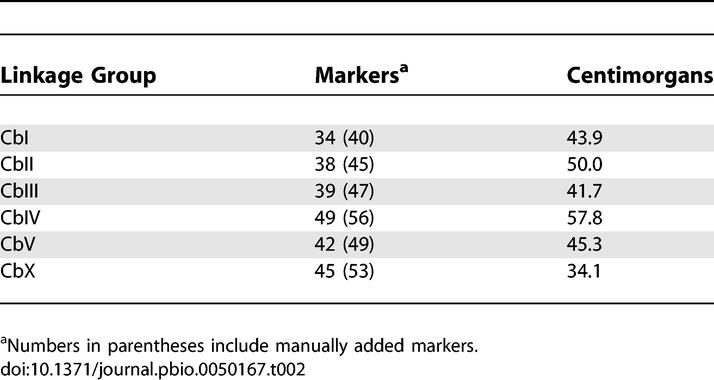
The 247 Markers Fall into Six Linkage Groups

Inspection of the raw data in the version 3.0 map in conjunction with the marker order in the sequence assembly highlighted places where markers of equivalent or nearly equivalent position in the genetic map could be shuffled to reconcile their order with that in the assembly. In addition the initial genetic map of the X chromosome (CbX; see below for chromosome assignments) showed a number of inconsistencies with the sequence assembly that could all be reconciled by a single inversion of the central segment of the genetic map for CbX. Additional recombinant data obtained for CbX from an experimental cross (see Materials and Methods) supported the revised genetic marker order. These changes were incorporated into version 3.1. Finally, inspection of the raw data in conjunction with the known groupings of markers based on the assembly suggested alternate orders of markers on chromosomes CbI, CbIV, and CbV that reduced the number of multiply recombinant chromosomes. These changes reduced overall map length by over 16 cM and did not reduce logarithm of the odds scores (logarithm of the odds score is a statistical estimate of whether two loci are likely to be near each other on a chromosome and therefore likely to be inherited together) of any markers below the threshold; they were incorporated into the genetic map to produce version 3.2.

Using this framework map, inspection of the remaining markers with lower call rates indicated that 44 of them could be readily linked to chromosomes and tentatively positioned within the chromosome. These added markers sometimes helped in orienting contigs and in five cases, positioned previously unplaced contigs. However, the lower overall call rates of these markers make their placement less certain.

### Segregation of Parental Markers

**Figure 1:**
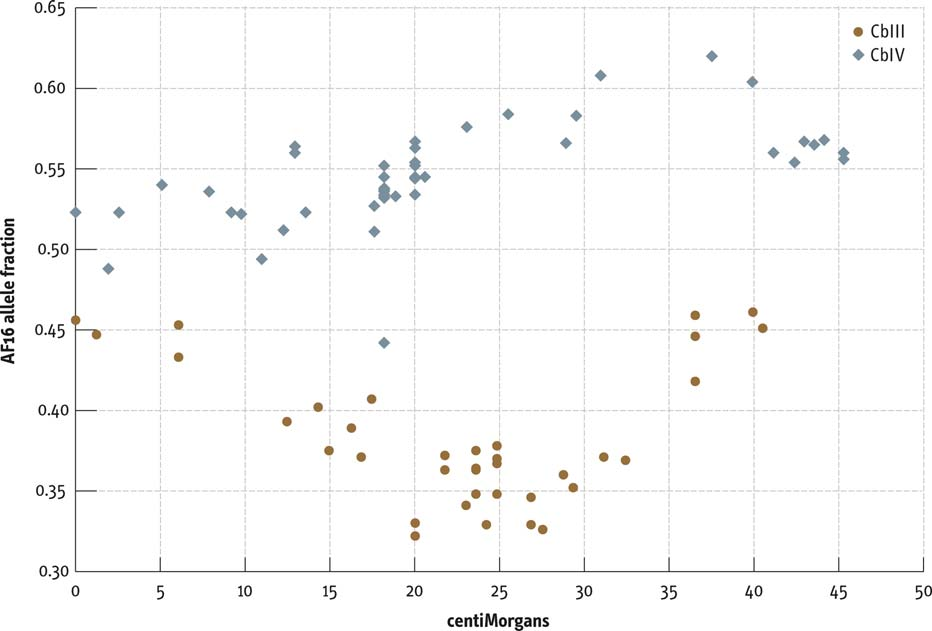
Percent Recovery of the AF16 Allele in the RI Lines for Each of the Markers Plotted against Its Position in cM on C. briggsae Chromosomes CbIII and CbIV Alleles from AF16 are less frequently recovered for much of CbIII, whereas for CbIV, the opposite holds. See Dataset S2 for other chromosomes.

With the genetic map in place, we examined the frequency of parental alleles within the RI lines across the chromosomes. For chromosomes CbII and CbX, there was little variation from the expected value of 50% for each marker. But for other chromosomes, there were regions of biased representation of the AF16 and HK104 alleles. For example, the AF16 allele was consistently underrepresented for most of CbIII, whereas it was overrepresented for much of chromosome CbIV (Figure 1). Chromosomes CbI and CbV also showed biased representation, but over more limited regions (see Datasets S1 and S2). The biased representation of alleles presumably reflects some selective advantage for offspring with these regions, either singularly or in combination. The selection of the first progeny at each generation in establishing the RI lines may have contributed to this bias. The relatively small number of recombinant events in these lines however precludes finer localization of such factors.

### Integrating Genetic and Sequence Maps

The sequence-based markers used in the construction of the genetic map allowed for ready integration of the genetic and sequence maps into a genome map. The association of a genetic marker with a supercontig and, in turn, an fpc contig positioned that sequence on a specific chromosome, and when multiple, genetically separated markers were assigned to a single sequence assembly, that sequence could be oriented. Generally, multiple markers from the same supercontig or fpc contig had adjacent positions in the genetic map, confirming the assembly in these instances.

However, markers assigned to 21 sequence assemblies were derived from more than one linkage group, indicating an error in either the genetic linkage assignment or in the sequence assembly. Because marker assignment to linkage groups was a robust feature of the genetic map and inspection of the raw data revealed no problems with the assignment in these discordant cases, the sequence map was probed for possible errors. Only one discrepancy was noted among 68 supercontigs with more than one marker, suggesting that misassemblies within supercontigs (constructed by using read-pair information to link sequence contigs) were unlikely to account for the bulk of the observed discrepancies. On the other hand, we noted that most markers with discordant linkage fell on fpc contigs (in which supercontigs were linked based on the physical clone map information). Detailed inspection showed that in these cases, a join based on the physical clone map information fell between discordant markers.

**Figure 2:**
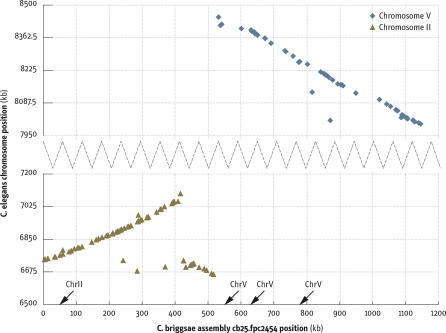
Example of the Transition in Synteny for an Assembly with Discrepant Genetic Markers For fpc contig cb25.fpc2454, the positions of 1:1 orthologs pairs are plotted both along the C. briggsae sequence (*x*-axis) and the C. elegans genome sequence (*y*-axis). The regions shown from two C. elegans chromosomes accounted for almost all of the orthologs from cb25.fpc2454. The positions of the SNP markers with their C. briggsae genetic map assignments are indicated along the *x*-axis. The region between the rightmost chromosome II ortholog at 524,614 and the leftmost chromosome V ortholog at 531,820 contained a single gap at 527,846 between supercontigs; the fpc contig was split at this gap.

Once the conservation of synteny between C. elegans and C. briggsae chromosomes was established (see below), the 1:1 orthology landmarks were used to delimit the region with the assembly problem, making it clear that the discrepancies arose because of false joins based on the lower resolution physical clone map (Figure 2). Inspection of the physical map in a sample of these regions revealed questionable clone overlaps often accompanied by an editor's comment to that effect, consistent with a misassembly at that point. As a result, 27 breaks were made in the fpc contigs at the site defined by the orthology landmarks (renamed as segments a, b, etc. of the parent contig). The single discordant supercontig was also broken at a site bounded by the ortholog landmarks. These breaks in the sequence assembly eliminated the inconsistencies between assignment of the markers to sequence assemblies and linkage groups (Table 3).

**Table 3:**
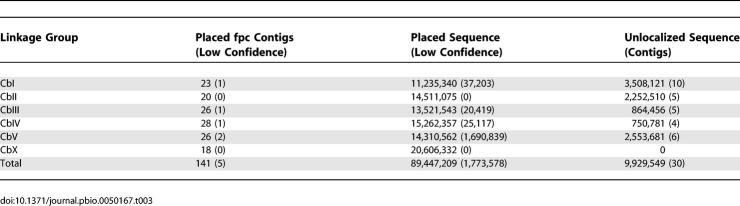
Integration with Sequence Map

Obviously, other misassemblies may remain undetected, because misassembled regions failed to have a genetic marker. Investigation of the entire sequence for clusters of discordant orthologs suggests five regions of more than 50 kilobases (kb) that are likely candidates for misassembly. Further, our analysis is less sensitive to misassemblies within the same chromosome, because precise order within linkage groups is less robust, making detection harder. Nonetheless, with one exception, markers in a single sequence assembly lie adjacent to one another in the current map. In the exception (cb25.fpc4010), a high-quality marker maps to the right end of chromosome CbIII, whereas two low-confidence markers suggest positions near the opposite end. Further, with one exception, multiple markers in a single sequence assembly fall in an order consistent with the genetic map order. In the single exception, a simple inversion of a pair of SNP markers in cb25.fpc3752 would reconcile the maps. However, we only altered the sequence assembly where there were compelling genetic data that the assembly was in error.

The integrated genetic and sequence map provide an initial genome map. The confidently placed genetic markers position 141 sequence assemblies, accounting for 89.4 Mb of the sequence along the chromosomes, with 42 of these oriented, accounting for 47.7 Mb. Inclusion of the lower-confidence markers provides tentative positions for an additional five assemblies, containing 1.8 Mb. By using read-pair information for assemblies adjacent in the genetic map, we were able to orient an additional 45 contigs, bringing the total oriented sequence to 67.3 Mb. In addition, by considering local order of 1:1 orthologs in both species (see below), we could tentatively order an additional 4.4 Mb. This reconciled genome map is reflected in version 3.3 of the genetic map.

### Recombination Rates Vary along the Chromosomes

**Figure 3:**
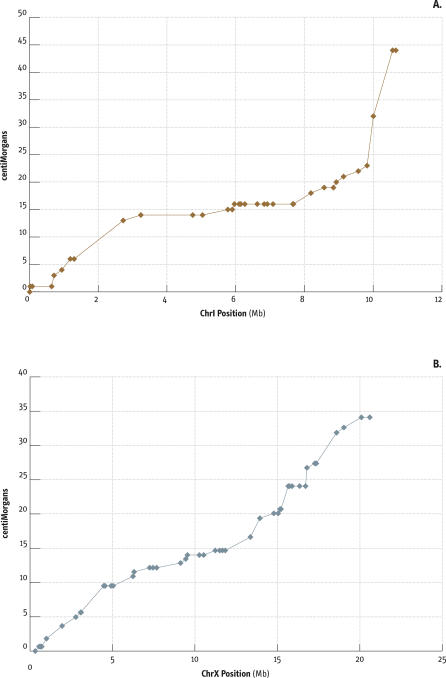
Recombination and Physical Distance in C. briggsae The panels show Marey plots [16] for chromosomes CbI (A) and CbX (B) in which the position in cM is plotted against the sequence position for each SNP marker. Recombination is high on the arms of CbI and low in the center. Other autosomes broadly follow this pattern (Figure S4). The difference between arms and centers on CbX is less marked.

In C. elegans, a distinctive feature of the genetic map is the reduced recombination per Mb of the centers of the autosomes compared with the arms [16]. We looked at the recombination rates across the C. briggsae autosomes and the putative X chromosome (see below) to see if the same features existed. Similar to that of *C. elegans,* each of the C. briggsae autosomes shows reduced recombination in the centers compared to the arms (Figure 3A, Datasets S3 and S4, and Figures S1–S4). Indeed, no recombinant events were observed in the RI lines over several megabases of the centers of several chromosomes, even though 60–70 recombinant events were observed on the 11–16-Mb autosomes. In contrast, recombination rates are more uniform on the presumptive X chromosome (Figure 3B).

These observations must be interpreted with some caution, because the C. briggsae genome map contains only 85% of the sequence, and assembly biases could mean that much of the unassigned sequence belongs on the arms. Further, some biases were introduced in the recovery of the RI lines, as noted above, which might also distort recombination rates. Finally the sequence differences and perhaps even inversions between the two strains could reduce recombination rates in local regions. Nonetheless, the general features seen here seem likely to be reflected in a more comprehensive map.

### Repeats, Genes, and Conserved Gene Distribution

**Figure 4:**
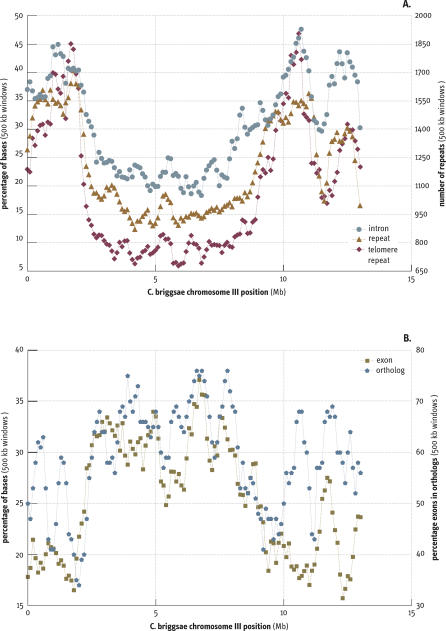
Variation of Features by Chromosomal Region Illustrated Using C. briggsae Chromosome III Features were examined in 500-kb windows in 100-kb steps along each chromosome. (A) The greater percentage of intronic sequence, the increased density of percentage of repeats (scale on left axis), and the greater number of telomere repeat sequences (TTAGGC) on the arms of CbIII (scale on the right axis). (B) The percentage of sequence present in exons (scale on left axis) and the percentage of exons in 1:1 orthologs (scale on right axis) for CbIII. Other autosomes all show this general pattern, with some variation for each feature (see Figures S1 and S2 for both C. briggsae and C. elegans).

In addition to the marked variation in recombination rates along the autosomes in *C. elegans,* repeat density and gene density were found to vary by region [6] . We observed similar variation in density of these features in the C. briggsae autosomes, with the repeat density higher and intron length greater on the arms and exon density greater in the centers (Figure 4, Datasets S3 and S4, and Figures S1 and S2). Again, as seen in C. elegans, telomere related repeats (TTAGGC) show a particularly marked difference in distribution. Strikingly, 1:1 orthologs, even after accounting for the greater exon density in the centers, are more common in the centers.

### Conservation of Synteny between C. elegans and C. briggsae Genomes

With the bulk of the C. briggsae genome placed along chromosomes, the conservation of synteny (using synteny here in the originally defined sense of genes on the same linkage group or chromosome) and colinearity (meaning the order of genes along the chromosome) between C. elegans and C. briggsae could be investigated directly across the whole genome. Early analyses of colinearity, using clone-based datasets of limited sequence continuity, estimated median tract lengths of <10 kb in one study [5] and 17 kb for autosomes and 41 kb for the sex chromosome in a second study [17]. The initial analysis of the C. briggsae whole-genome assembly observed a mean of 37,472 base pairs (bp) and a median 5,557 bp with a maximum block of 1.68 Mb [8]. This initial analysis used genome-wide alignment data and allowed regions to match as many as five segments in the reciprocal genome. Inspection of the junctions between the 4,837 candidate colinear blocks (minimum length 1.8 kb) suggested the breakpoints represented 1,384 inversions, 244 translocations, and 2,735 transpositions.

To make the present analysis less sensitive to repeated sequences and to small blocks of similarity that may have arisen by the large number of transposition events, we began by identifying 9,767 1:1 gene pairs (where each gene was represented only once in its genome and matched only one gene in the other genome) using the previously defined gene set [8]. These data provide an unambiguous orthologous landmark on average about every 10 kb. For those sequence assemblies that had only one genetic marker or that had genetic markers all on a single linkage group in the initial map, we found that the 1:1 orthologs on that assembly overwhelmingly derived from a single C. elegans chromosome. The same observations held for the corrected assemblies. More remarkably, we noted for sequence assemblies assigned unambiguously to the same C. briggsae linkage group that the 1:1 orthologs assignments were consistently from a single C. elegans chromosome (Table 4). Exceptional orthologs were often isolated, singular events. This remarkable conservation of synteny between the two species allowed us to assign not just regions but each of the entire C. briggsae linkage groups to its corresponding C. elegans chromosome.

**Table 4:**
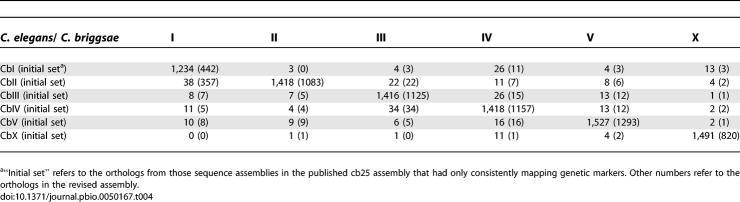
Reciprocal Mapping of 1:1 Orthologs

**Figure 5:**
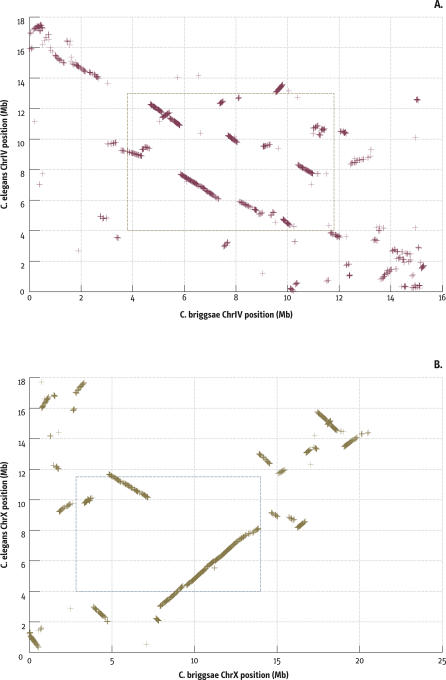
Chromosomal Positions of Orthologs The chromosomal positions of the orthologs in the two species reveals colinearity and breakpoints. The positions of ortholog pairs along the chromosomes of the two species are plotted against one another for chromosomes IV (A) and X (B). The position of each pair is designated with a “+”. Extensive colinearity of pairs produces an apparent line. The box in the center of each panel (dotted line) delimits the region of low recombination based on inspection of the Marey plots (Table 6). See Figure S4 for other chromosomes and for similar data for C. elegans.

To look at the colinearity of the orthologs within chromosomes, we plotted their location in each of the pairs of syntenic chromosomes (Figure 5, Dataset S5, and Figure S3). There have been extensive intrachromosomal rearrangements, but large colinear blocks remain, especially in the centers. More interestingly, sequences that are in the central, low-recombination segment of one species tend to be in the corresponding region in the other species. By contrast, there is mixing between the two arms.

To quantify this, we established blocks of sequence with the same order of genes in the two genomes allowing minor exceptions (see Materials and Methods). Our methods yielded only 851 blocks using a minimum block size of one ortholog, with only a third of these more than 50 kb long. Because our analysis excludes repeated sequences, these numbers do not reflect most transposition events, which formed the bulk of the rearrangements detected in [8]. Nonetheless, 351 of the 1:1 ortholog blocks are small enough (<20 kb) to be consistent with transposition events. Only 12 blocks greater than 20 kb involve nonsyntenic orthologs and might represent translocations; none of these have confirmatory genetic markers and could all represent assembly problems. Thus the only confirmed rearrangements represent intrachromosomal events. Their distribution across the chromosomes is distinctly nonrandom. As seen in Table 5, the block size of the X chromosomes is considerably larger than for the autosomes, and similarly within the autosomes, the block size in the centers is much larger than the arms. The median for the autosomes is similar to that obtained in [17], whereas the median for the X is considerably larger, perhaps because of the greater continuity of the sequence in our study.

**Table 5:**
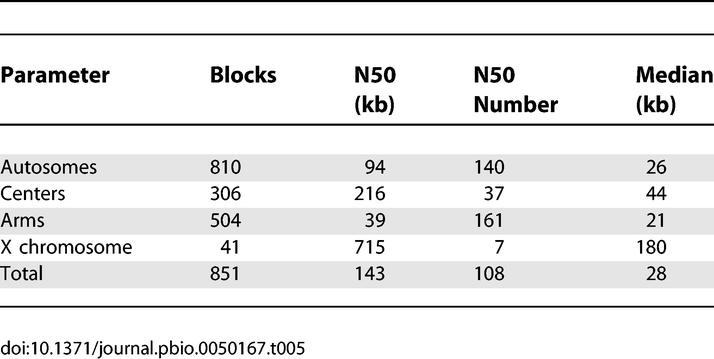
Colinear Block Size Characteristics

**Table 6:**
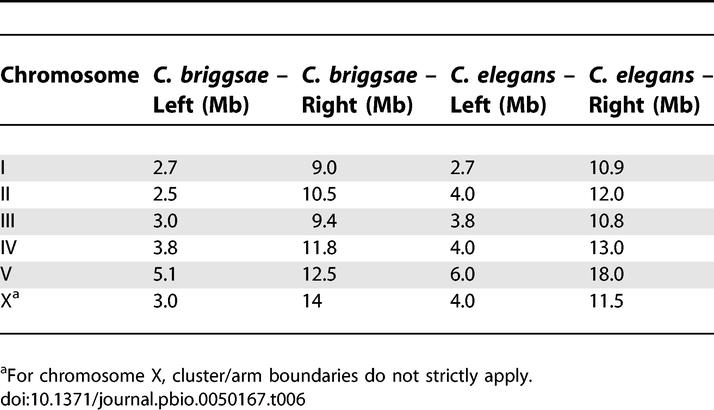
C. briggsae/C. elegans Cluster/Arm Boundaries

### Syntenic and Nonsyntenic Orthologs

**Figure 6:**
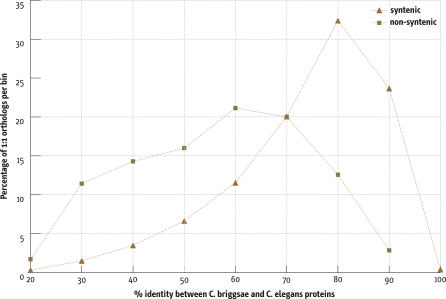
Sequence Similarity of Syntenic and Nonsyntenic Ortholog Pairs Syntenic and nonsyntenic ortholog pairs differ in their sequence similarity. The distribution of the percent identity, binned in five percentile bins, is shown both for pairs on the same chromosome (syntenic) and on different chromosomes (nonsyntenic).

Given the overwhelming tendency of orthologs to remain on the same chromosome, we investigated the nonsyntenic ortholog pairs to see what features might distinguish them from syntenic pairs. To minimize the likely contamination of the nonsyntenic set with misassemblies, we excluded 12 larger clusters of nonsyntenic orthologs (see Materials and Methods). The most distinctive difference between the two sets was the lower percent identity of the aligned nonsyntenic pairs (Figure 6). These differences existed among pairs regardless of whether the members of the pair lay both on chromosome arms, both in chromosome centers, or one on an arm and one in the center.

One explanation for the greater divergence of the nonsyntenic ortholog pairs might be that the true ortholog is missing in the draft C. briggsae sequence. We looked for evidence of this by finding the 1:1 orthologs (e.g., A and C) flanking the C. elegans member of a nonsyntenic ortholog pair, (ABC, where B is from the nonsyntenic pair) and then searching the region between the C. briggsae orthologs of A and C for evidence of large gaps or partial genes. Of 175 nonsyntenic ortholog pairs, we detected homology in the interval defined by the flanking orthologs for only 19 cases, and only 29 regions had 4% or more of the interval as uncalled bases (Ns). Almost half the intervals had less than 1% of the sequence as Ns. Thus, while the draft nature of the C. briggsae sequence may result in incorrect assignment of 1:1 orthology, producing an apparent increased divergence, it seems unlikely to account for the bulk of our observations.

## Discussion

### SNPs

The comparison of random clone sequences from whole genome shotgun libraries from the Japanese (HK104) and Hawaiian (VT847) isolates with the genome assembly of AF16 provided in each case adequate numbers of widely distributed SNPs to develop markers across the genome assembly. The sequence generated also provides the opportunity for more in-depth studies of patterns of variation among the different isolates. In this study we have confined our analysis to the overall rates of differences, determined by the simple method of scoring base differences between aligned sequences with quality scores >35. With this quality score cutoff, errors should contribute a false SNP no more than one per 3,200 bases, and given that most bases have quality scores well above this, the contribution is likely to be much smaller. Since the observed rates of difference considerably exceed this, errors will only slightly inflate the observed rates. Indeed, of the more than 320 SNP assays that provided data, only five (1.5%) were monomorphic.

The SNP rates we observed between these C. briggsae strains are higher than those observed between the most divergent C. elegans strains tested to date, with the HK104/AF16 differences about 8-fold higher and the VT847/AF16 differences about 3-fold higher than rates observed in similar experiments between N2, the standard strain of C. elegans, and CB4856, a strain from Hawaii that is among the most divergent strains yet isolated [18,19]. The SNP rates we observed for both VT847 and HK104 compared with AF16 are similar to those reported by studies focused on a few genes [20,21]. We also looked at regions of overlap of VT847 and HK104 sequences (total 129 kb) and noted that few differences were shared between the strains. Similarly we observed that the HK104/AF16 SNP assays were predominantly monomorphic when assayed against VT847/AF16 RI lines. These results are consistent with those of [21], on studies of 4.2 kb of sequence from six genes. The authors of [21] noted that strains from temperate regions across the globe, including HK104, HK105, and PB800, had little diversity among themselves, but were more variant as groups from tropical strains, which include both AF16 (India) and VT847. In contrast to the temperate strains, the tropical strains contained considerable diversity. These results suggest that the effective population size of C. briggsae may be several-fold larger than that observed for C. elegans.

Initial analysis suggests that the overall SNP rates may be greater on chromosome arms than in the centers. However, the differences in gene density and other features between the chromosomal regions may contribute to the apparent rate differences. A more careful parsing of the sequence reads among the features of the genome, a process now underway (LW Hillier and RH Waterston, unpublished data), will be required to evaluate the different regions.

### The RI Lines and Genetic Map

The placement of 248 markers onto six linkage groups is in accord with cytogenetic estimates of chromosome number [22]. The observed length in centimorgans of the autosomes is consistent with the hypothesis that each chromosome undergoes one recombinant event per meiosis, as is thought to be the case for C. elegans. However for CbX, the total length was only 34 cM. Of course the present markers may not extend to the ends of the chromosome, although the X, at more than 20 Mb, is the largest of the chromosomes and had no additional assemblies assigned to it based on ortholog assignments. Also, the two strains used to generate the RI lines might differ significantly in some regions in the genome, reducing recombination, e.g., through an inversion. If the X length is not artifactually short for one of the reasons given above, the genetic length of 34 cM would suggest that other mechanisms exist to ensure normal segregation of the X chromosome. Such mechanisms must exist in males, which are XO, and might be operative in XX animals in C. briggsae.

Although the RI lines served adequately to generate the map, they had shortcomings that might be improved in future studies. There was clearly biased recovery of some markers, with markers from the AF16 strain underrepresented on chromosome CbII and overrepresented on CbIV. This bias might be readily corrected by a more-random selection of progeny to establish each line. In addition, the RI lines had relatively few recombinant events. As a result, central regions of low recombination often contain several successive markers at the same distance. Strategies to establish lines that allowed several rounds of interbreeding would capture more events.

### The Integrated Genomic Map

The long-range continuity of the genetic map served to order and in many cases orient more than 90 Mb of the sequence assemblies along the chromosomes. Combining this with linking information from read-pairs and ortholog local colinearity, additional order and orientation of the contigs was provisionally imposed on the map. By exploiting the conservation of synteny, another 9 Mb could be tentatively assigned to chromosomes, although not ordered along them.

The conflicts between the genetic map and sequence assembly exposed misassemblies in the whole genome assembly. By carefully defining a set of 1:1 orthologous genes between the two species, the extensive conservation of synteny between the two species became more apparent and made clear that the problems lay in the assembly. The analysis also suggests at least another five regions of potential misassembly, each spanning more than 79 kb with a cluster of ten or more orthologs matching to a nonsyntenic chromosome. Smaller clusters of genes from nonsyntenic chromosomes also exist, but the fraction of these (or indeed the larger clusters) that represent assembly errors is uncertain. Positioning markers within these regions and testing them against the RI lines should distinguish misassembly from rearrangements.

The integrated map revealed that organization into arms and centers for a number of features found in C. elegans is also present in C. briggsae. These include the rates of recombination as a function of physical distance (Marey maps), the distribution of repeats and exons and the size of introns. Comparative analysis also shows a relative paucity of 1:1 orthologs in the arms as opposed to the centers, beyond that expected from the difference in exon density alone.

The maintenance of this distinctive organization over approximately 200 My of evolution, and despite numerous intrachromosomal inversion events, strongly supports the selective advantage this organization confers. The enrichment for strongly conserved genes with yeast and for 1:1 orthologs in the centers suggests that genes are protected in this environment from the mutagenic effects of the high recombination and associated transposable element (TE) activity that is prevalent on the arms. By contrast, the arms are enriched for rapidly evolving gene families, where recombination, higher mutation rates, and TEs may facilitate family expansion and rapid gene adaptation [23]. The association between regions of higher recombination and more rapidly evolving genes has been reported in other species as well, including yeast [24] and Drosophila [25,26].

### The Conservation of Synteny

The genome map revealed a striking degree of synteny conservation. More than 95% of 1:1 orthologs remain on the same autosome despite the extensive evolutionary time since the MRCA. For the X chromosome, the conservation is even greater, with about 97% of orthologs remaining syntenic in accord with theory [27]. Even this may underestimate the extent of conservation, since misassemblies may still contribute to some of the nonsyntenic regions.

The conservation of synteny in worms does not reflect a lack of overall rearrangements, however, since hundreds of rearrangements have occurred intrachromosomally. But within chromosomes, the observed breakpoints are not randomly distributed, with the block size much greater in the centers. Nor is there substantial mixing of the centers with the arms.

The extensive conservation of synteny between C. elegans and C. briggsae may extend beyond the genus to more distantly related nematodes. Analyses of short stretches of Pristionchus pacifica and Brugia malayi with C. elegans genomic sequence suggest that, although local gene order may be altered over this evolutionary distance, orthologs remained overwhelmingly on a single chromosome [28,29].

Our results are in striking contrast to the observations in mammals. Mouse-human comparisons show extensive mixing of DNA between chromosomes [30] with the notable exception of the X chromosome. Using a simple two-hit model to account for the difference in chromosome number, and the ratio of break points attributable to translocations and intrachromosomal rearrangements in mammals to estimate the expected number of translocations in nematodes, the failure to observe any validated translocation events in nematodes is highly significant (*p* < 0.0001). Even within primates synteny is often not conserved. For example, human chromosome 2 represents a fusion of 2 smaller chromosomes present in the MRCA with chimpanzee [31], and the gibbon branch has experienced an exceptional number of inversions and translocations [32,33].

Our findings are more similar to observations in the various species of the Drosophila genus. Muller recognized as early as 1940 that the chromosome arms of D. melanogaster are largely maintained as intact, though internally rearranged, units in other species of the genus. Recent analysis of genome sequences reveal just two pericentric inversions among the dozen species with a total branch length of more than 200 My [34]. The vast majority of genes remain on the same Mullerian element, as the arms have come to be called, although elements may fuse or split at centromeres and there are extensive rearrangements within elements.

What accounts for the marked difference between the Caenorhabditis and Drosophila species and vertebrates? We presume that interchromosomal translocations occur but rarely become fixed in these invertebrates. In contrast, such events appear to be fixed more frequently in vertebrates.

The paucity of translocations in worms and flies might be explained, at least in part, by larger effective population sizes in invertebrates. Before fixation, the half translocations will suppress recombination and their segregation will produce aneuploid genotypes that would be selected against in both vertebrates and invertebrates. Larger effective population sizes (*N*_e_) would lead to stronger selection against such unfavorable traits. Estimates of *N*_e_ for humans and for the common ancestor of humans and chimpanzees are 10,000 and 52,000–96,000, respectively [35,36]. Estimates of *N*_e_ for C. elegans and C. briggsae are similar, 9,600 and 60,000, respectively [21,37]. However, C. elegans and C. briggsae are hermaphroditic whereas their most recent common ancestor likely was dieocious [38]. C. remanei, the dieocious sister species of C. briggsae and perhaps more representative the ancestral populations, has an effective population size of approximately 1,000,000 [11].

Beyond differences in selection strength, the disruption of chromosome architecture may also contribute to the paucity of fixed translocations in the Caenorhabditis. Gene density of course is greater in Caenorhabditis so that translocations are more likely to disrupt genes. However, intrachromosomal rearrangements are abundant, making gene density less likely to be an important factor. As noted above, each of the autosomes has distinct domains with arms and centers displaying different characteristics. Most translocations would disrupt this architecture, presumably with unfavorable effects given the conservation of the structure over time. Also Caenorhabditis chromosomes are holocentric, with a kinetochore that spreads along the chromosome's entire length [39]. Perhaps these are associated with chromosome-specific sequences, with translocations producing a hybrid signal that might interfere with normal segregation. And formally, long-range interactions of genes on the same chromosome may be important, so that the particular combinations of genes on the different chromosomes may confer a selective advantage. Finally we cannot rule out that worms are more sensitive to differences in gene dosage.

### Nonsyntenic Ortholog Pairs

Compared to syntenic ortholog pairs, the small fraction of non-syntenic pairs is unusual in having a lower percent identity. Rather than arising through translocation, these small segments presumably arose by transposition-like events, creating at least temporarily duplicate genes. These events may have occurred before the time of the MRCA, with loss of the copy in one line and loss of the original gene in the other. In this case, the lower percent identity between apparent 1:1 ortholog pairs could reflect simply the longer divergence time of the genes compared to the species divergence. If, however, the duplication/loss events occurred after the MRCA, the lower percent identity might reflect rapid adaptation of the nonsyntenic gene to its new environment. The rapid evolution might be aided by the temporary presence of two gene copies. Alternatively, perhaps only weakly conserved genes tolerate a break in synteny. Either explanation would imply a strong effect of a chromosome-wide environment, since the effect is observed independent of position along the chromosome.

### Conclusion

By using SNPs and RI lines to create a dense genetic map, we have localized much of the whole genome shotgun sequence assembly to chromosomes, with the bulk of that oriented. The C. briggsae chromosomes have an organization similar to that of C. elegans, suggesting that the distinctive features of chromosome arms and centers are functionally important over evolutionary time. Further, our analysis suggests that nematodes, perhaps like insects, are strikingly different from mammals with respect to conservation of chromosome structure and the infrequent movement of genes between chromosomes, specifically with respect to chromosomal translocations. The strong conservation of synteny indicates that chromosomal levels of selection are operating, although it is unclear what functions are being selected for or against.

## Materials and Methods

### Strains and RI lines.

C. briggsae strains were obtained from the Caenorhabditis Genetics Center. AF16 was originally isolated in Gujarat, India [40]. HK104 and HK105 were derived from collections in Okayama, Japan (H Kagawa). VT847 was collected in Hawaii, United States (V Ambrose), whereas PB800 was isolated in Ohio, United States. AF16 and VT847 group in the tropical clade of C. briggsae, whereas HK104, HK105, and PB800 group in the temperate clade [10,11].

C. briggsae recombinant inbred lines (RILs) were constructed from the AF16 and HK104 parental strains and AF16 and VT847 parental strains [10]. RILs were constructed from F2 progeny of crosses between HK104 (or VT847) males and sperm-depleted AF16 hermaphrodites. F2 larvae were picked as L4s and propagated through one hermaphrodite per generation from F2 to F11.

### Library construction and sequencing.

Genomic DNA was prepared from each of the strains [41]. The DNA was sheared, sized-selected, ligated into the pOT sequencing vector, and transformed into competent cells as described [42]. The resultant colonies were used to prepare plasmid DNA, which was sequenced as described [42].

### Sequence assembly terminology.

The several levels of sequence assembly are defined as follows. Sequence contigs are assembled from overlapping sequence reads with no gaps. Supercontigs are constructed by linking contigs using read-pair information to span a gap. In turn, fpc contigs were constructed by aligning the supercontigs, where possible, to the clone-based physical map, and using the physical map continuity to link and orient supercontigs with respect to one another. We use “sequence assemblies” where it is not important to distinguish the different levels. The acronym “fpc” or FingerPrint Contigs is derived from the program fpc used in physical map construction (Soderland et al. 1997).

### Genetic map.

SNP discovery/alignment methods: Each of the reads was initially aligned against the C. briggsae genome sequence, using WU-BLASTN (S = 1000, S2 = 150, W = 13, gapW = 4, gapS2 = 150, M = 5, N = −11, Q = 11, R = 11, B = 10000, V = 10000, hspmax = 1000) [43]. The alignments were then filtered for alignments over 100 bases long and greater than 96% identity. The top alignments by *p*-value were then re-aligned using CROSSMATCH (P. Green, unpublished data) using the following parameters: -masklevel 0, –alignments, –discrep_lists. Discrepancies with quality values higher than 35 were then mapped backed to the C. briggsae genome.

Marker selection and primer design: Design of FP-TDI genotyping assays was attempted for all putative SNPs in high-throughput fashion as previously described [44]. Flanking sequences were extracted from the *cb25.supercontigs.fasta* assembly and masked for repetitive elements with RepeatMasker, using a customized library of C. briggsae repeats. However, the positions of nearby putative SNPs were not marked. PCR primers for the optimal melting temperature (54–56) and product size (80–400 bp) were identified using *Primer3* [45]. For each SNP that passed PCR primer design, Perl scripts identified the shortest extension primer of 16–40 bp with TM of 50–55. If a suitable extension primer was not found in forward orientation, design on the reverse strand was attempted.

Supercontigs in the C. briggsae *cb25.supercontigs.fasta* whole-genome assembly with at least one assayable HK104 putative SNP were sorted by size from largest to smallest. One or two markers were selected for each supercontig until a total of 400 SNPs was reached. For supercontigs with more than two available SNPs, the markers with the lowest and highest contig positions were selected.

FP-TDI: The SNPs were genotyped using the template-directed dye-terminator incorporation (FP-TDI) assay as previously described [46,47]. The FP-TDI assay required three unlabeled oligonucleotides for each SNP. Two served as PCR primers and the third was a SNP probe that was complementary to the template sequence with its 3′ end annealed to the target one base before the polymorphic site. The entire reaction was conducted in single reaction tube without separation or purification. The DNAs from the two RI line crosses were assembled in two 96-well trays including parental DNAs (each duplicated as controls), and two no-DNA controls. The FP-TDI experiments were conducted in a 384-well plate format, typing two SNPs against the DNAs. .

Kits (AcycloPrime-FP, Perkin Elmer Life Sciences, http://www.perkinelmer.com) were used for FP-TDI. Briefly, after a PCR step using a hot start Taq polymerase and two designed primers, Exonuclease I and shrimp alkaline phosphatase were added to digest remaining primers and inactivate deoxynucleotide triphosphates, and the enzymes were heat inactivated at the end of the digestion. For the TDI step, also called primer extension or minisequencing, the designed SNP primer, Taq polymerase from the kit, buffer, and the appropriate combination of dye terminators labeled with TAMRA or R110 dye were added and the samples were subjected to a thermocycling program. We detected incorporation of the dyes by measuring fluorescent polarization (EnVision, Perkin Elmer Life Sciences). We further used quenching properties of the dyes to aid in scoring genotypes [48].

Genetic map construction: After quality control for genotyping, the genotypes, classified by SNP and RI line, were assembled in a text file. Using this text file, the genetic map was assembled as described in results using the program Map Manager QTXb20 (http://www.mapmanager.org/) [15].

To confirm the order of chromosome CbX, single F2 worms, which were provided to us by Bhagwati Gupta (McMaster University),were isolated from an AF16 x HK104 cross and placed in lysis buffer. We performed whole-genome amplification on each sample using a kit containing Phi 29 DNA polymerase according to the manufacturer's instructions (GenomiPhi, GE Healthcare, http://www.gehealthcare.com). Some 95 animals typed with 11 markers on CbX were used to generate a new version of the genetic map using Map Manager QTXb20. The results were consistent with version 3.3 (unpublished data).Details of the genetic map are available (Dataset S1, http://snp.wustl.edu/, and http://www.wormbase.org/).

Comparison to other genetic maps: Genes with molecular correlates in the current classical genetic map (Bhagwati Gupta, personal communication) were identified and placed on the C. briggsae integrated assembly. In turn, the C. elegans ortholog was identified along with its chromosomal location.

No significant differences arose in comparison of these maps with the integrated map derived here.

### Sequence/genetic map integration.

Methods for breaking sequence assemblies: For each assay the three markers were independently aligned to the genome sequence using WU-BLASTN, selecting the site with all three markers at expected intervals. For those sequence assemblies assigned to multiple linkage groups, we identified the interval where a transition occurred in the chromosomal assignment in groups of genes identified by 1:1 orthology (see Methods below). We located any gaps between supercontigs in the interval (usually only one) and split the sequence assembly at that point, assuming there had been a false join. In the few instances where more than one gap lay in the interval, other alignments were used to determine the most likely site of the false join. Sequence assemblies were only broken when genetic mapping data dictated the break.

Defining order/orientation: Sequence assemblies were localized to chromosomes and then to locations along those chromosomes based on the genetic positions of the assigned markers. Similarly, sequence assemblies were oriented based on the genetic position of multiple assigned markers. For adjacent ultracontigs where the genetic markers had identical genetic map positions, read-pairing data from the underlying whole-genome shotgun assembly were used where possible to assign order. Also for ultracontigs where the genetic markers did not establish orientation, we used read-pairing data with neighboring ultracontigs where possible to orient them.

Rules for placing on Chr*_random: For those sequence assemblies remaining unlocalized after using the genetic mapping data, we assigned them to a specific chromosome in the Chr*_random bin if that assembly had at least six 1:1 orthologs (defined as below) on the majority chromosome and no more than four and less than 15% assigned to the secondary chromosome. The remainder were left on “chrUn”.

### Chromosome analysis.

Center versus arm boundaries in C. elegans and C. briggsae: We created recombination plots (genetic versus physical location) for both C. elegans and C. briggsae. From those data, we identified the inflection points that delineate central cluster region from the arms for both species (Table 6).

Gene sets: We used both the C. briggsae hybrid gene set [8] obtained from WormBase (versions brigpep2.pep/cb25.hybrid.gff) and a set of genes based on homology with C. elegans confirmed genes (L. Hillier and R. Waterston, unpublished data) in our analyses. We mapped these genes onto the coordinates of our modified assembly and integrated genome sequence.

For the exon and intron density plots and for all 1:1 ortholog calculations reported here, we used the hybrid gene set, whereas we used the alternative set for refining breakpoints in the fpc contigs as described. We obtained the C. briggsae integrated hybrid gene set [8] from WormBase (versions brigpep2.pep/cb25.hybrid.gff) and mapped that set to the new C. briggsae coordinates.

For C. elegans, we created a nonredundant set of C. elegans genes from WormBase release 137 by retaining the longest gene per transcript for those with multiple transcripts per gene.

Defining the C. elegans:C. briggsae orthologs and ortholog blocks: To define the C. elegans:C. briggsae 1:1 orthologs, for both the C. elegans gene set and the C. briggsae hybrid gene set we searched each gene set against itself and against each other using WU-BLASTP in two rounds first using (filter = seg, V = 10000, B = 10000, hspmax = 10000, -topcomboN = 1) and then rerunning the analyses removing filter = seg. Using the results from the WU-BLASTP with filter = seg, a gene was labeled as unique (“1”) if the best hit against its own protein set had a P-value exponent at least 29 larger than the P-value of the next best hit. We then examined the between-species matches. To qualify a match as a C. elegans:C. briggsae 1:1 ortholog, we required (a) that a *p*-value be at least as significant as 1× 10^−09^ between the sets, (b) that the gene be a “1” in C. elegans and a “1” in C. briggsae, (c) that the proteins be mutual best similarities, (d) that the top match was better by 10^−29^than the second best match and (e) at least 50% of the C. briggsae protein must align to at least 50% of the C. elegans protein. For requirements a, b, c, and d, the WU-BLASTP results using filter = seg were used. For requirement (e), the WU-BLASTP results that were obtained not using filter = seg. We defined a syntenic ortholog as one localized to the same chromosome in both C. elegans and C. briggsae; a nonsyntenic ortholog was defined as one localized to different chromosomes. For a subset of the analyses, we removed clusters of more than three nonsyntenic orthologs.

To define an ortholog block, we identified stretches of C. briggsae sequence where the C. elegans genes were on the same chromosome and in the same order as those in C. briggsae allowing only a single “out of order” C. elegans gene to interrupt a block and allowing no more than two C. elegans genes to be “missing”/moved.

Repeats Repeatmasker [49] was run using the C. briggsae repeat library [8] to identify repeats in C. briggsae. For *C. elegans,* the repeat boundaries were downloaded from WormBase (release 137).

## Supporting Information

Dataset S1 Raw Marker Data Provides the raw allele determination for each of the 321 scored markers across the 93 strains.
(477 KB XLS)

Dataset S2 Genome Map Data Provides the set of markers ordered along each of the six linkage groups, the fraction of each allele called for each marker, the position of the markers within the sequence assemblies and the chromosome, and the order and orientation of the sequence assemblies along the chromosome and the primer sets. Graphs of the AF16 allele fraction for each chromosome and Marey plots for each chromosome are included.
(213 KB XLS)

Dataset S3 Distribution of Features across C. briggsae Chromosomes The representation of various features is given in 500-kb windows in 100-kb steps across each chromosome. Features include the percentage of the sequence in the window that is intronic, the percentage that is exonic, and the percentage that is repetitive. Also shown are the percentage of exons in orthologs and the number of telomere repeat sequences in each 500-kb window.
(105 KB XLS)

Dataset S4 Distribution of Features across C. elegans Chromosomes Identical to Dataset S3 except for data are provided for C. elegans rather than C. briggsae.
(112 KB XLS)

Dataset S5 Ortholog Positions The positions of 1:1 orthologs in both C. elegans and C. briggsae.
(1.7 KB XLS)

Figure S1 Distribution of Features across C. briggsae Chromosomes Graphs of each feature for each chromosome corresponding to the data provided in Dataset S3.
(140 KB PDF)

Figure S2 Distribution of Features across C. elegans Chromosomes Graphs of each feature for each chromosome corresponding to the data provided in Dataset S4.
(151 KB PDF)

Figure S3 Ortholog Positions Graphical representations of the positions of 1:1 orthologs in both C. elegans and C. briggsae. Graphical representations are provided for all on-chromosome relationships.
(150 KB PDF)

Figure S4 Recombination Data Plot of the physical versus genetic map positions for C. elegans.
(243 KB PDF)

### Accession Numbers

This Whole Genome Shotgun project has been deposited at DDBJ/EMBL/GenBank (http://www.ncbi.nlm.nih.gov/Genbank/) under the project accession CAAC00000000. The version described in this paper is the first version, CAAC01000000. Accession numbers for the C. briggsae chromosomal sequences are: CU457376, CU457377, CU457378, CU457379, CU457380, and CU457381. The chromosomal assembly is also available at http://www.wormbase.org as “C. briggsae build CB3.”

## Abbreviations

cM: centimorgans
fpc contigs: physical map–based contigs
FP-TDI: fluorescent polarization detection
kb: kilobases
Mb: megabases
MRCA: most recent common ancestor
*N_e_*: population size
RI: recombinant inbred
SNP: single nucleotide polymorphism
