# Supplementary figures and images for: Comparison of C. elegans and C. briggsae Genome Sequences Reveals Extensive Conservation of Chromosome Organization and Synteny
Source: PLoS Biol. 2007 Jul 3;5(7):e167. doi: 10.1371/journal.pbio.0050167 (PMC1914384; doi:10.1371/journal.pbio.0050167)

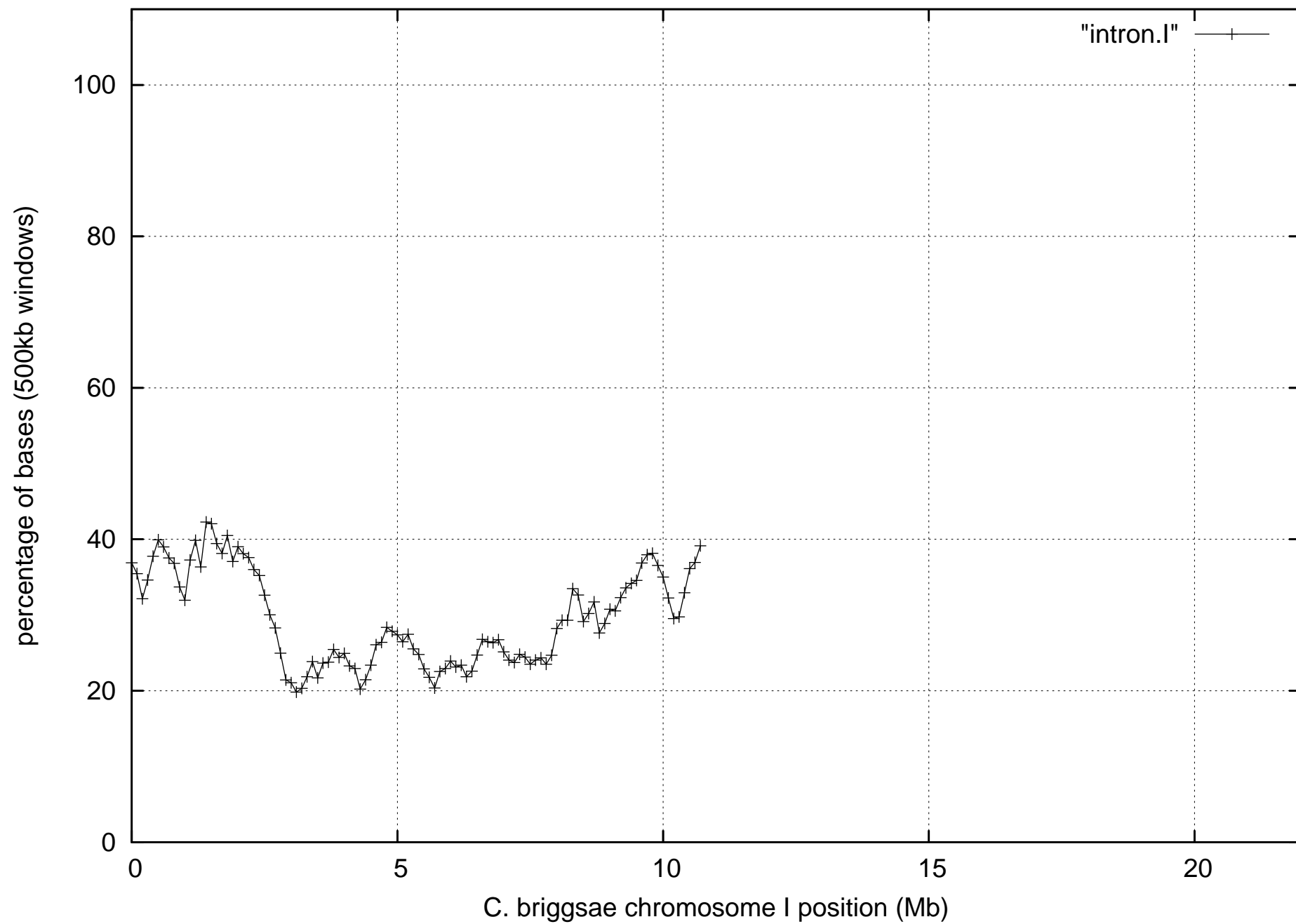

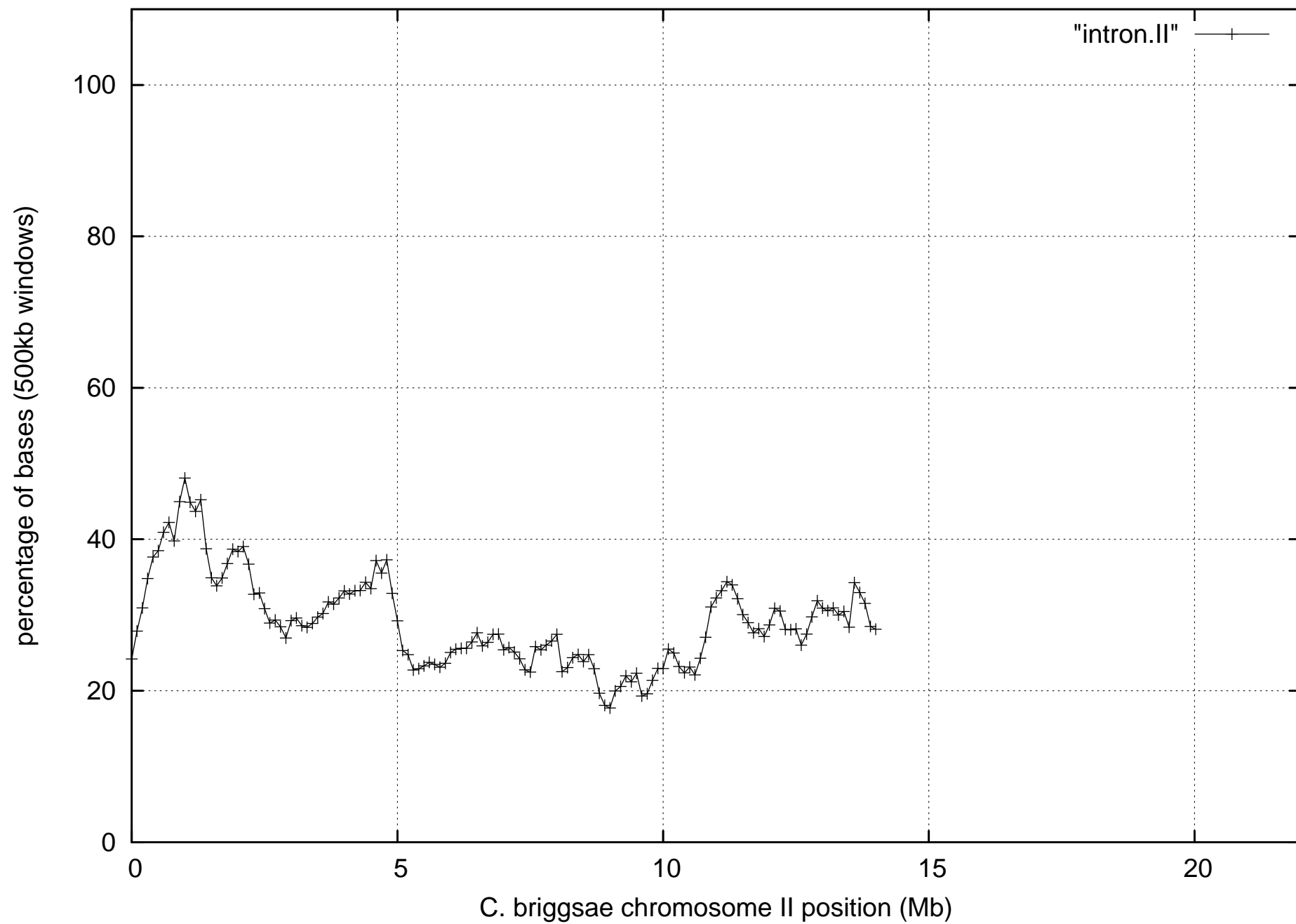

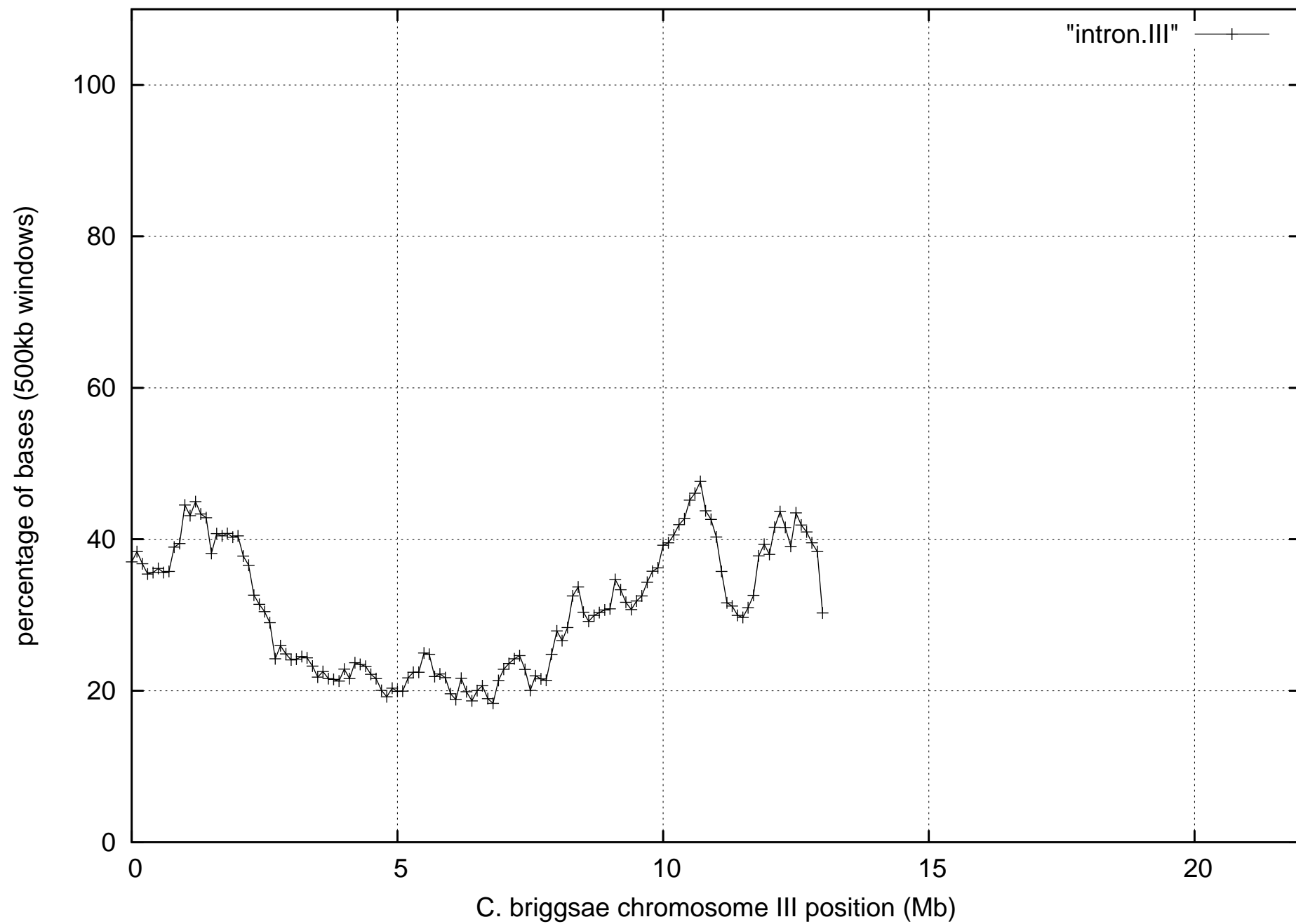

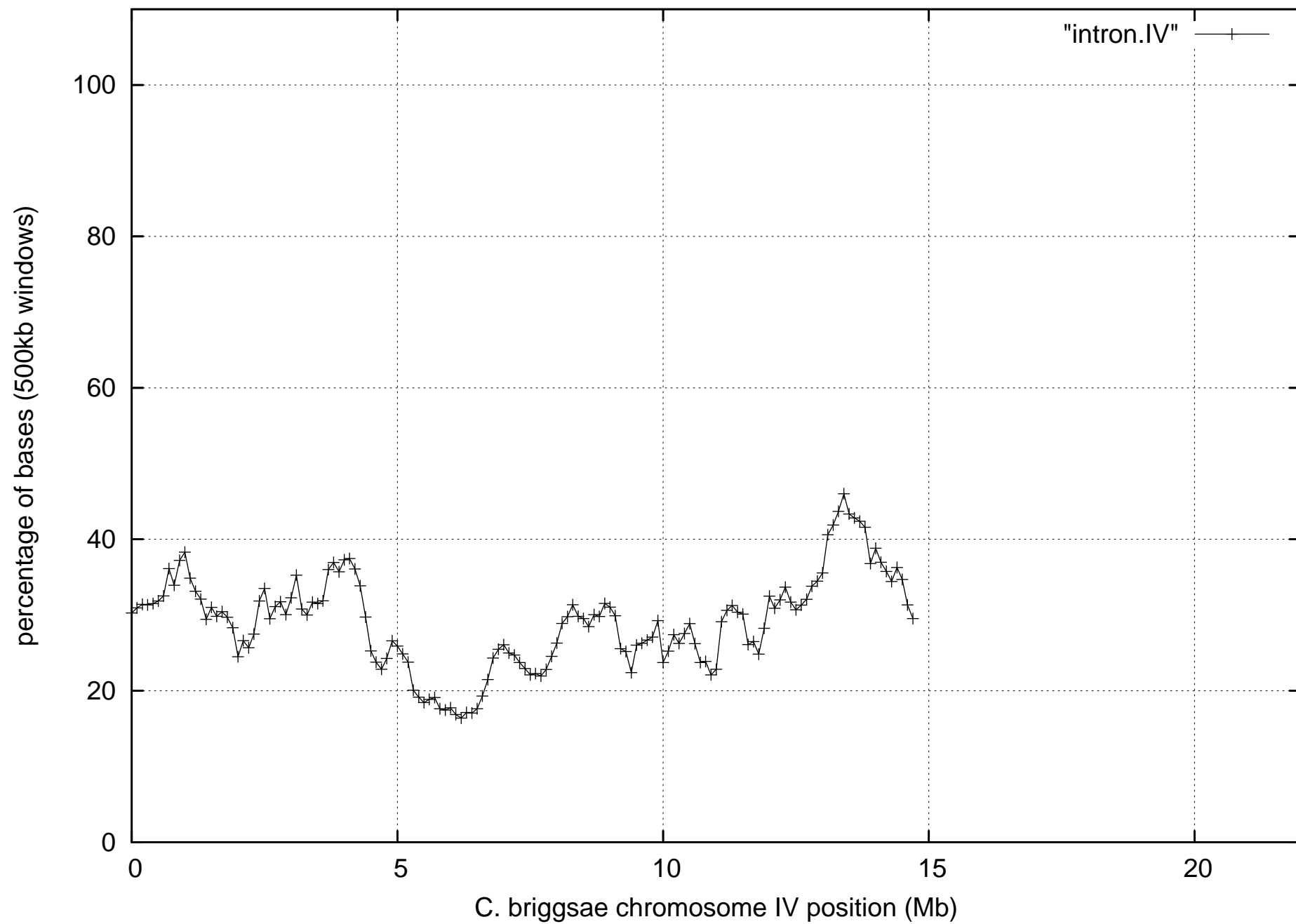

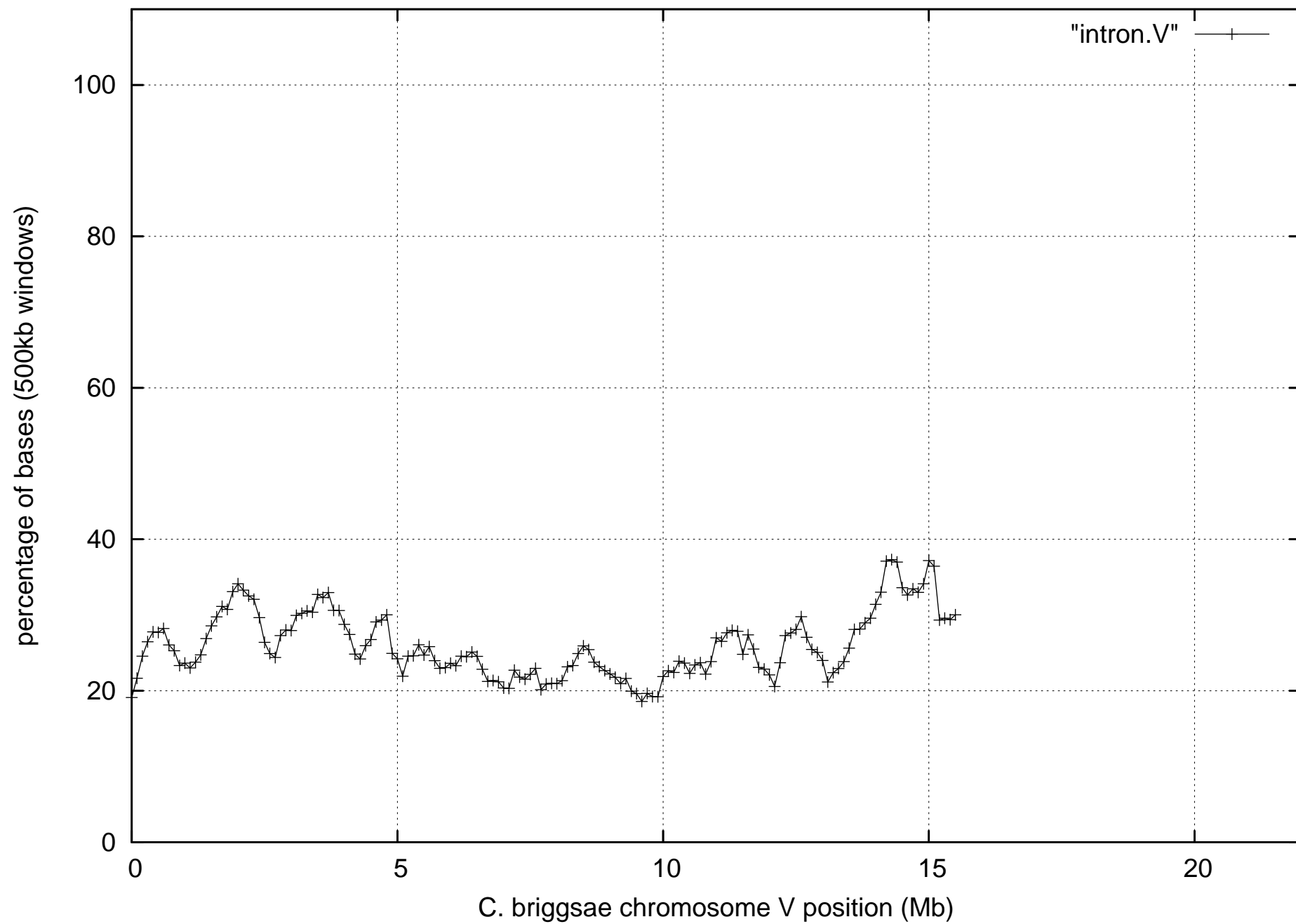

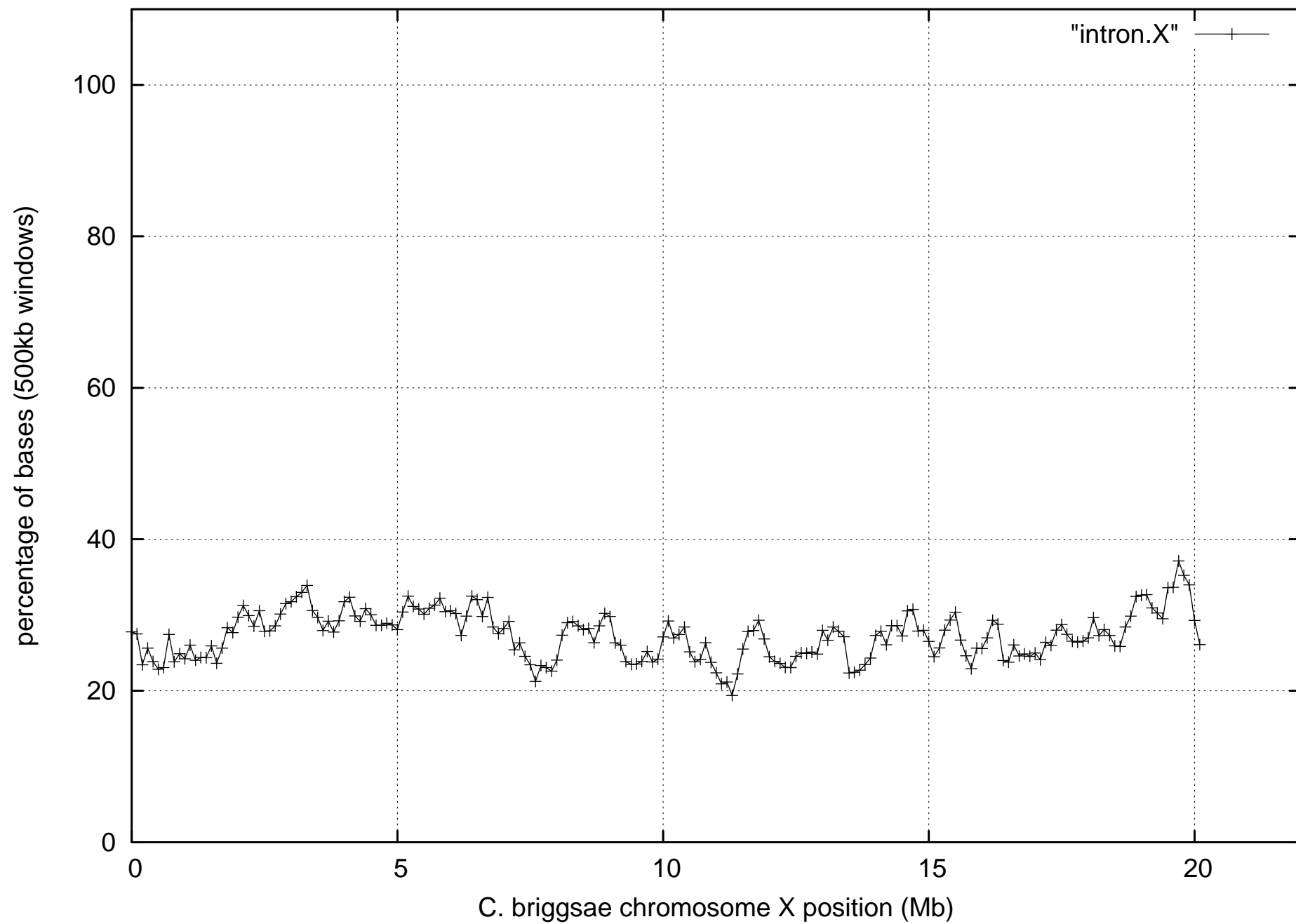

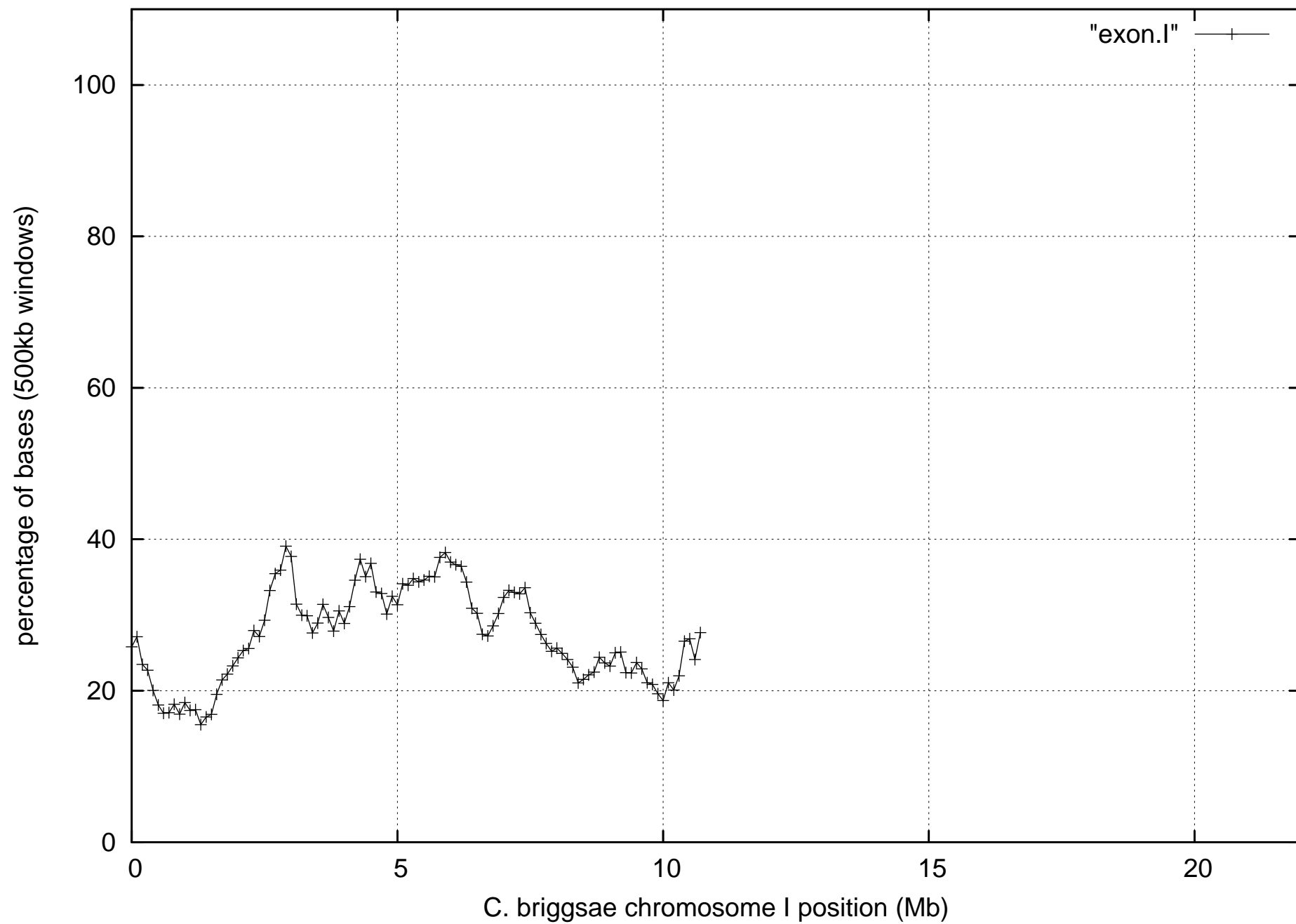

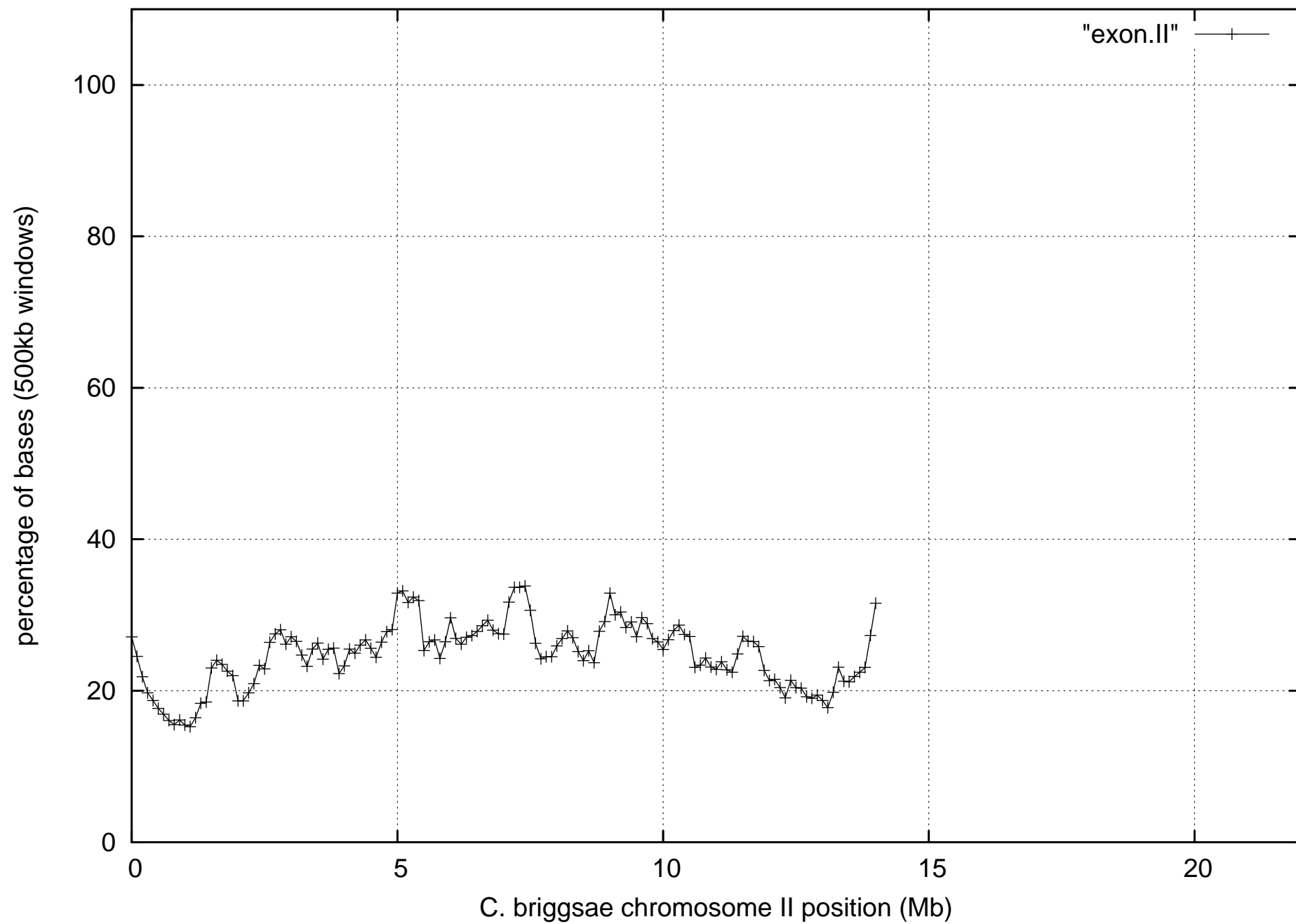

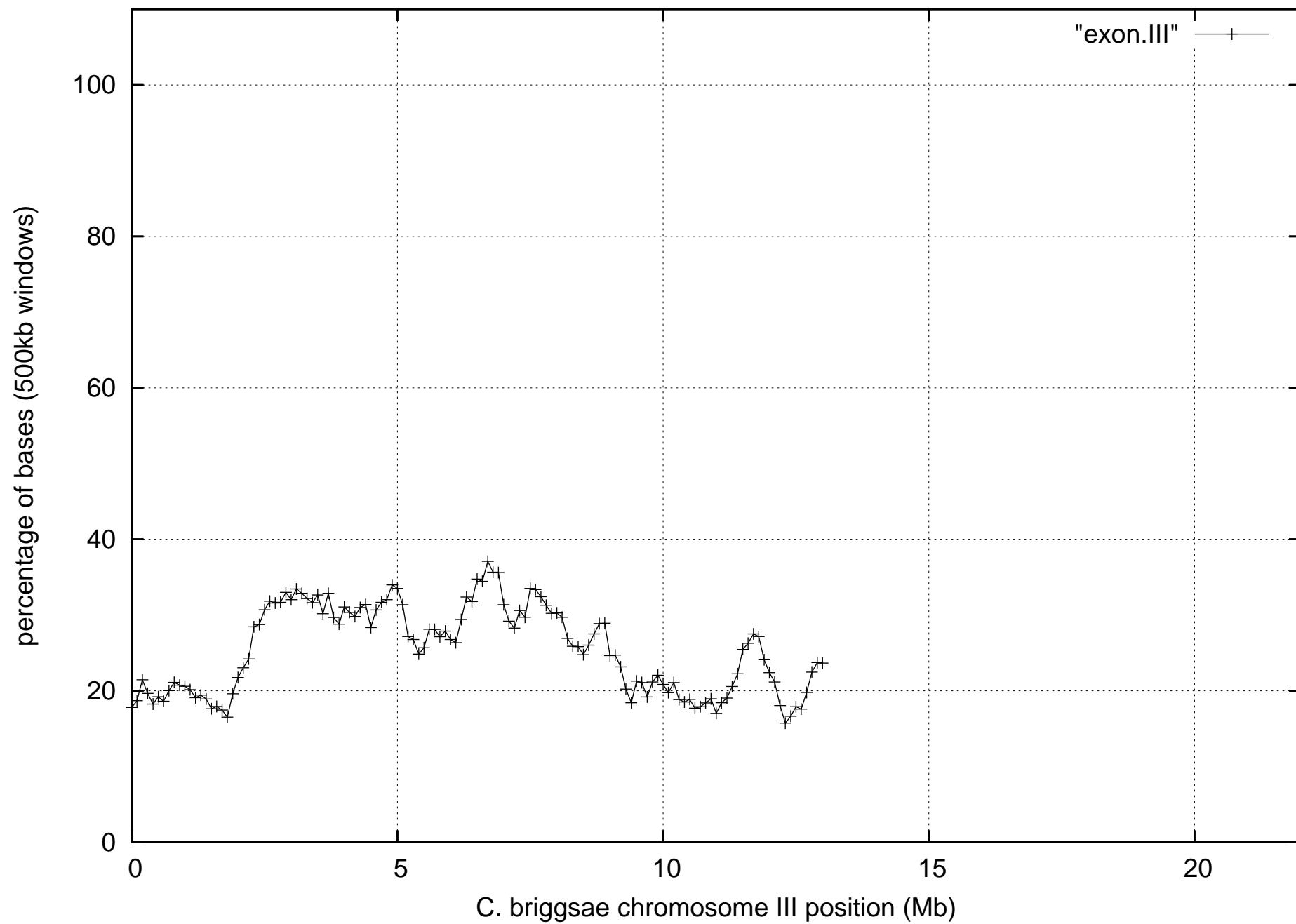

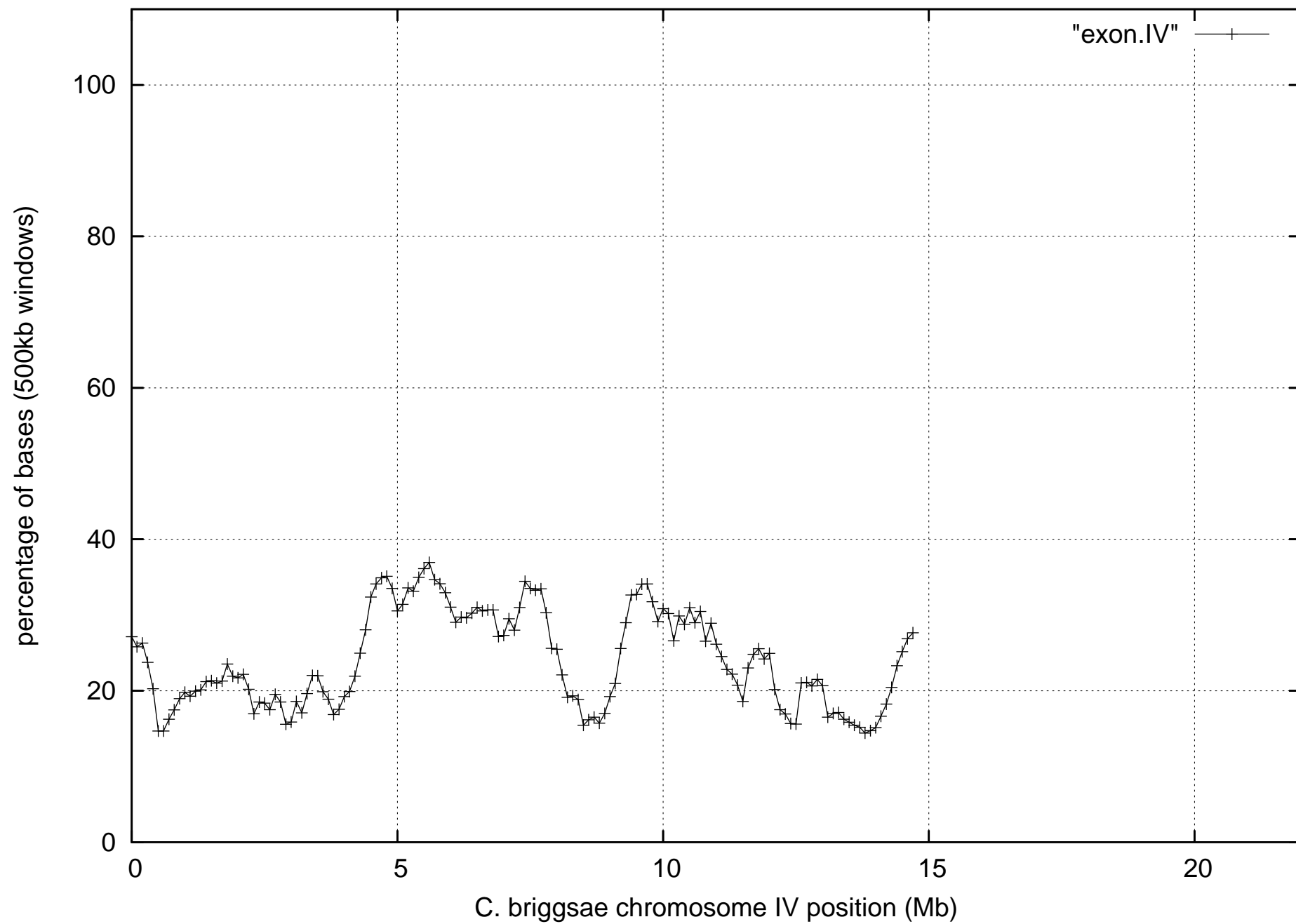

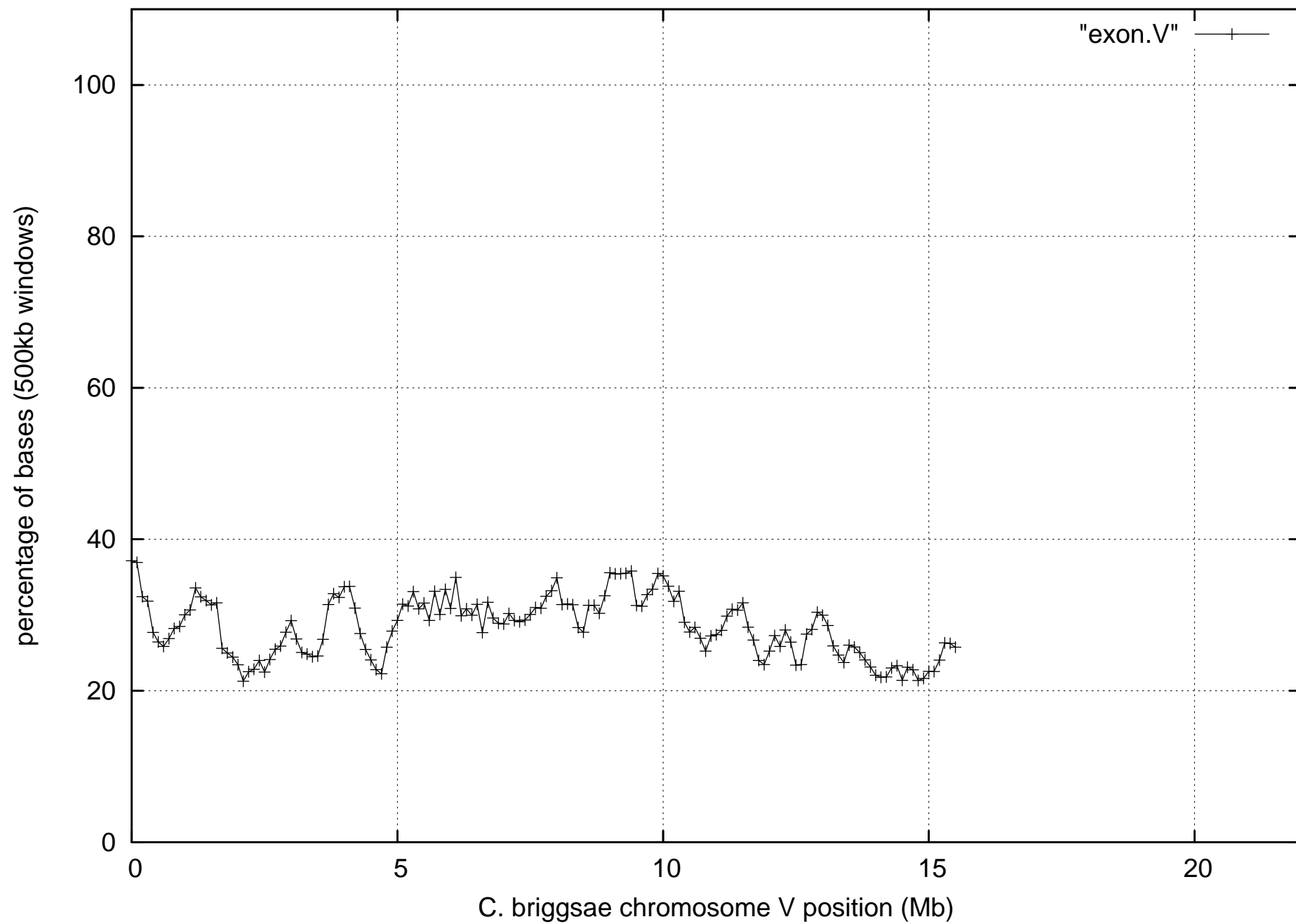

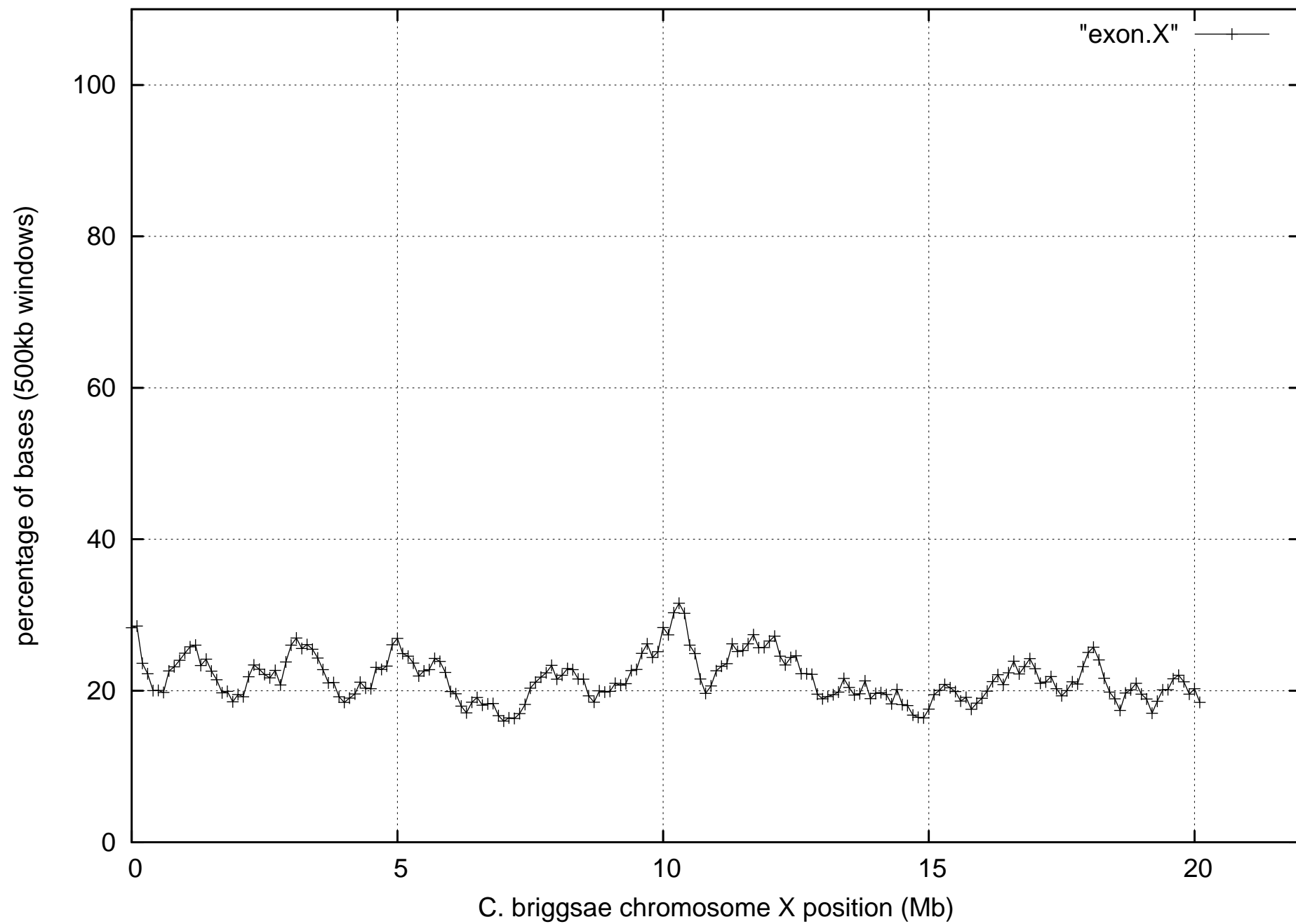

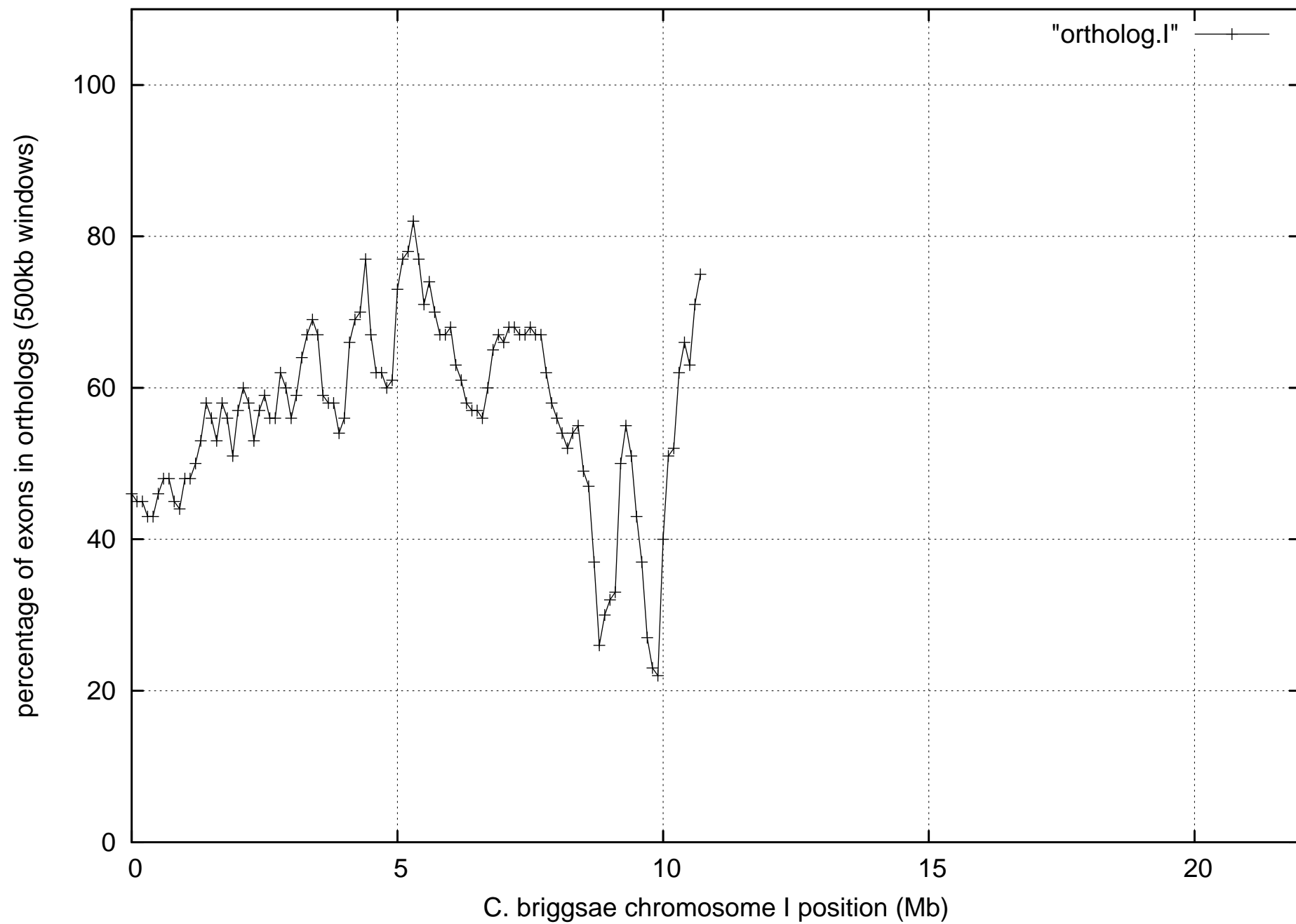

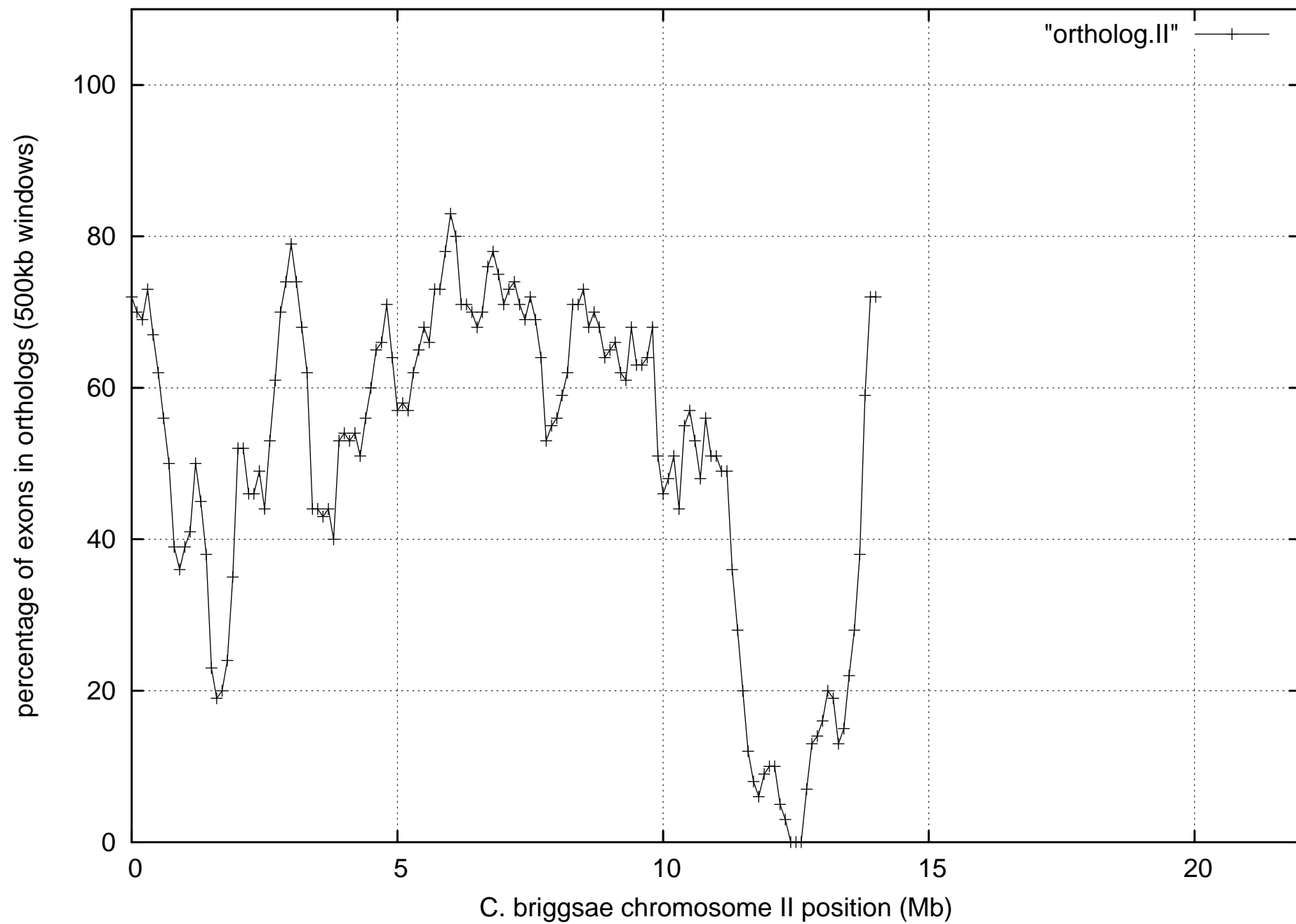

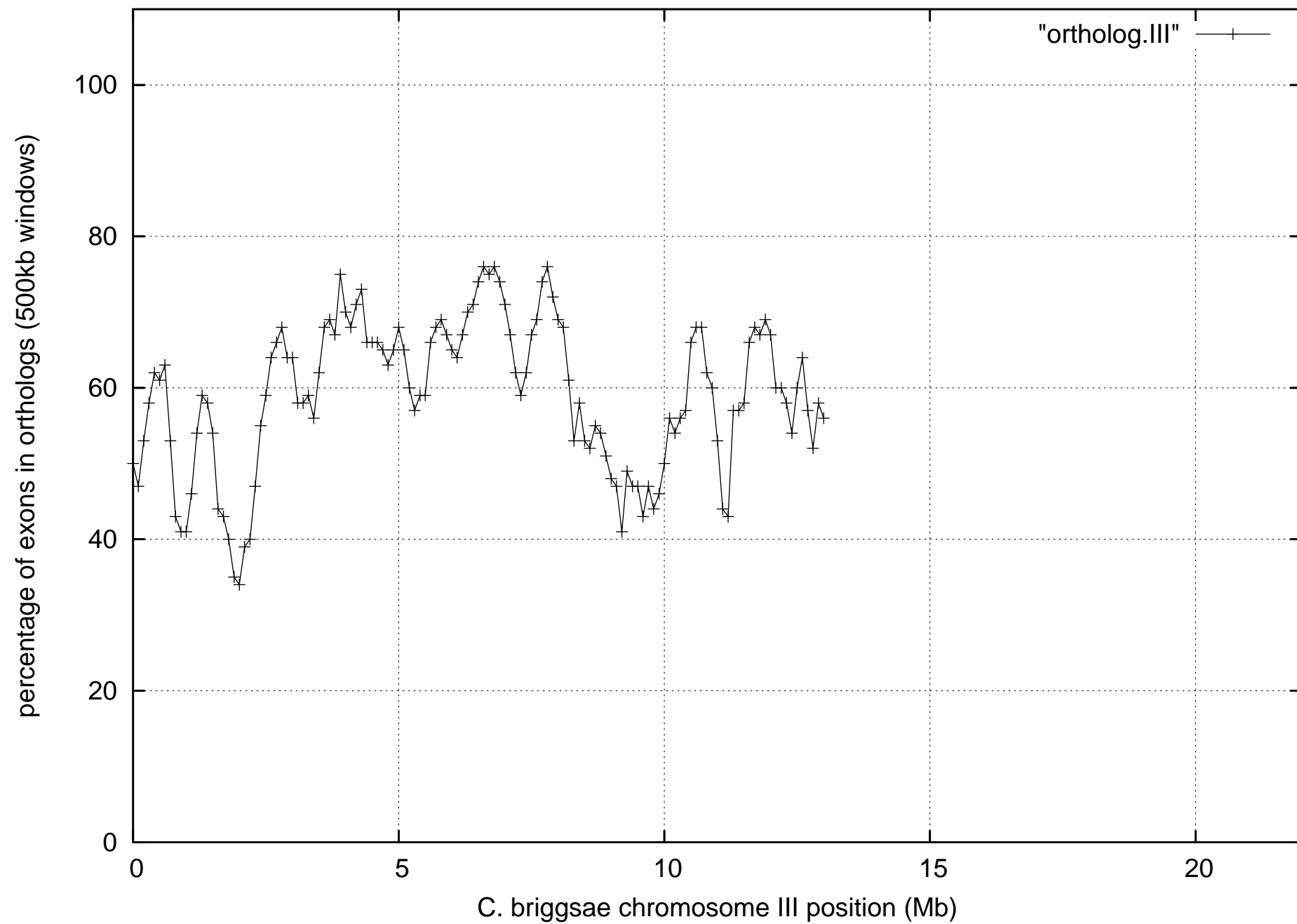

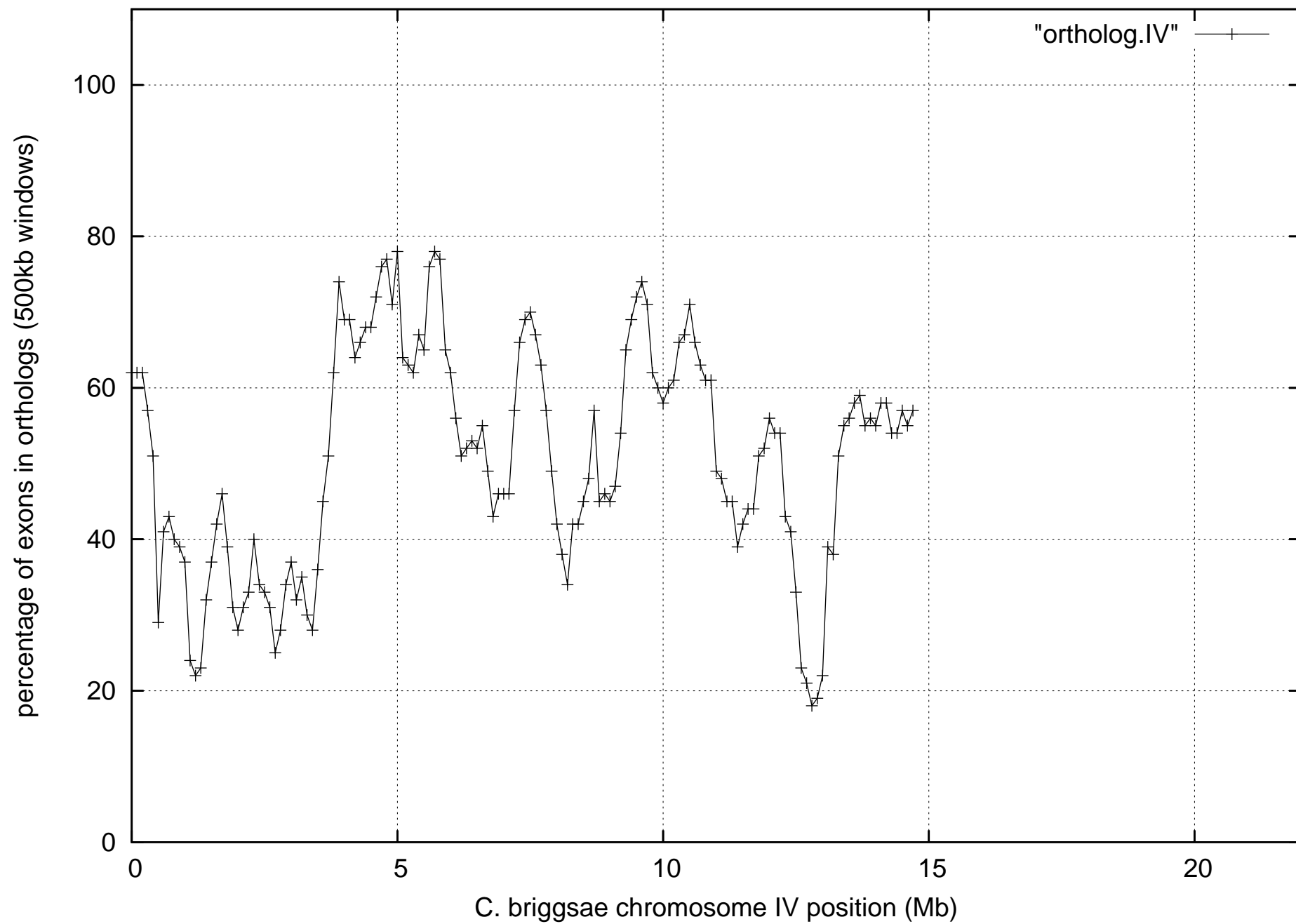

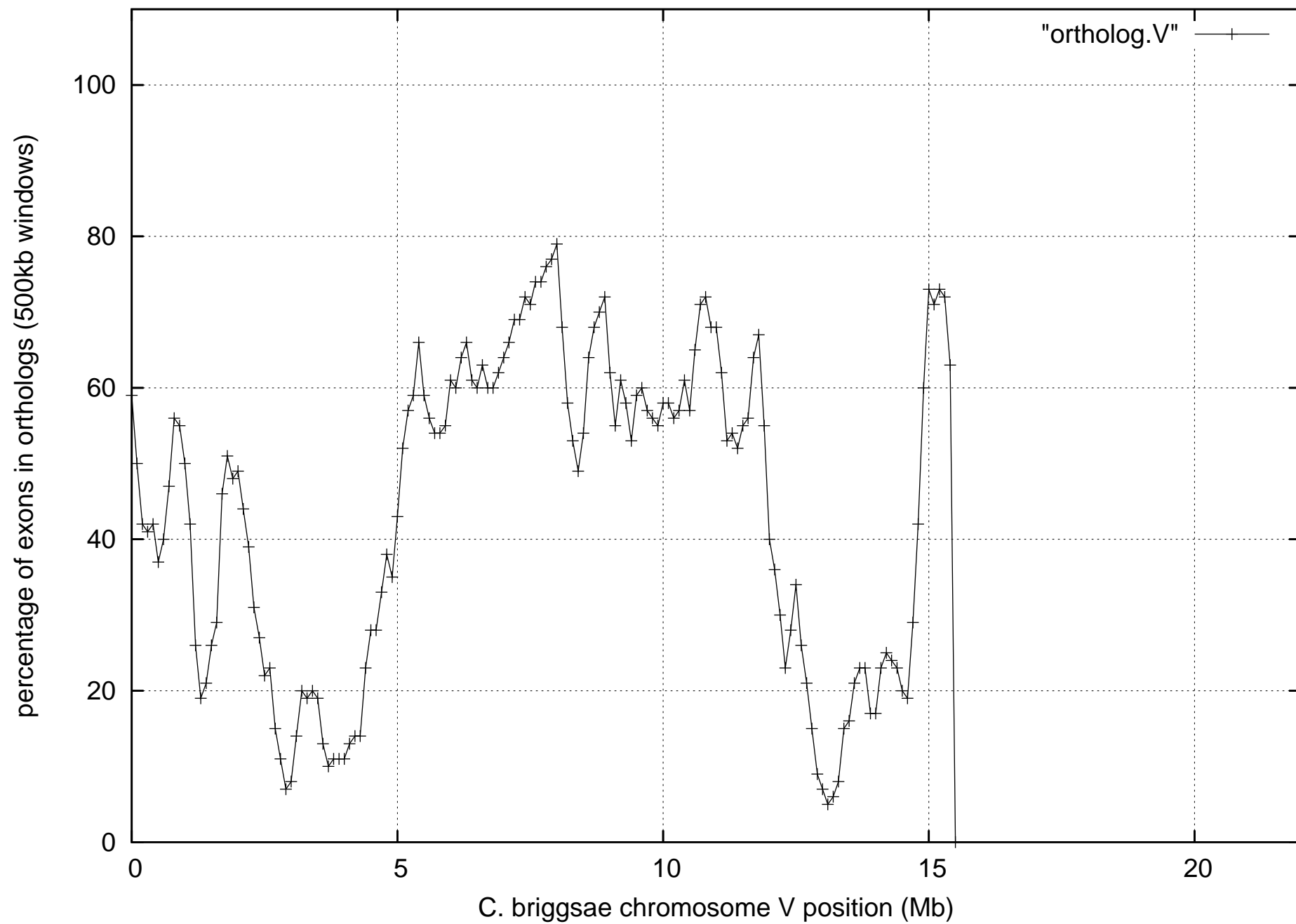

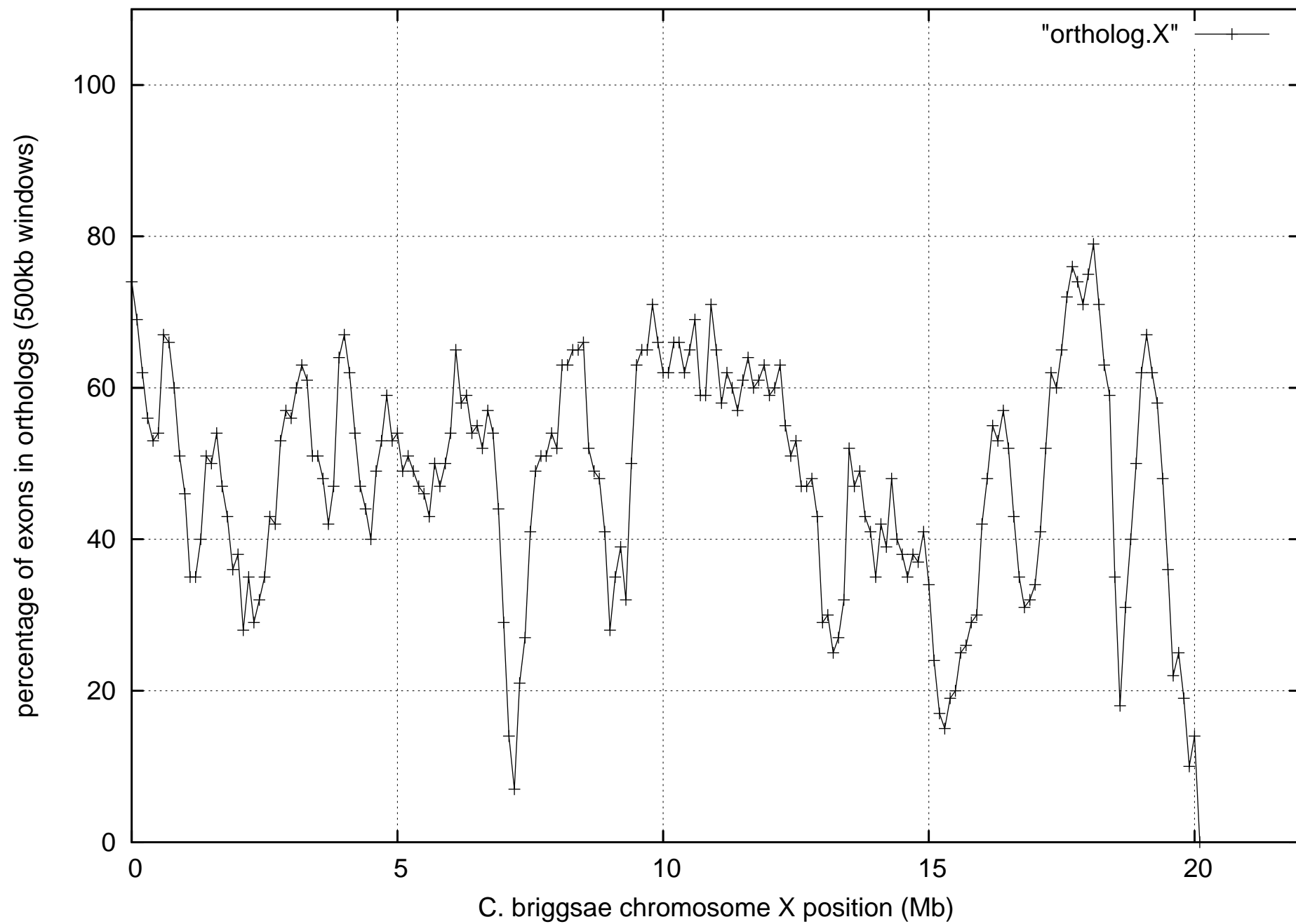

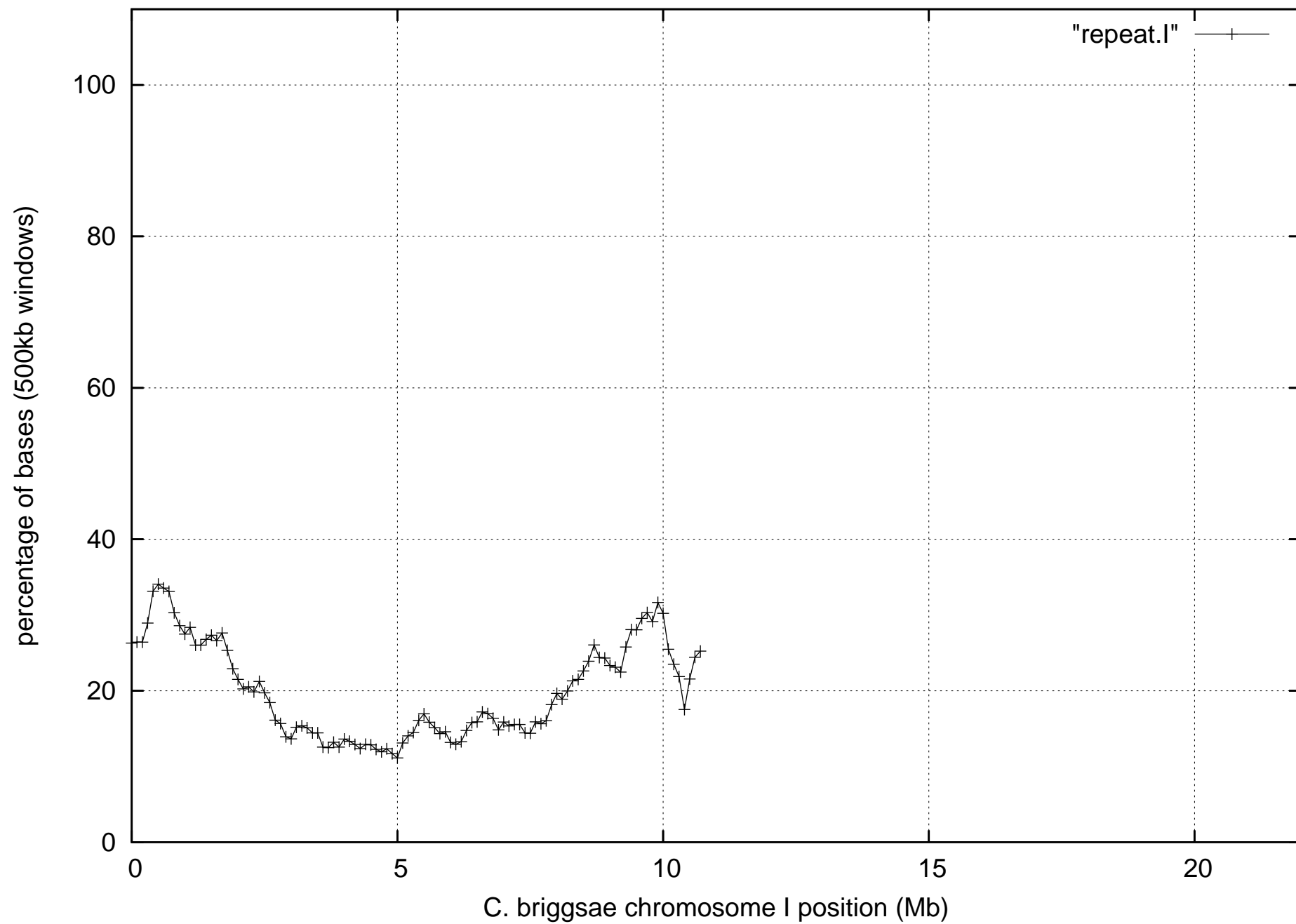

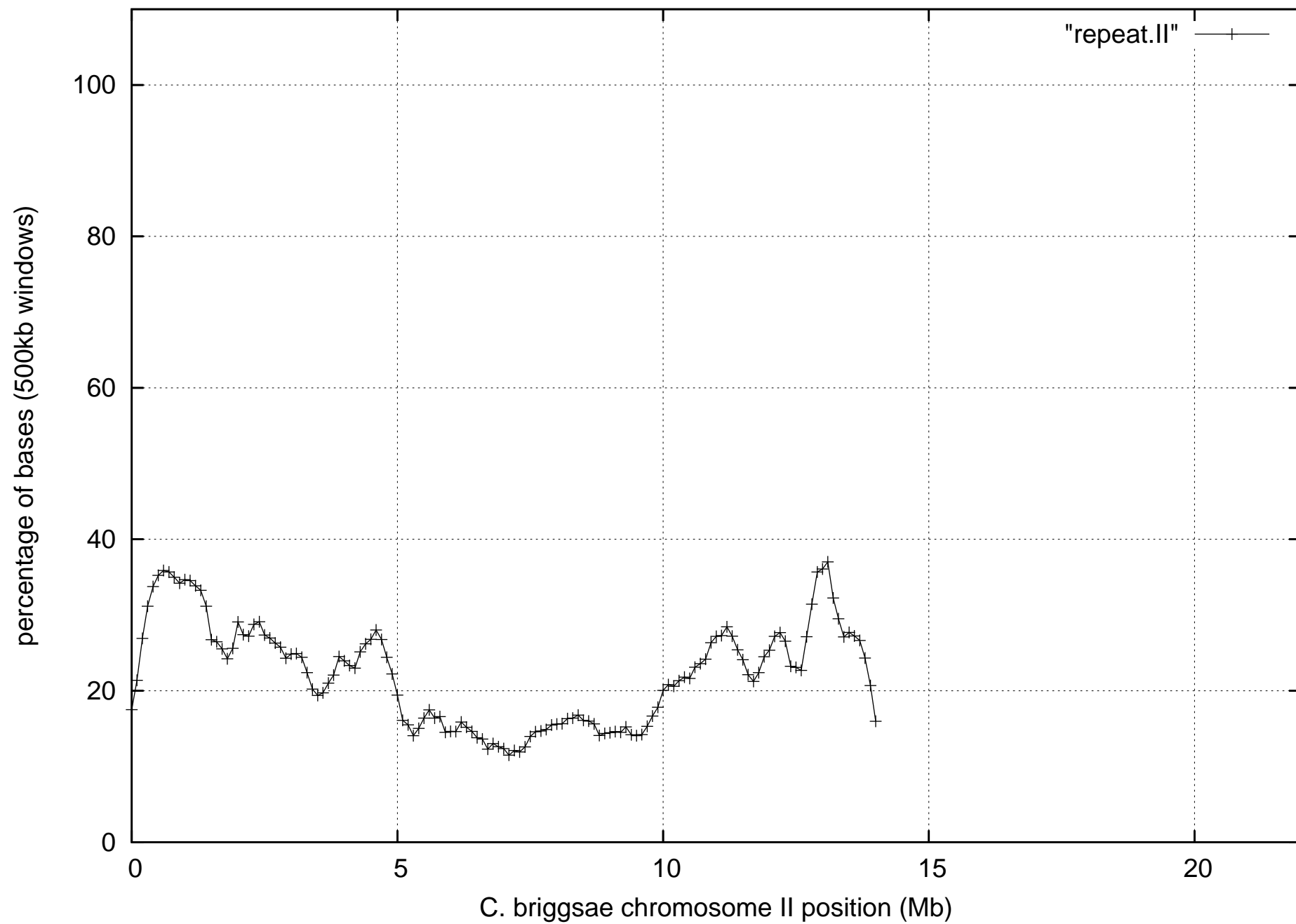

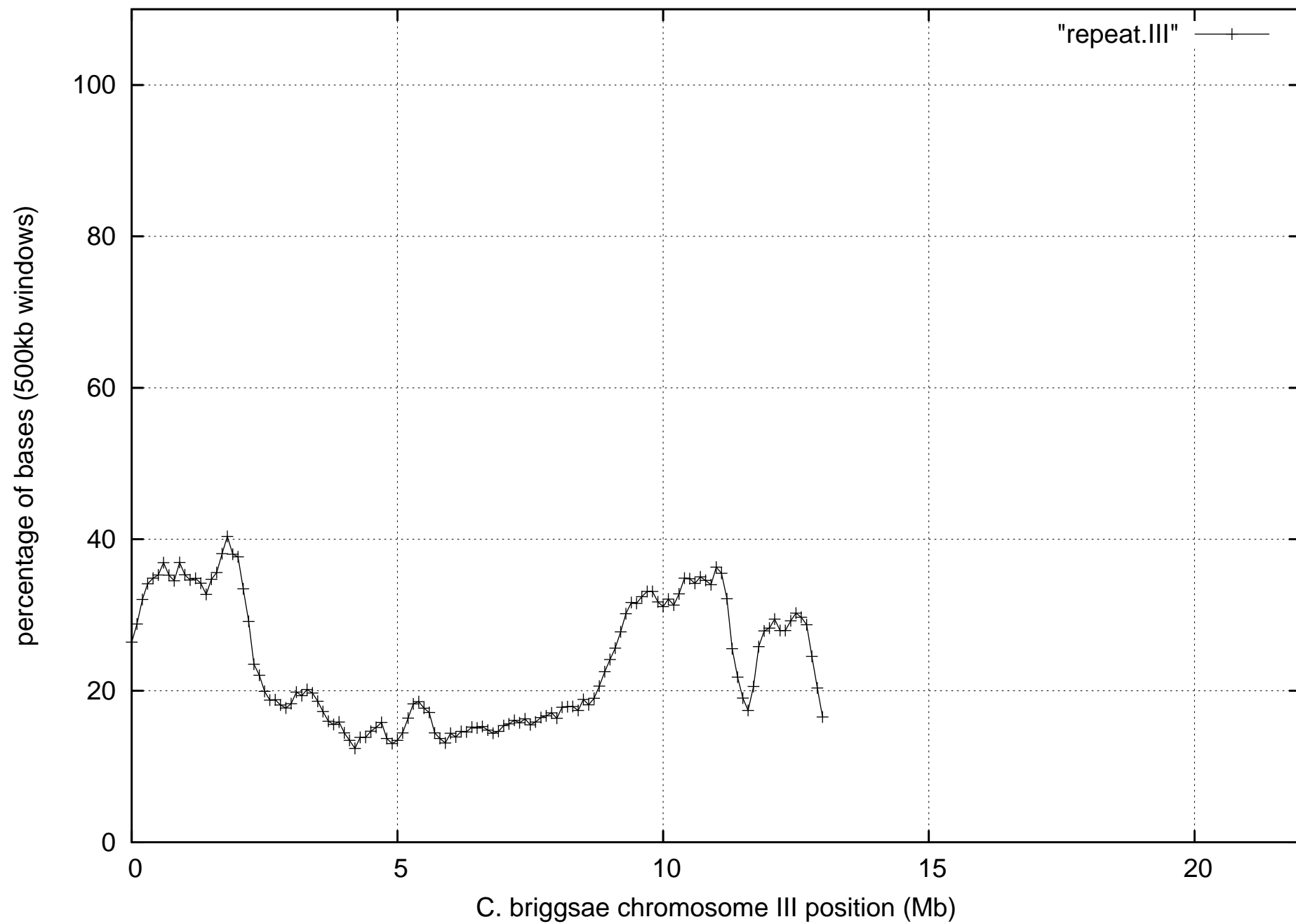

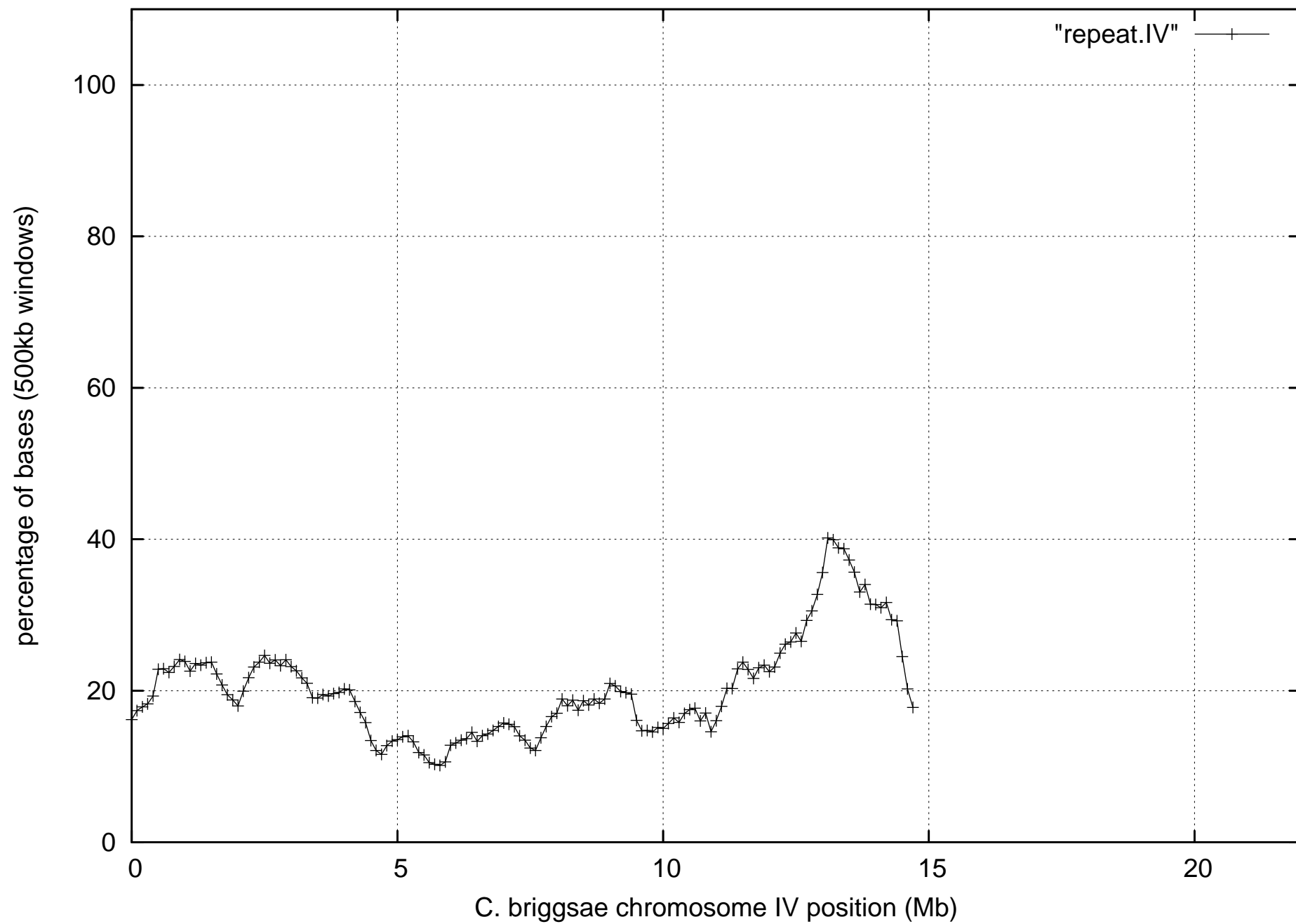

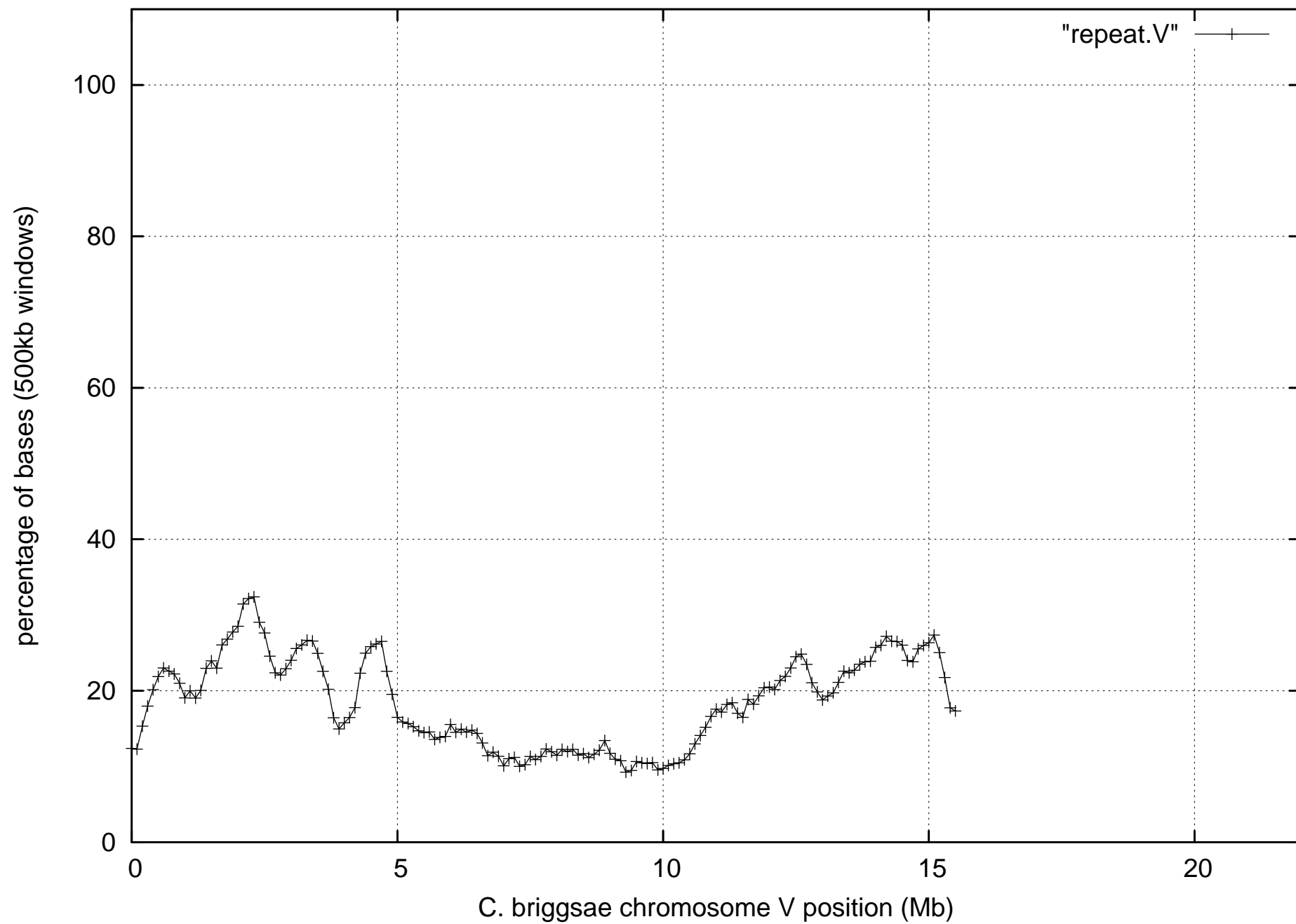

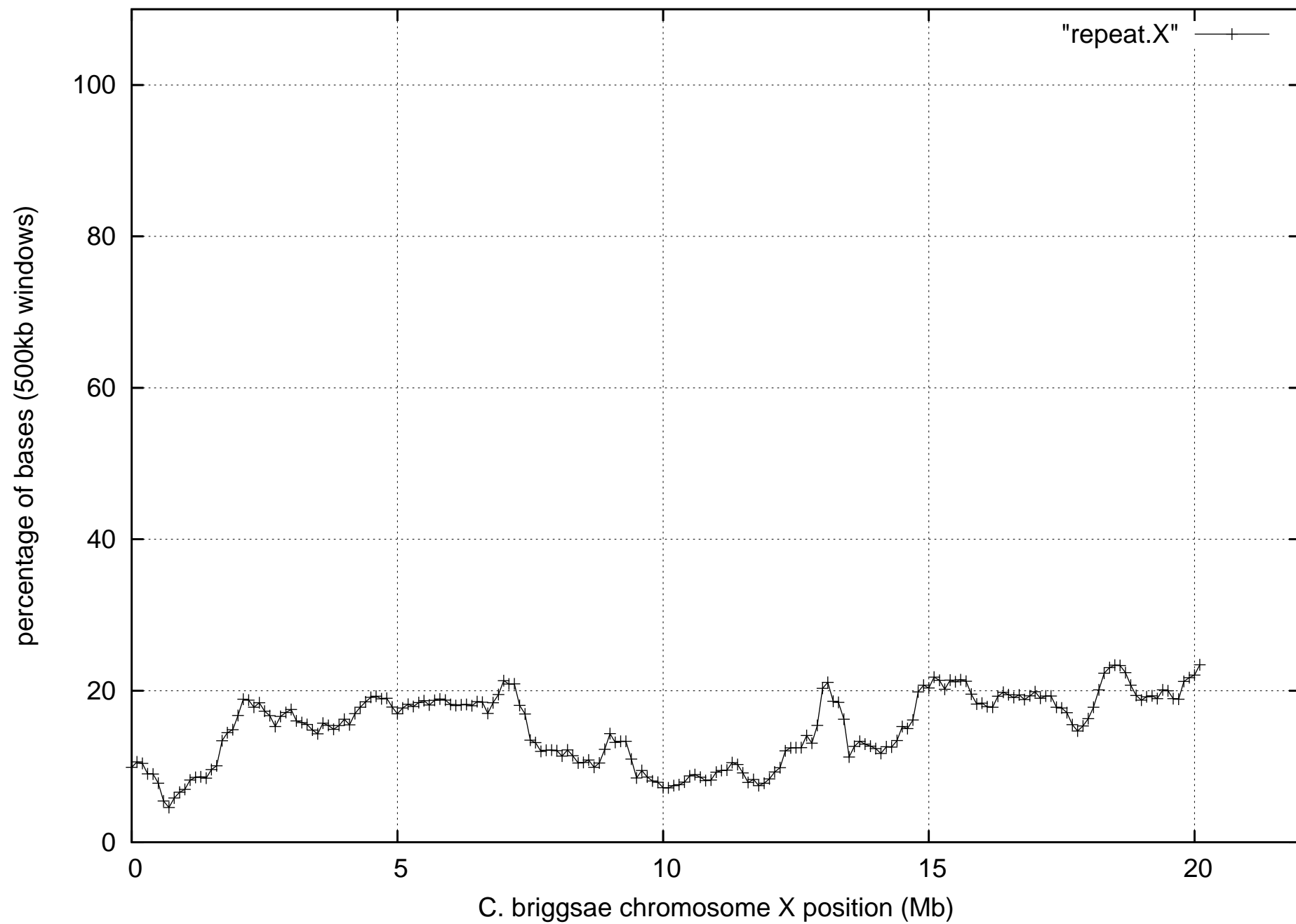

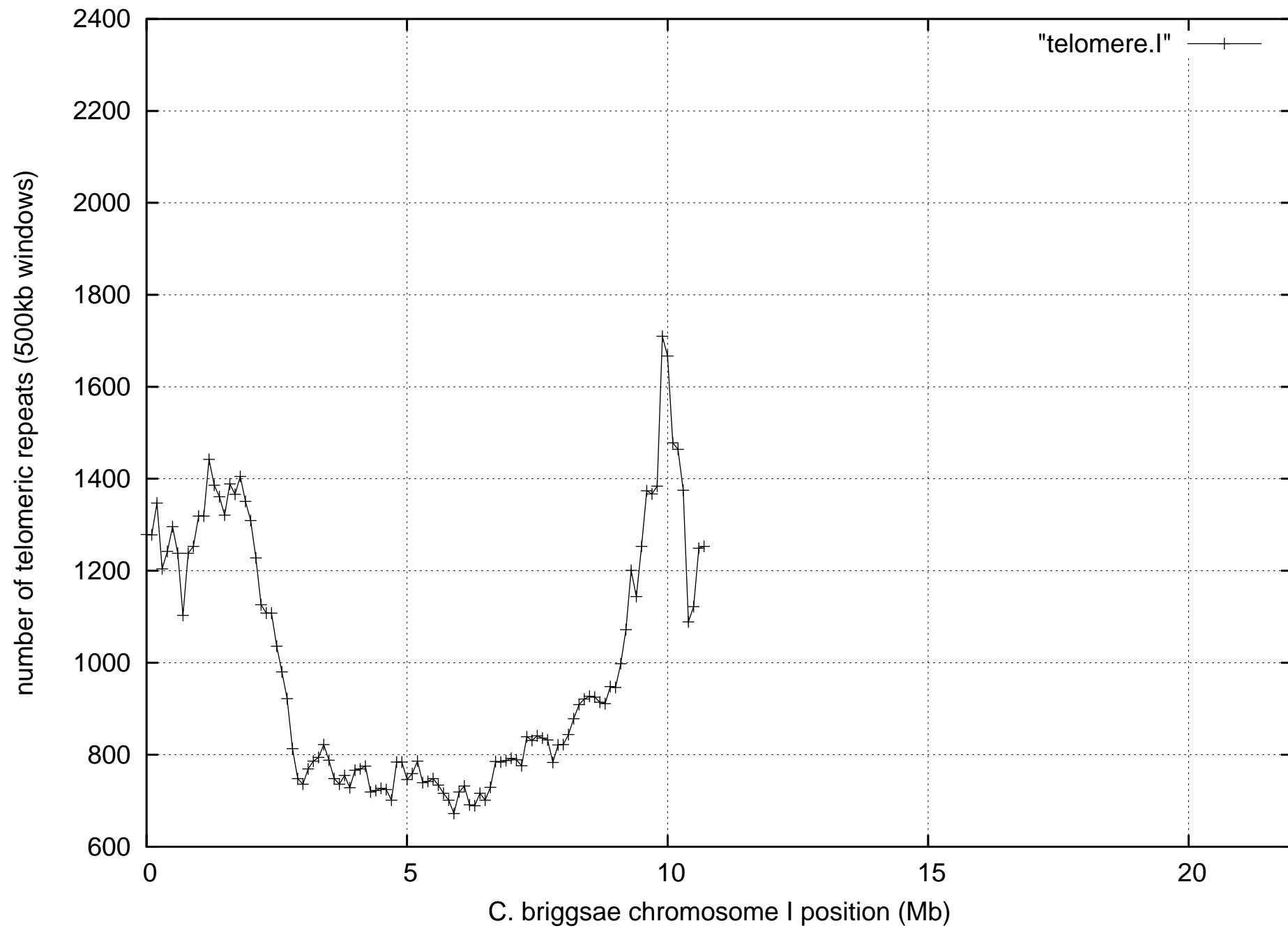

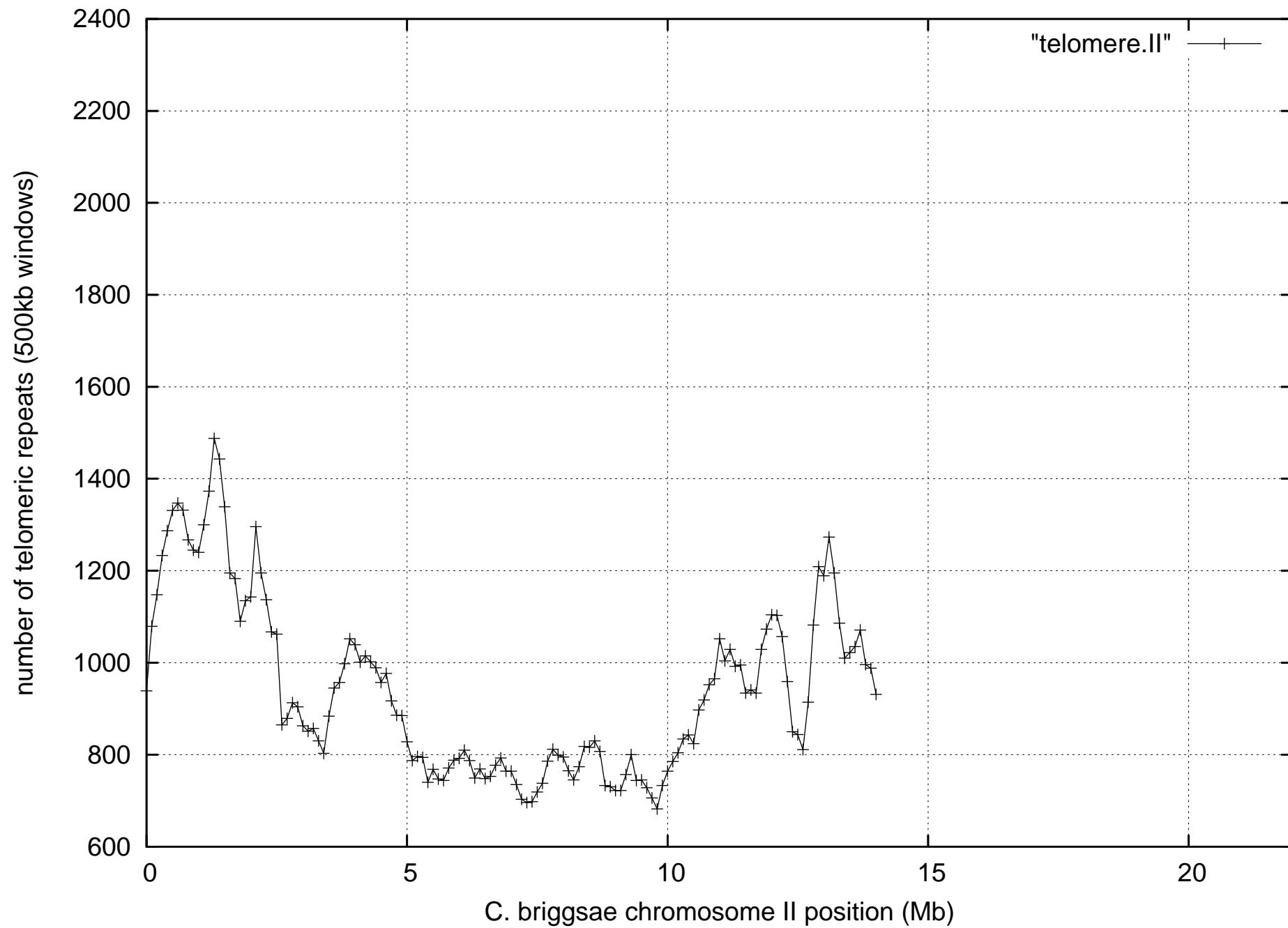

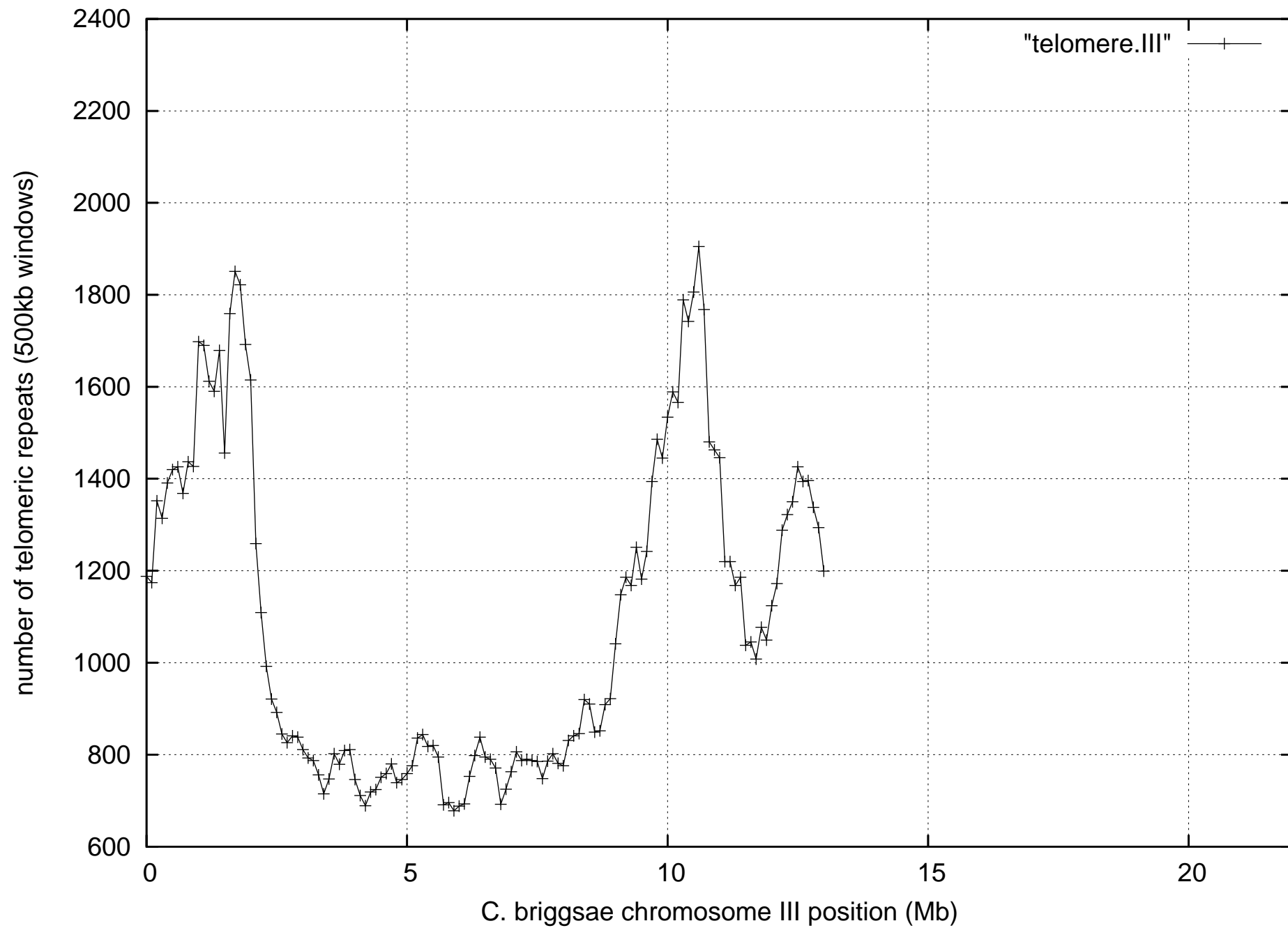

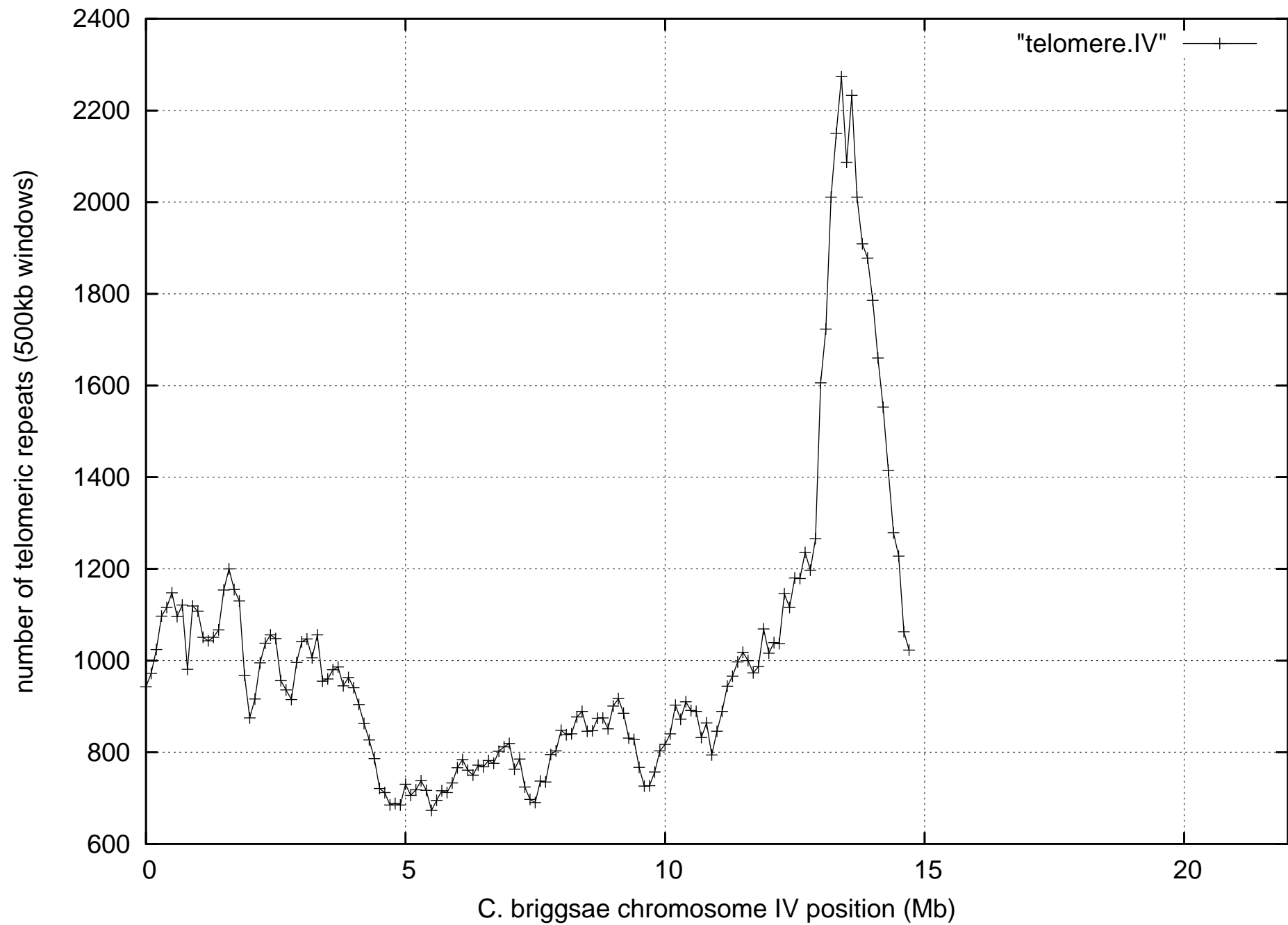

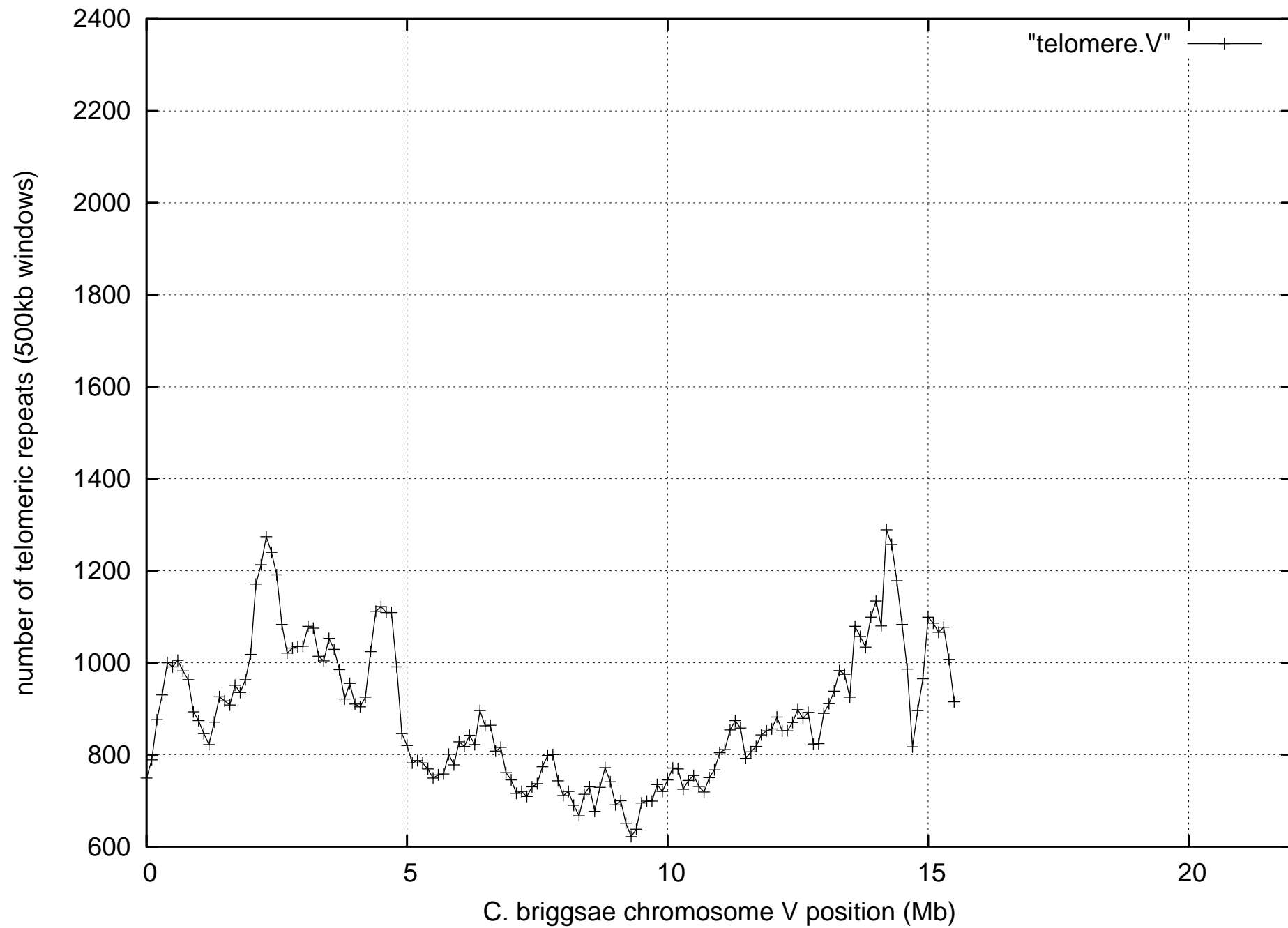

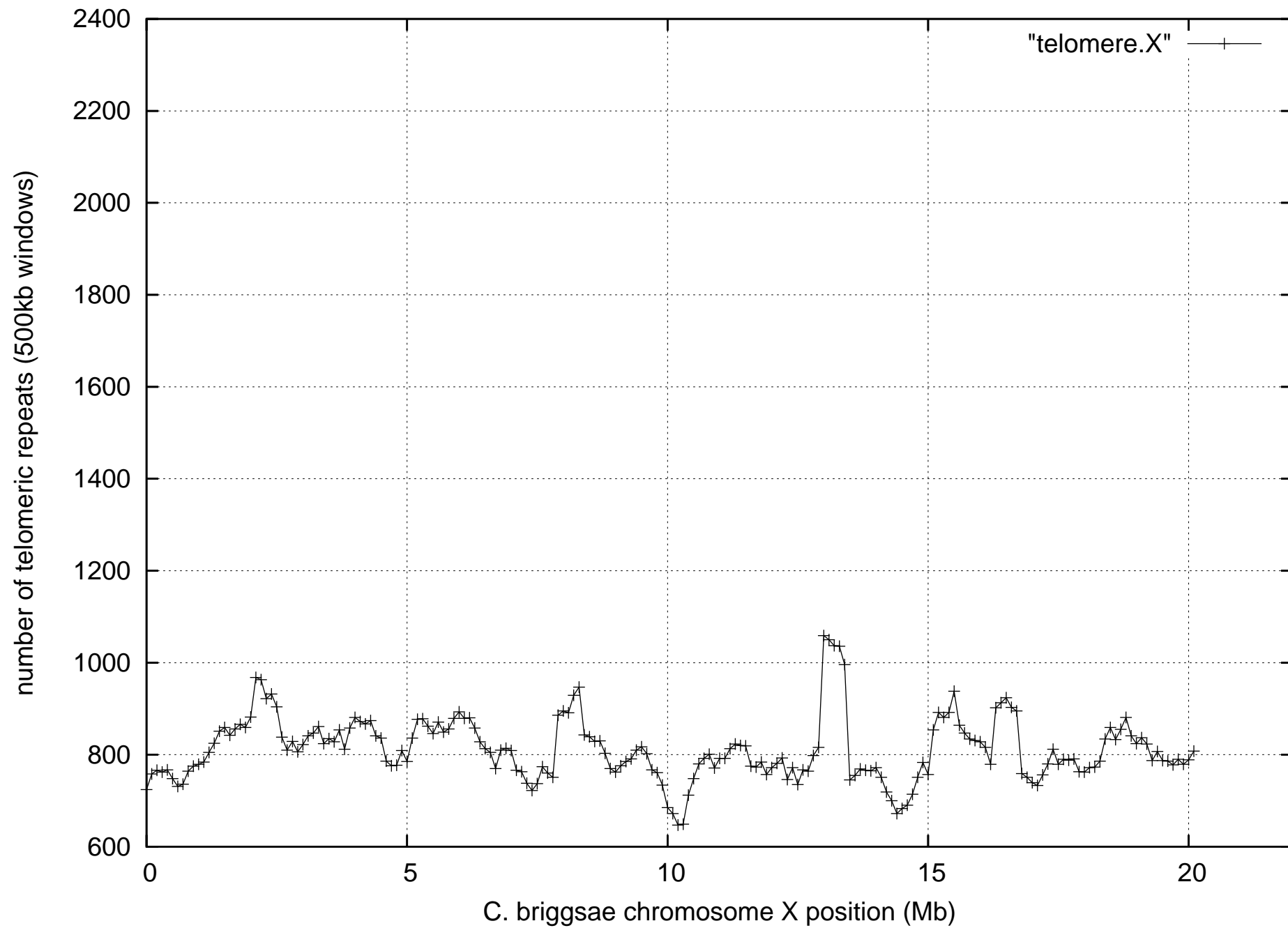

Supplement: Figure S1 — Graphs of each feature for each chromosome corresponding to the data provided in Dataset S3. (140 KB PDF) [file pbio.0050167.sg001.pdf]

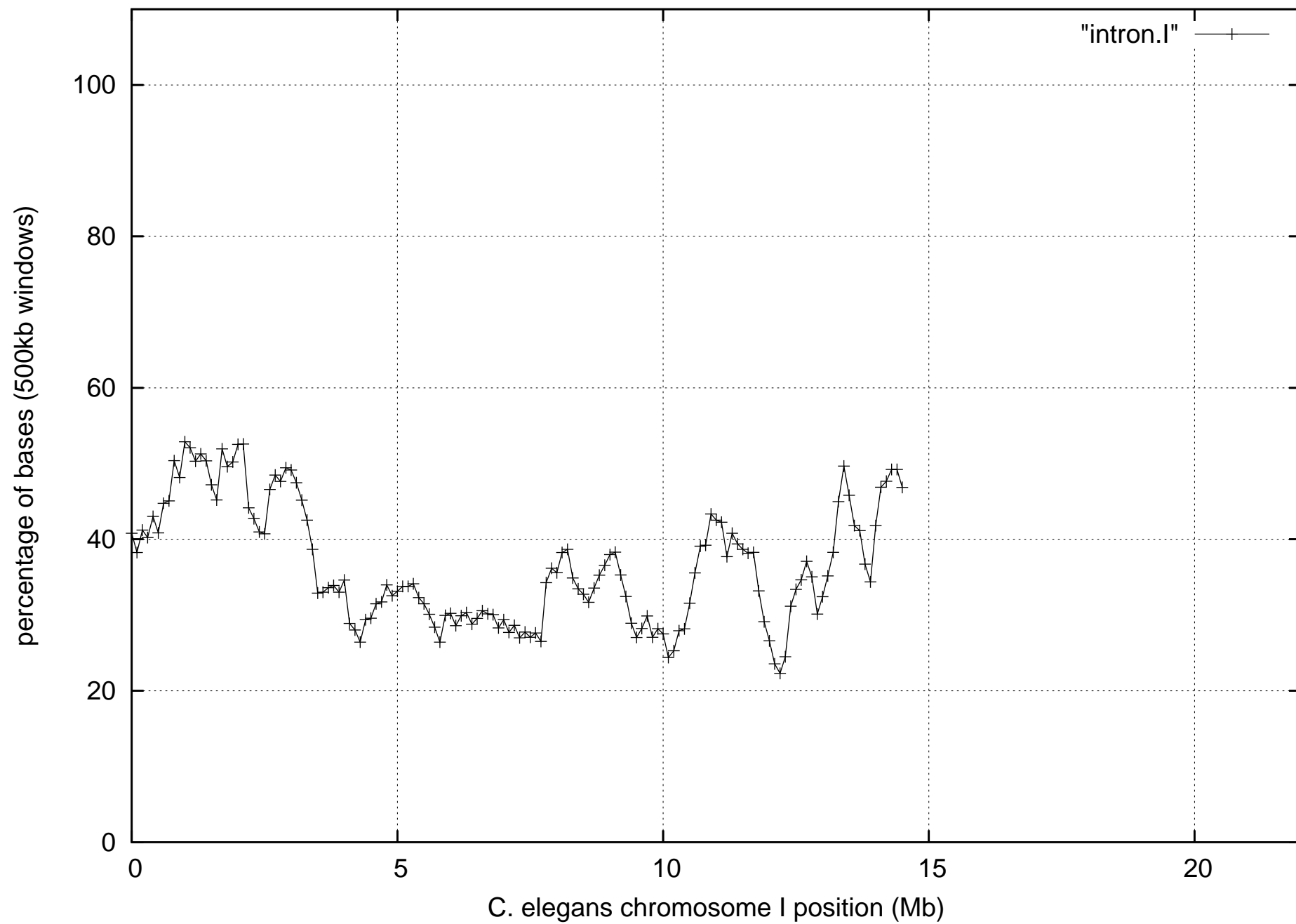

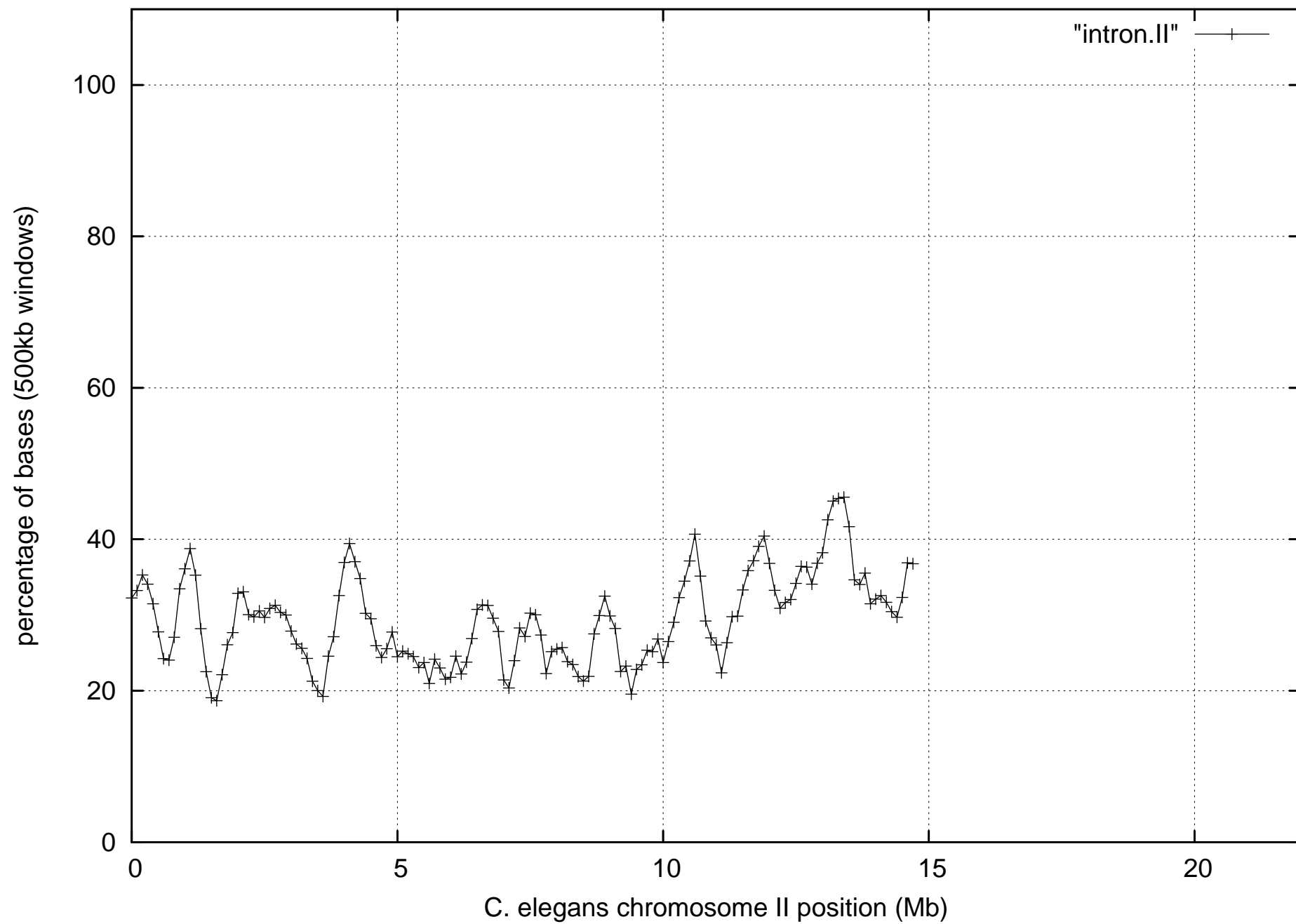

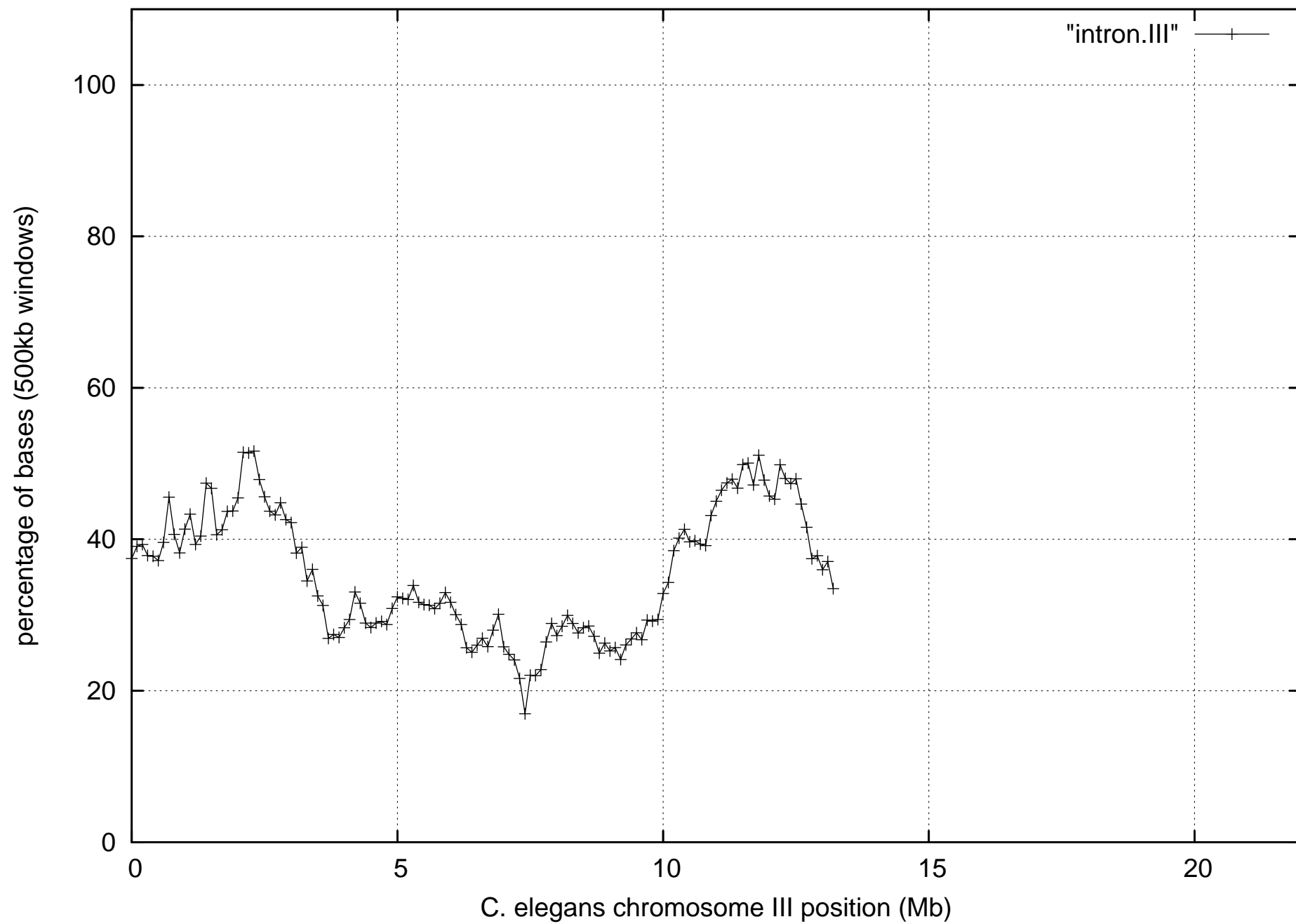

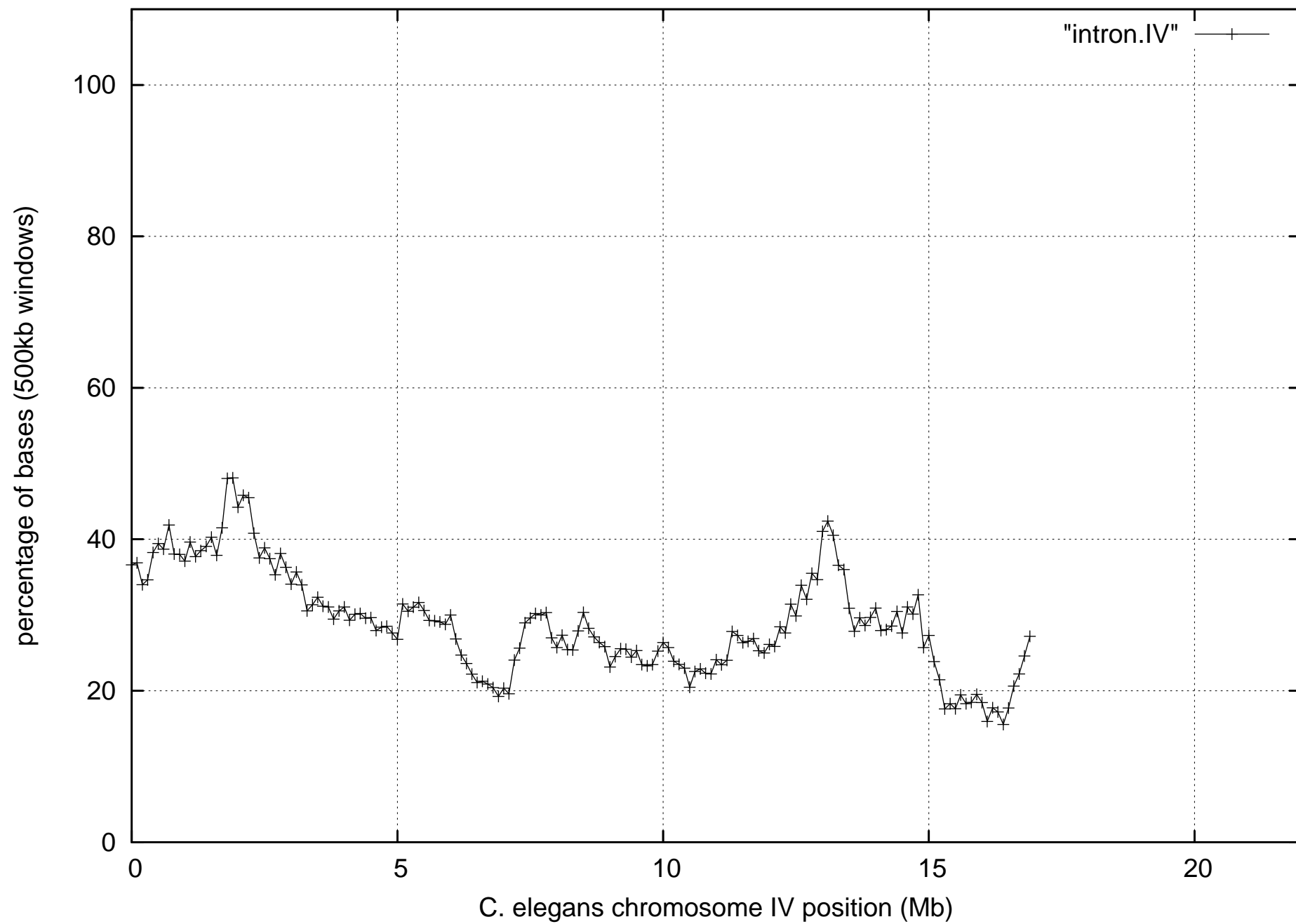

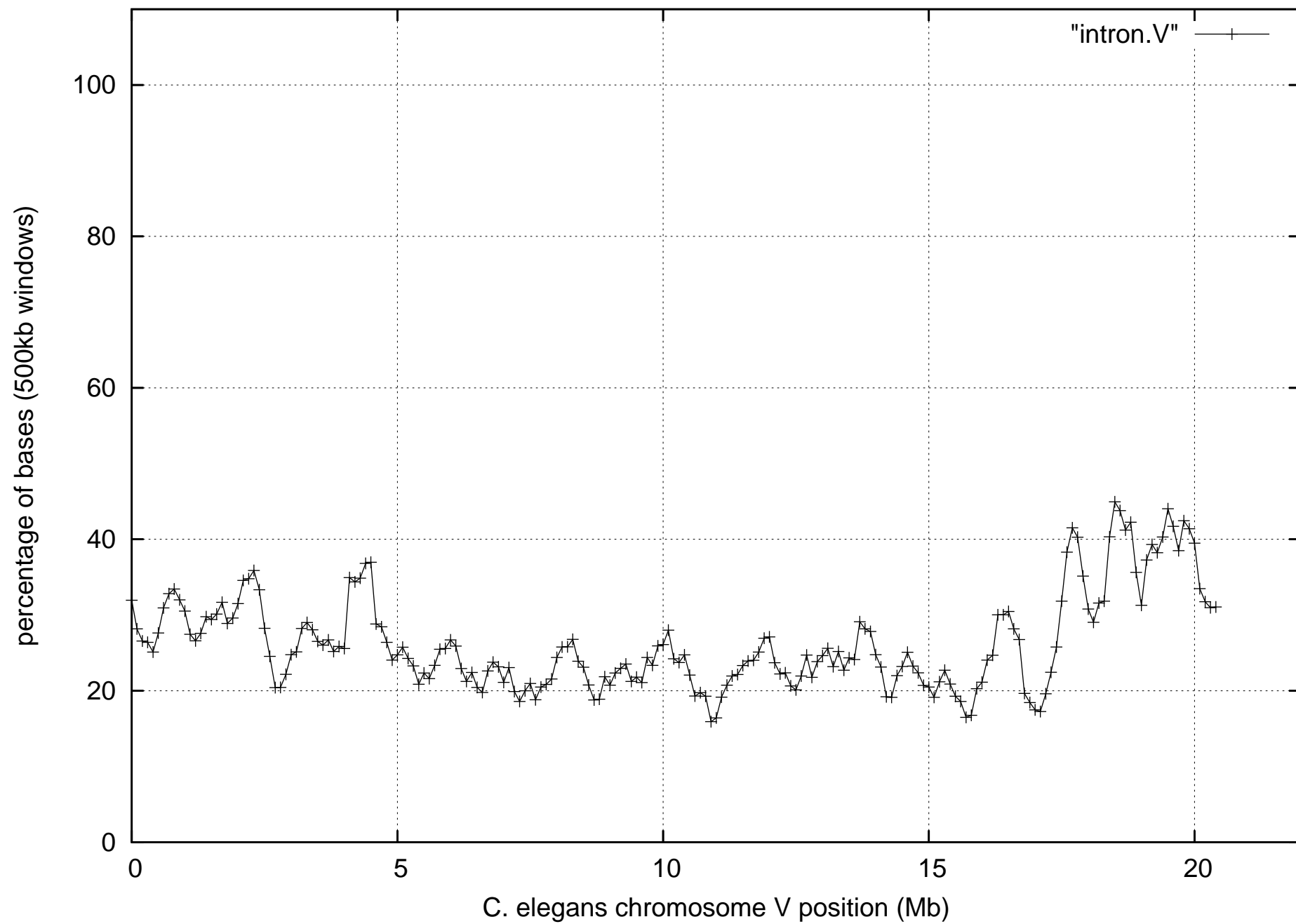

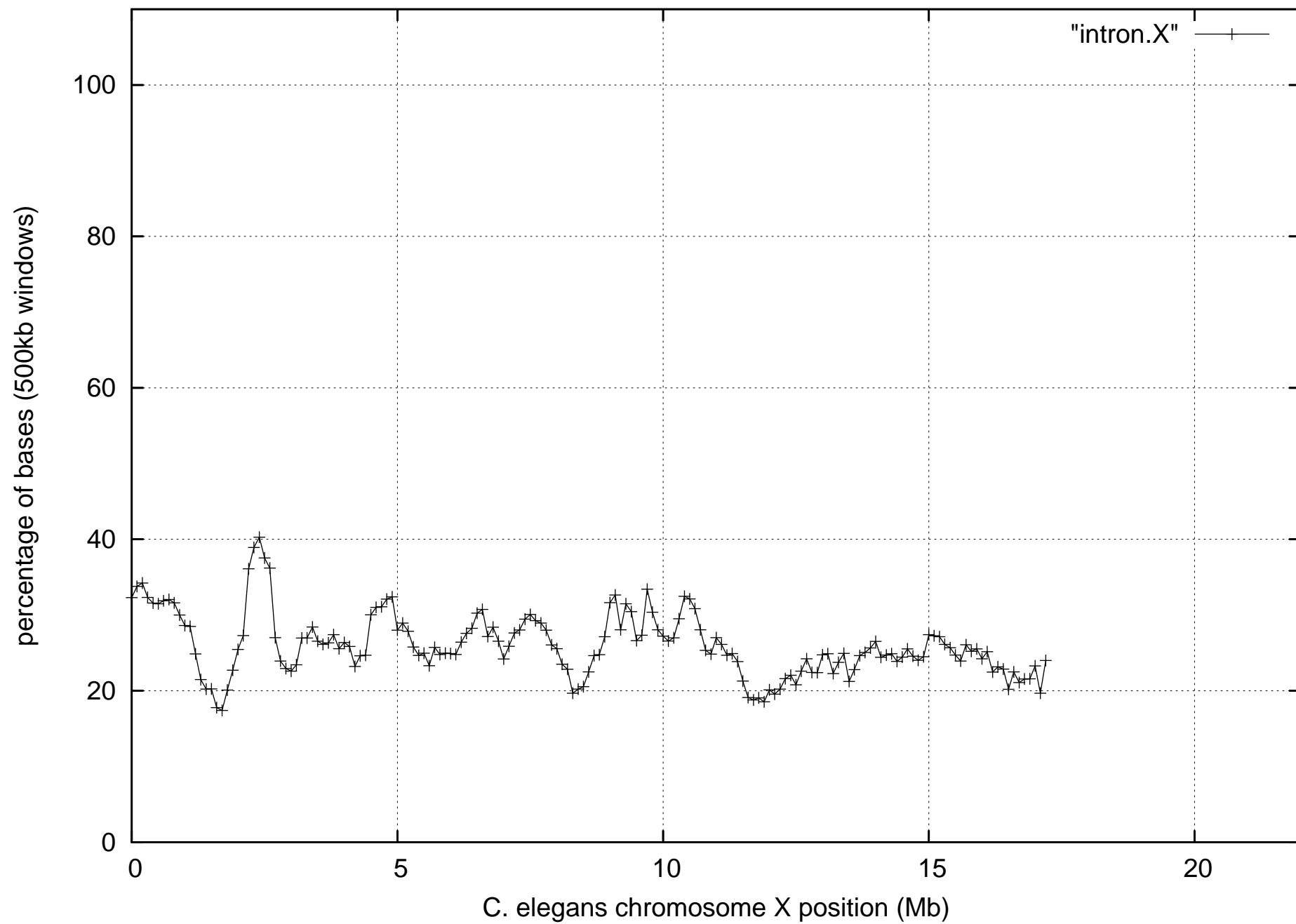

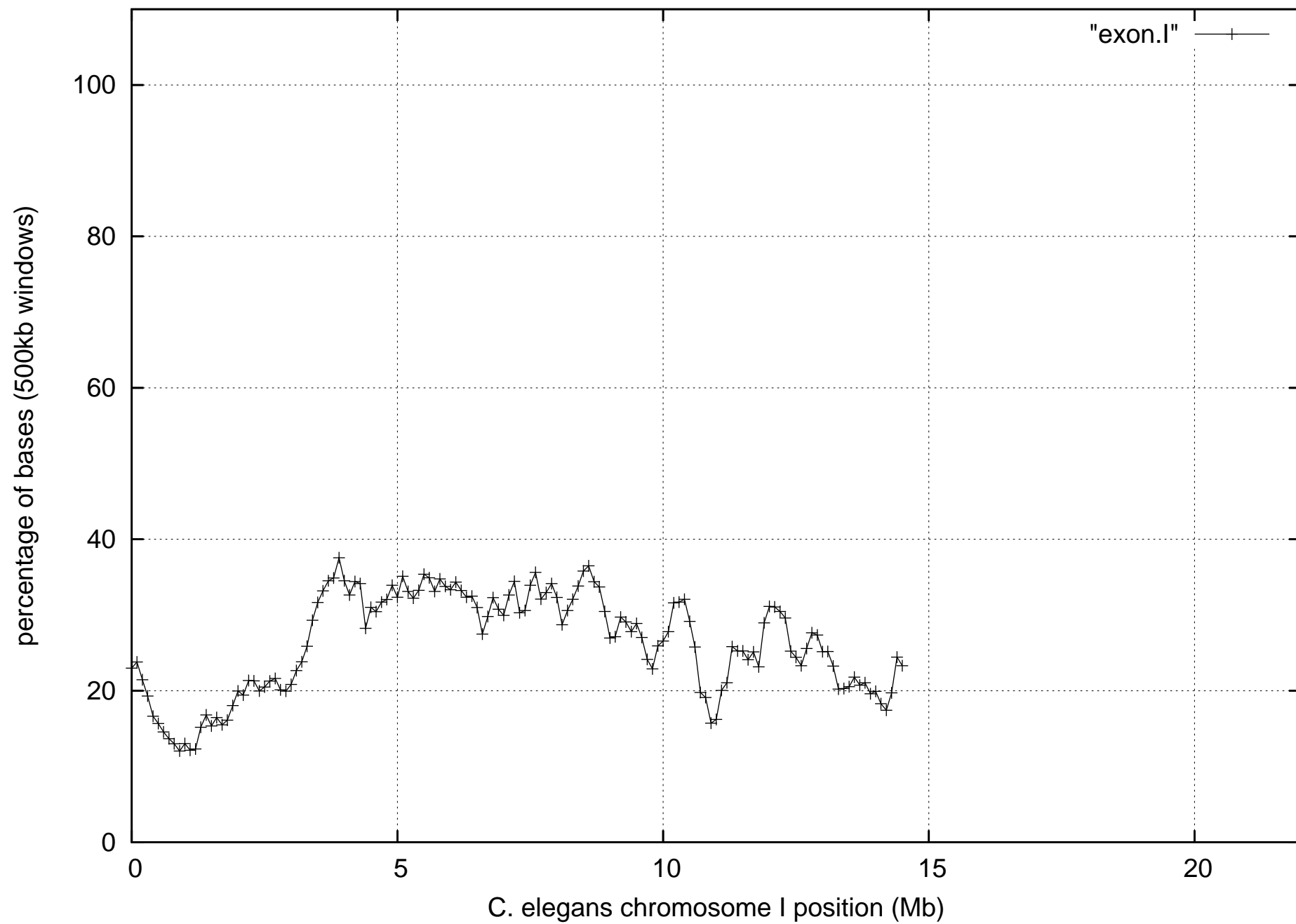

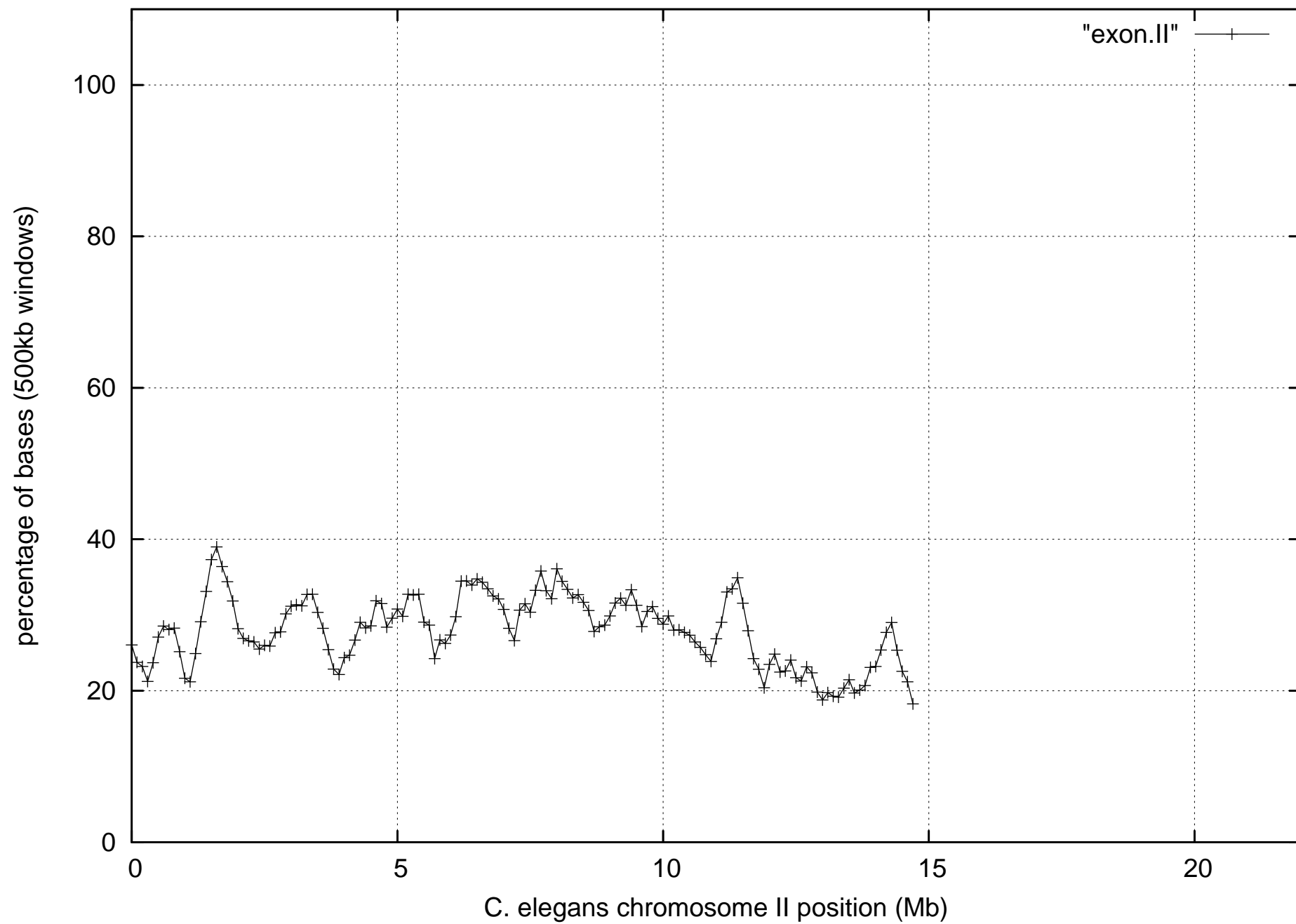

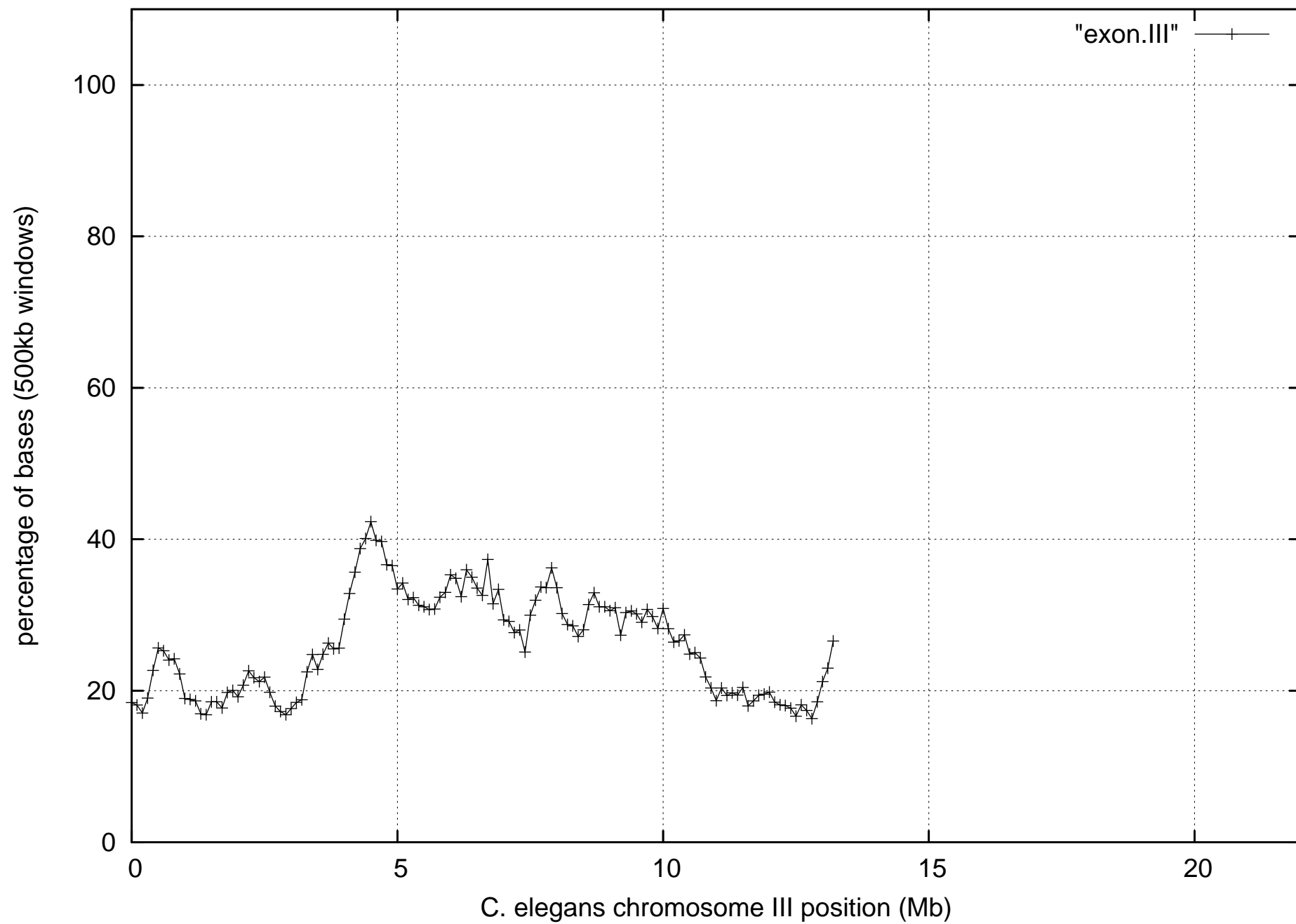

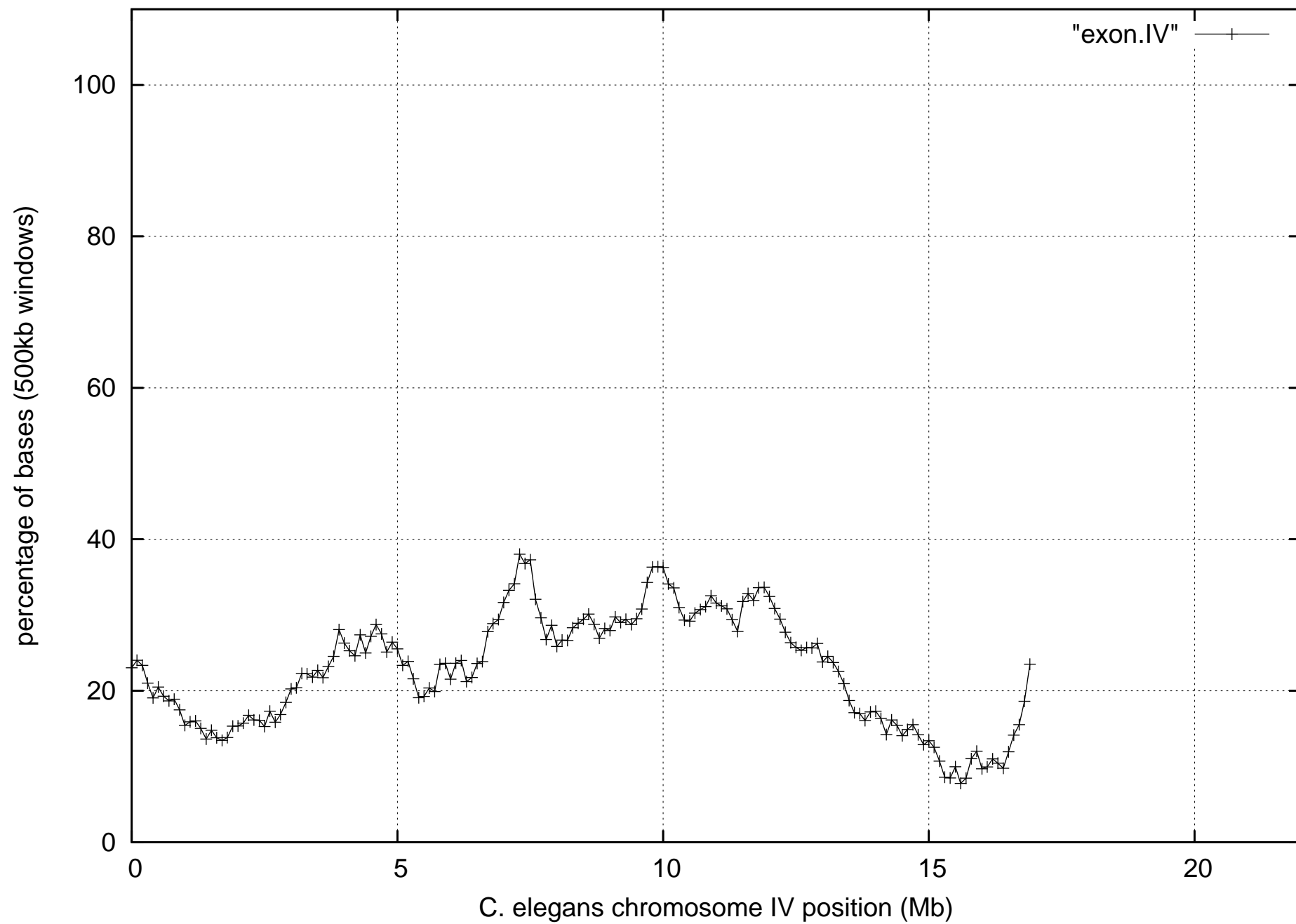

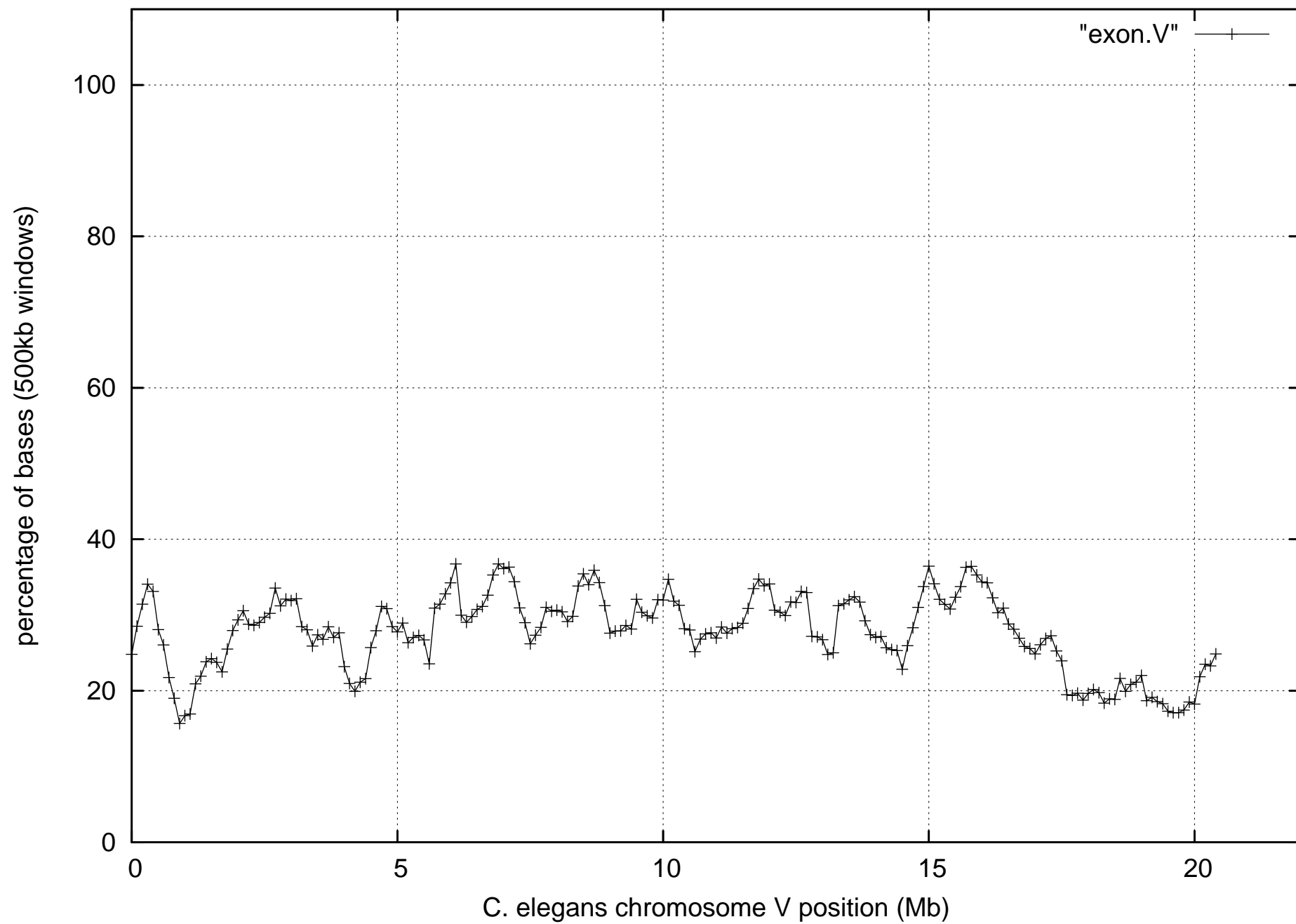

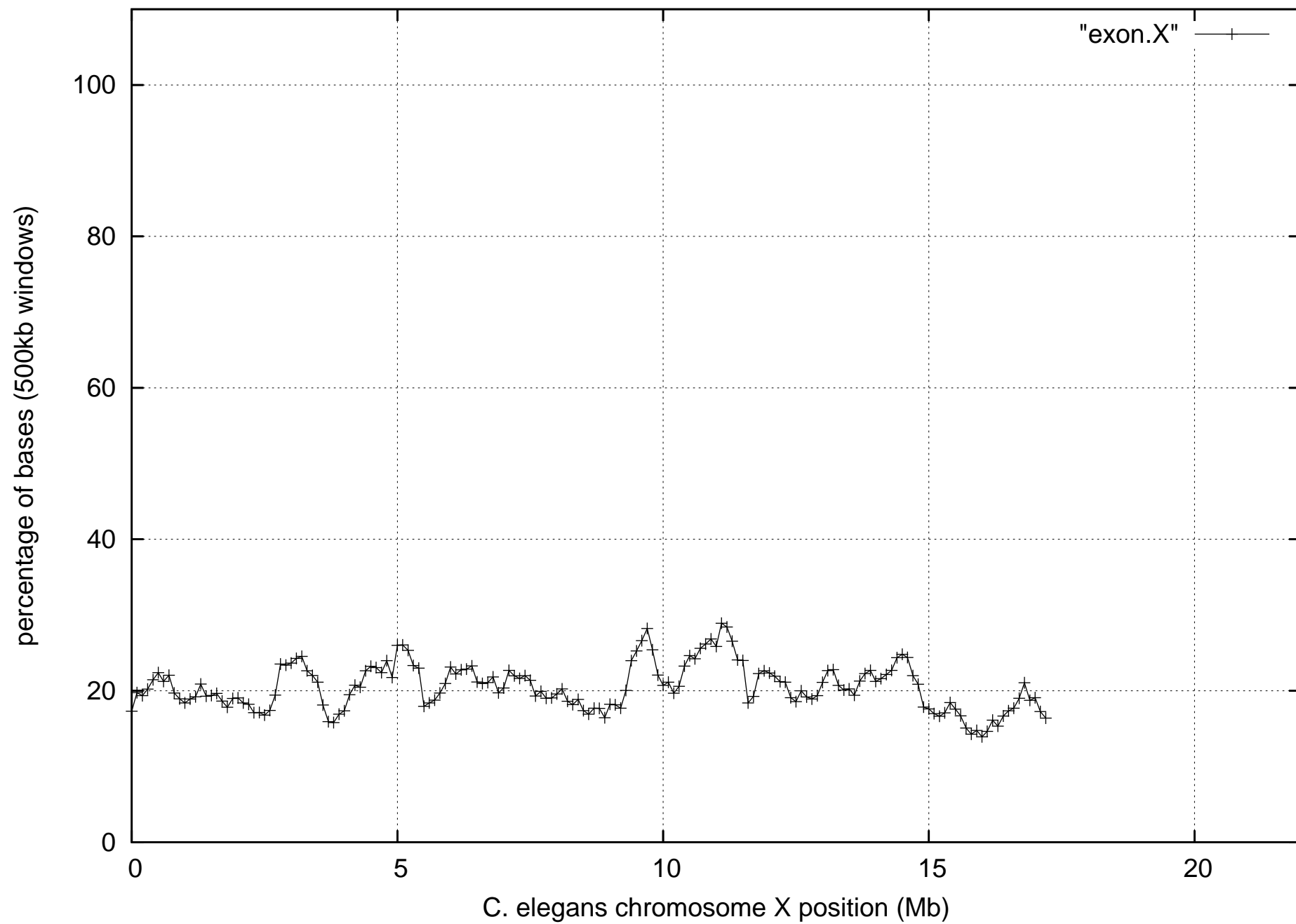

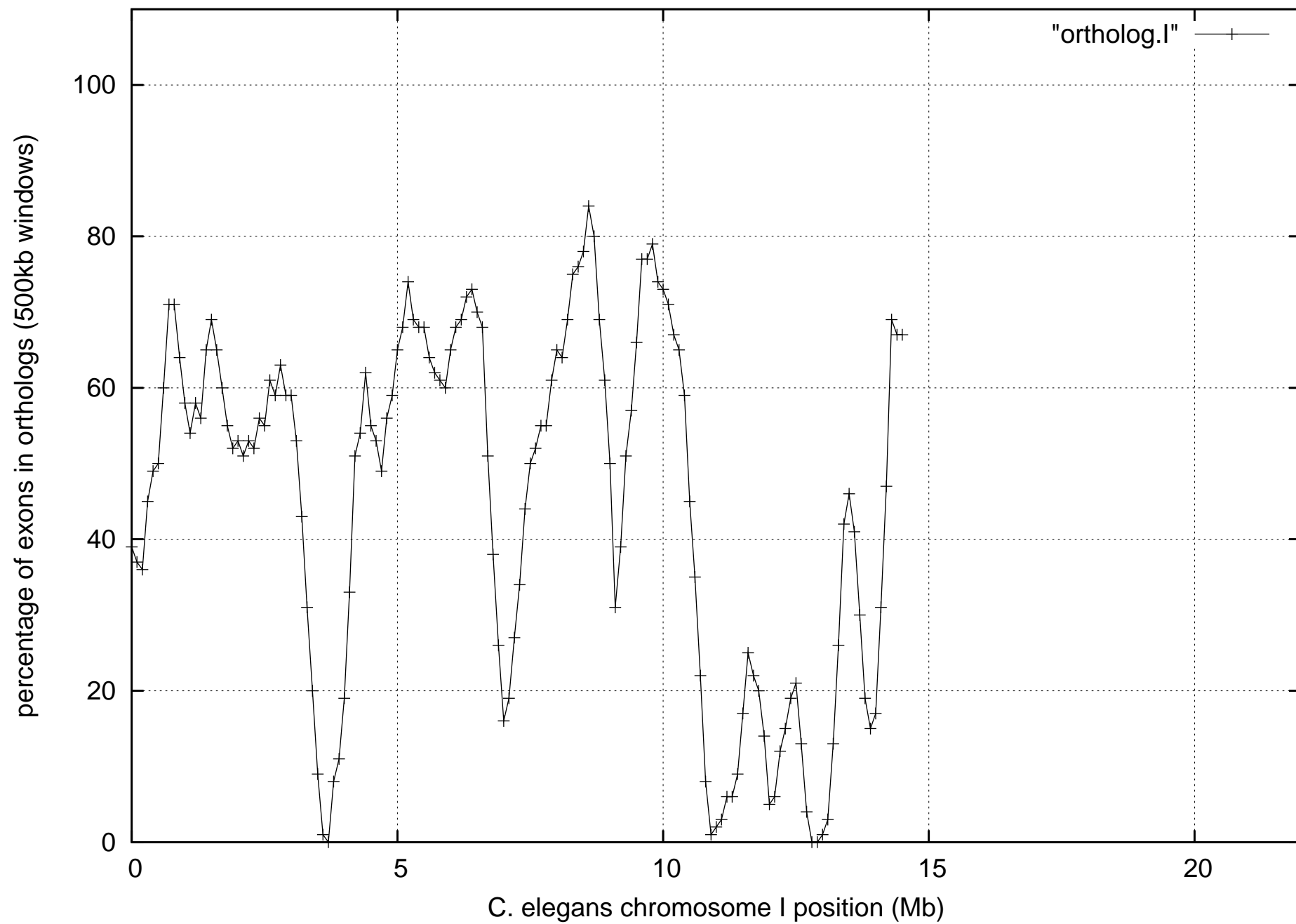

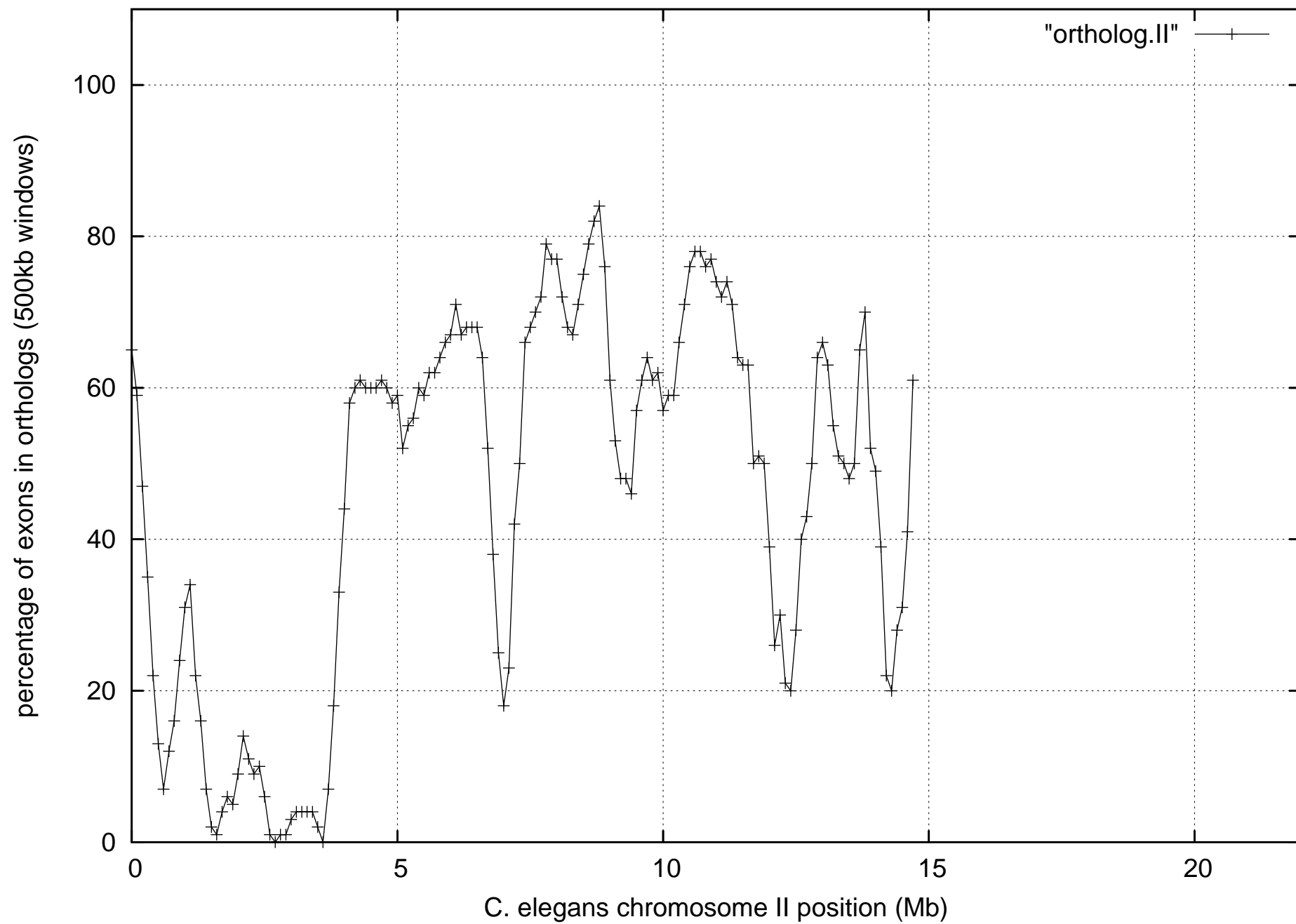

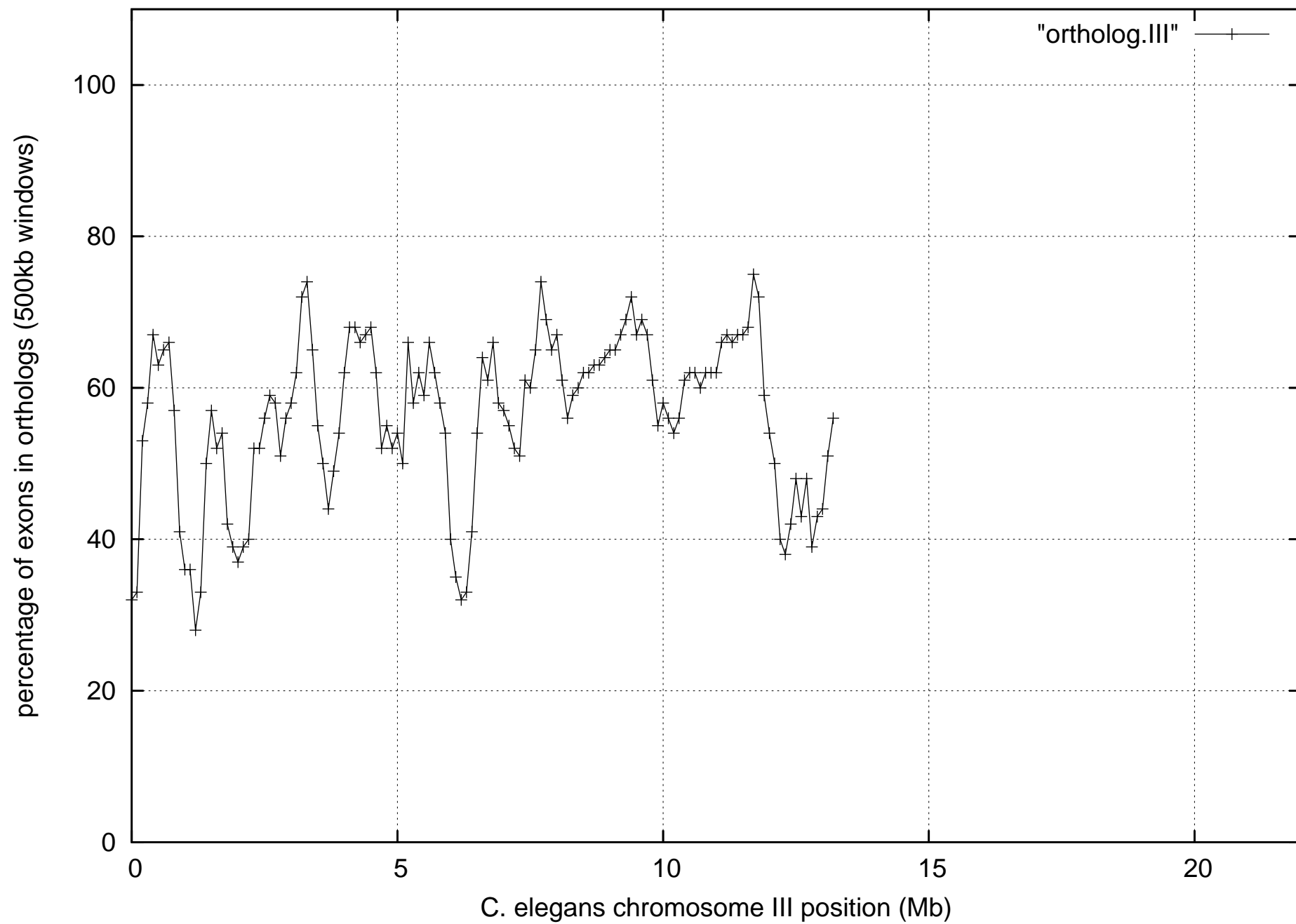

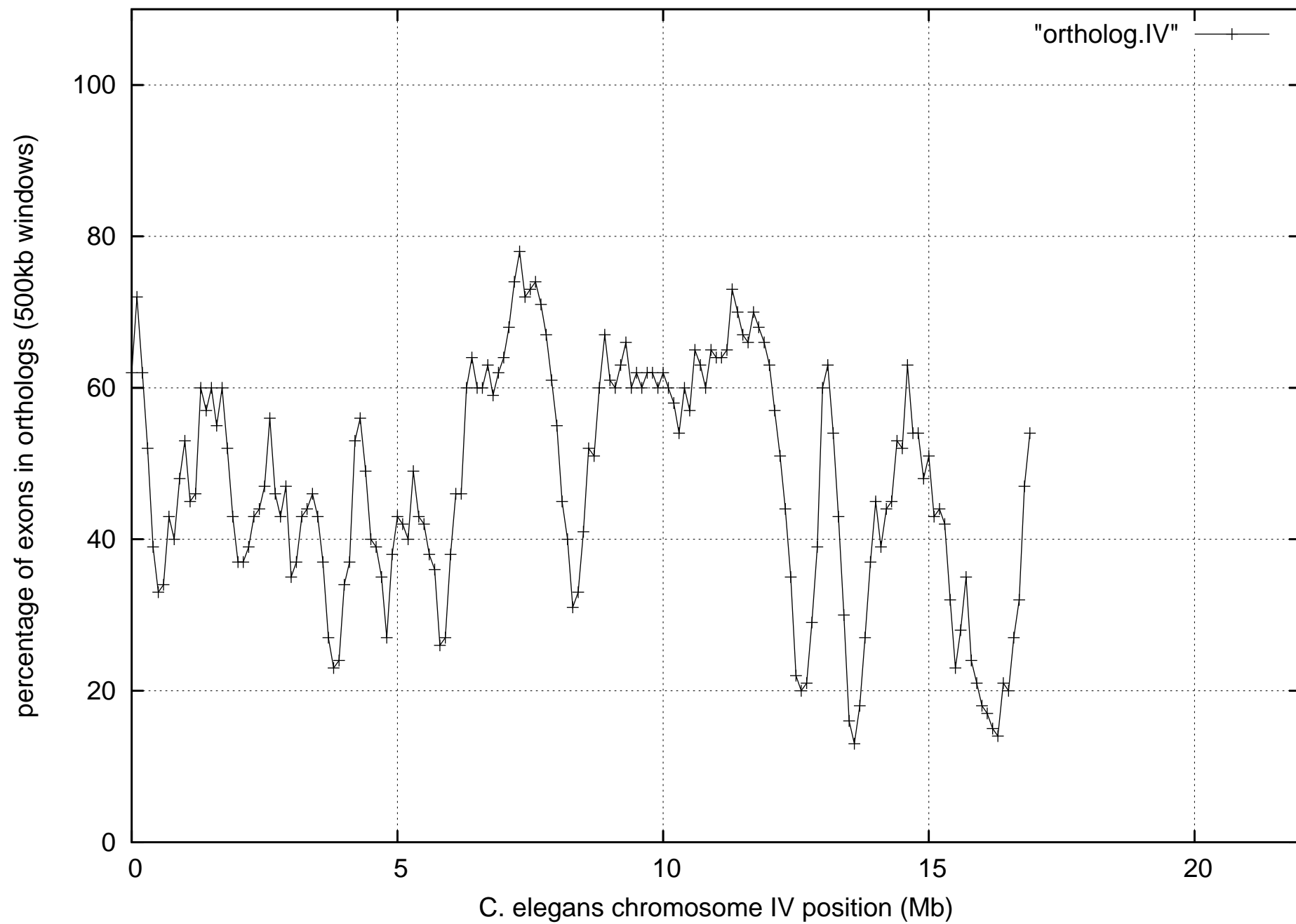

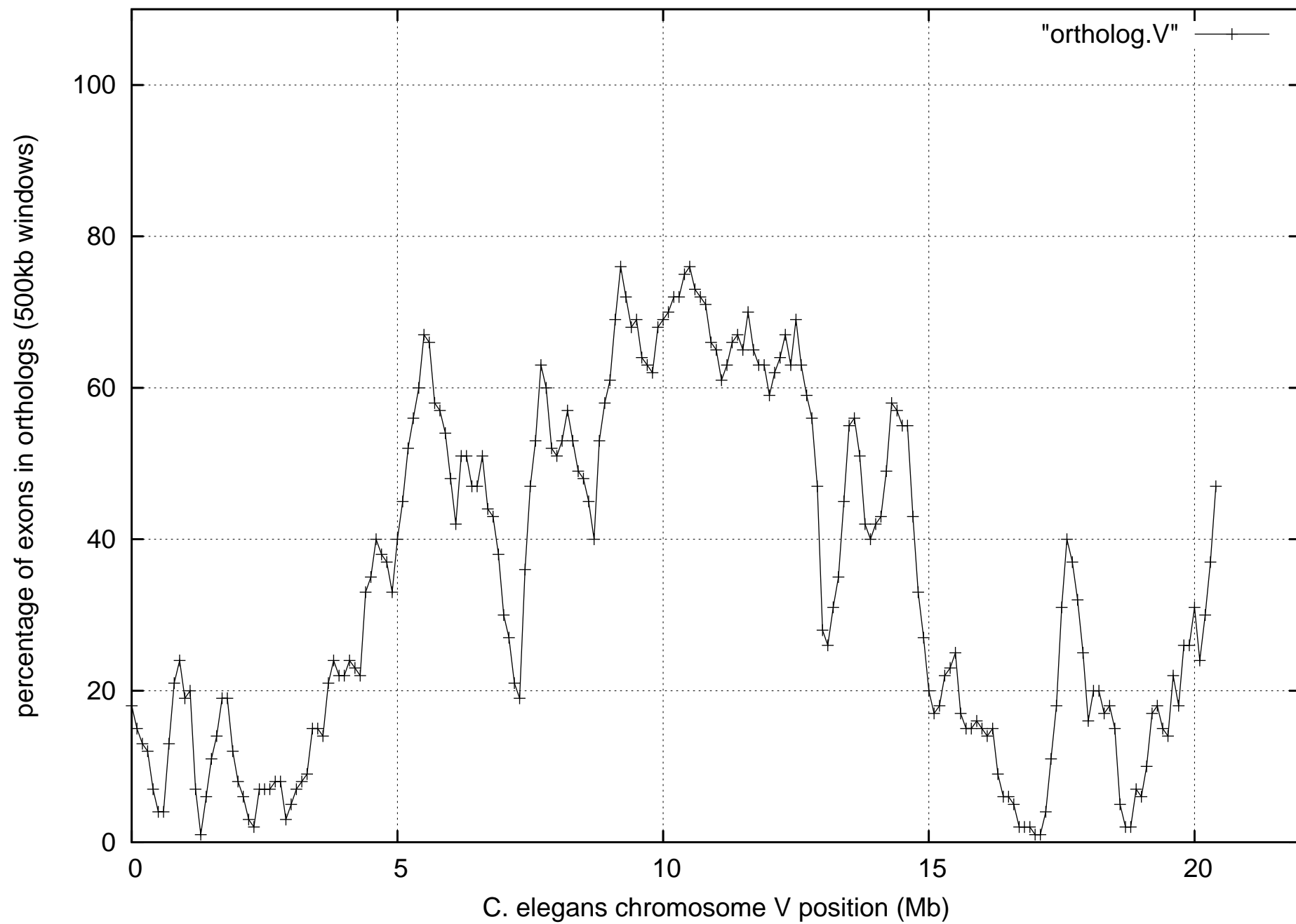

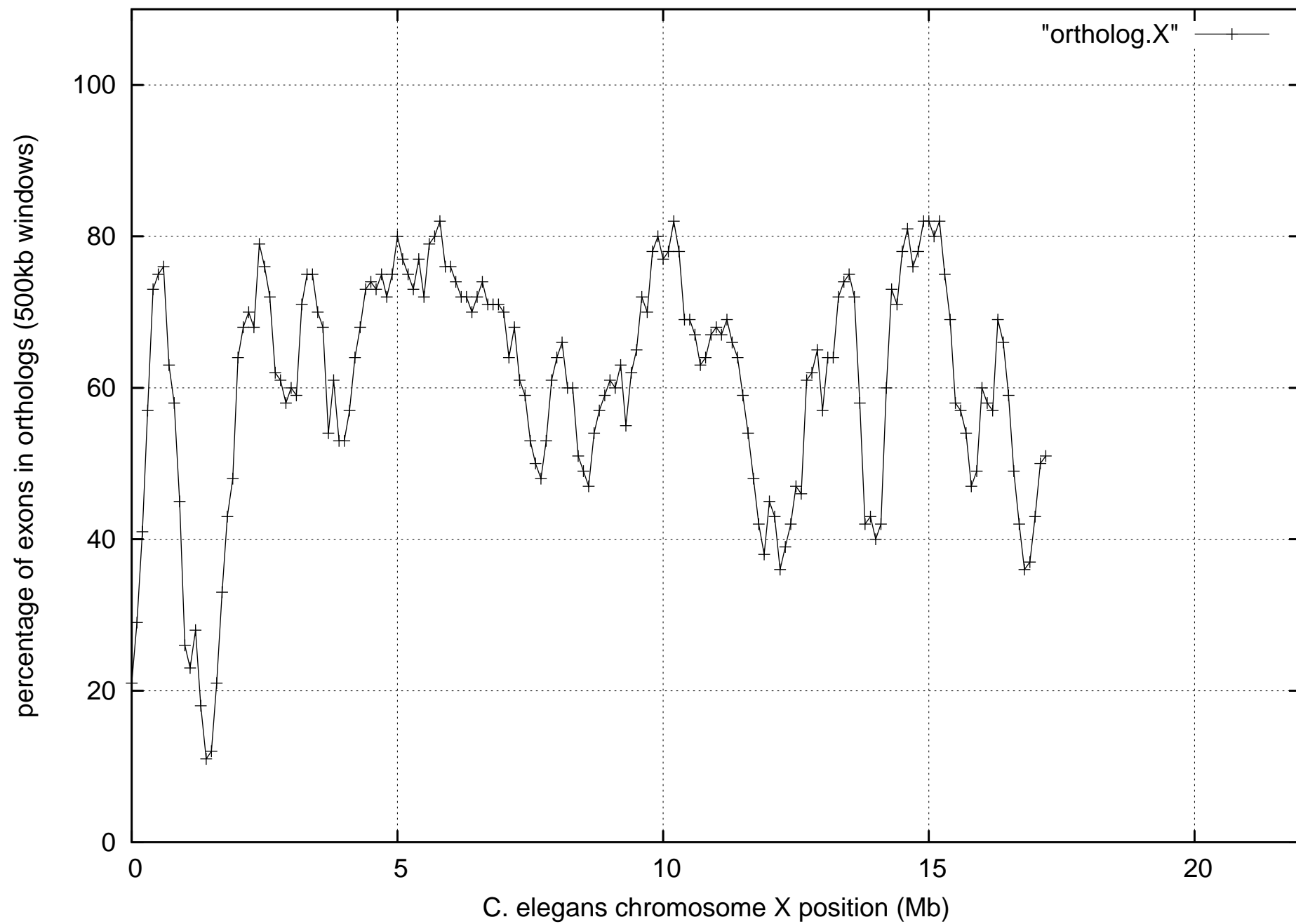

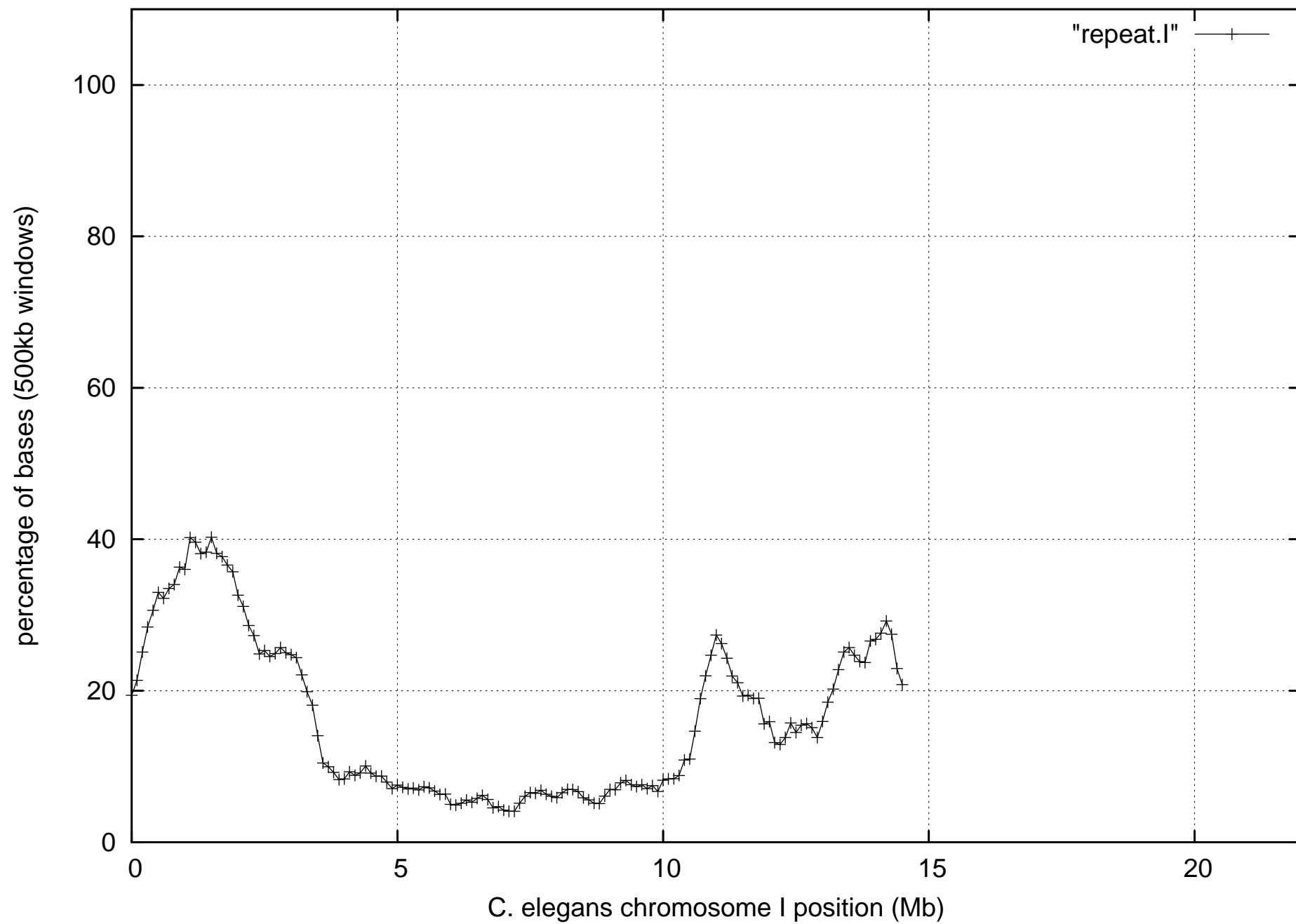

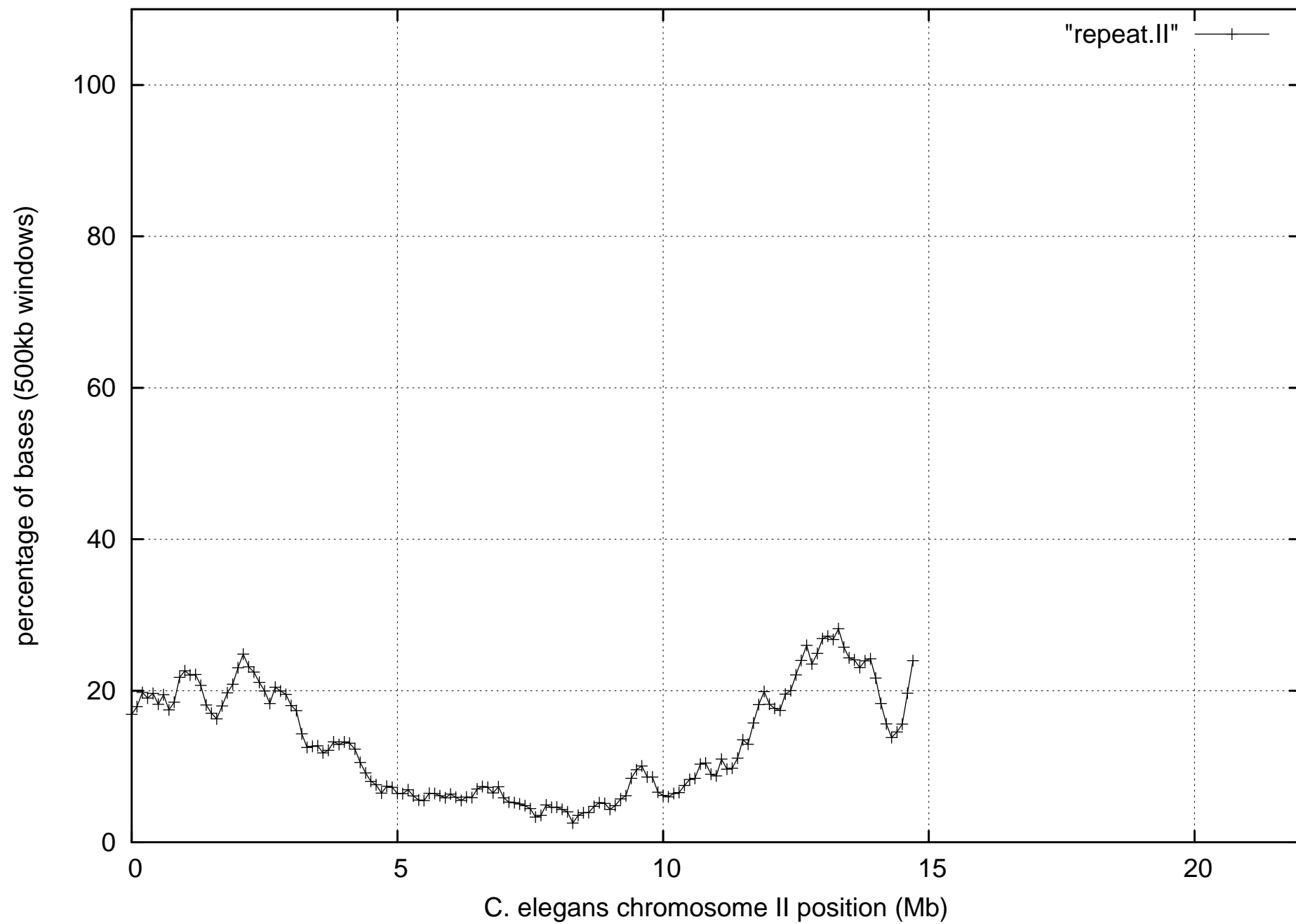

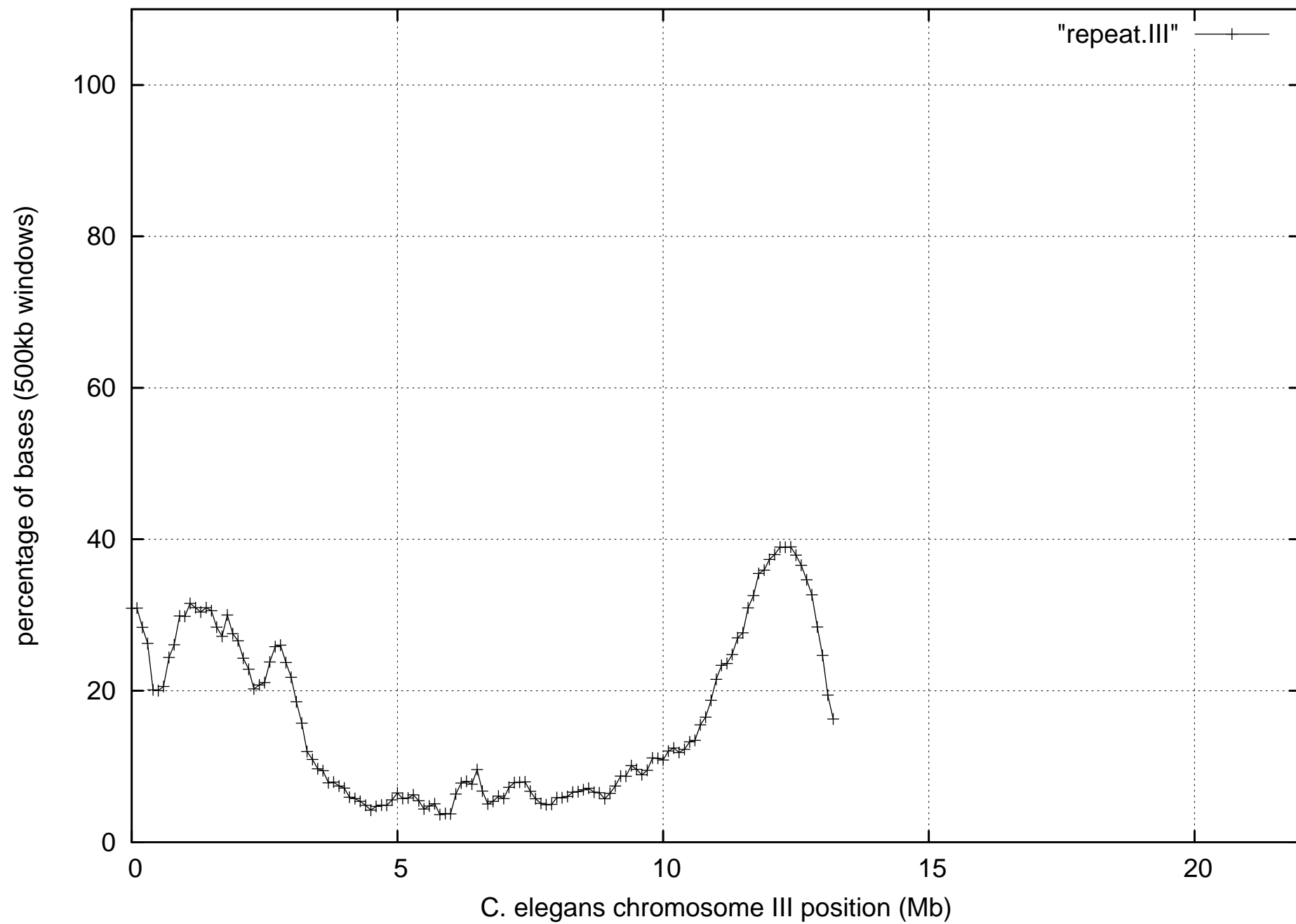

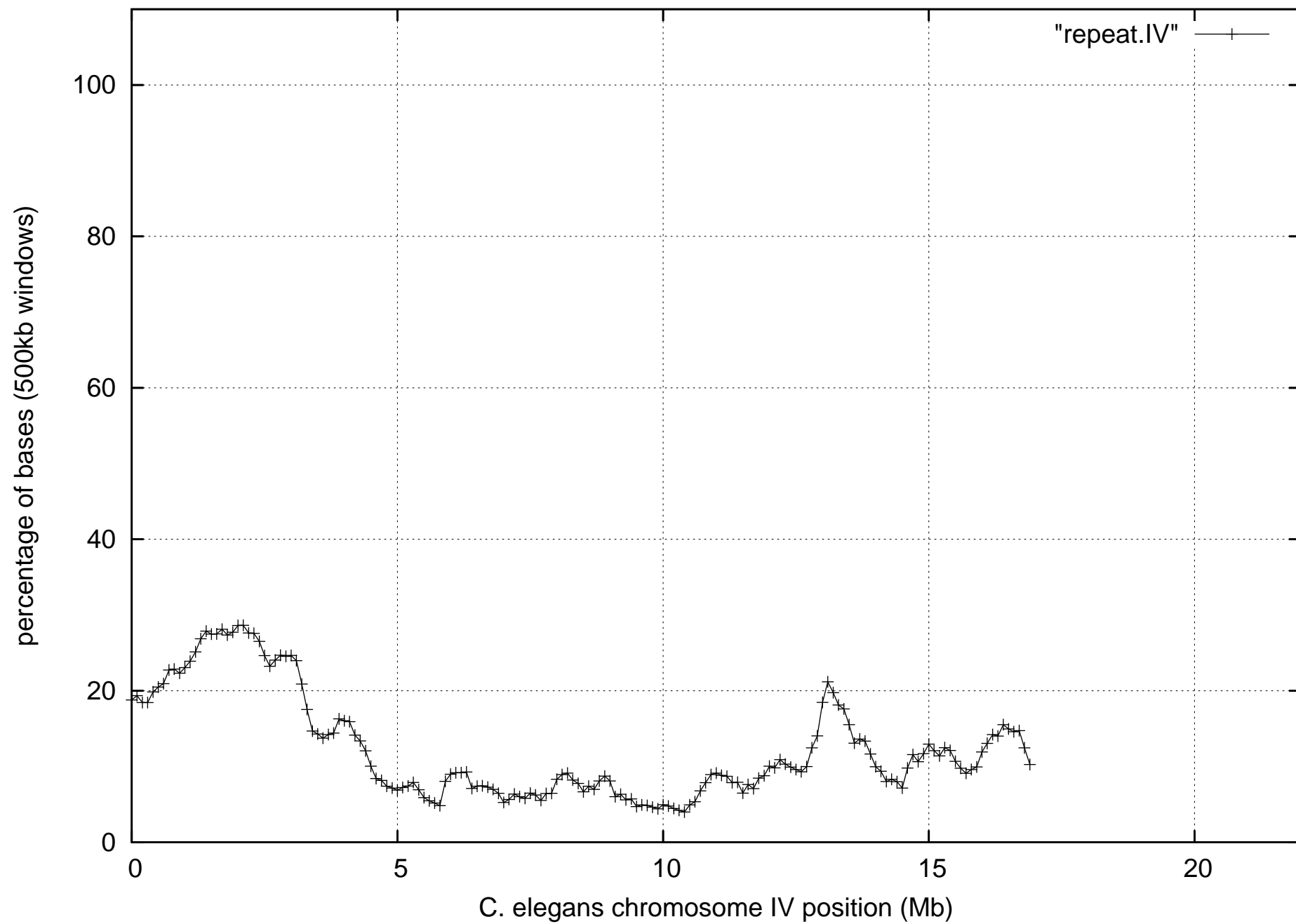

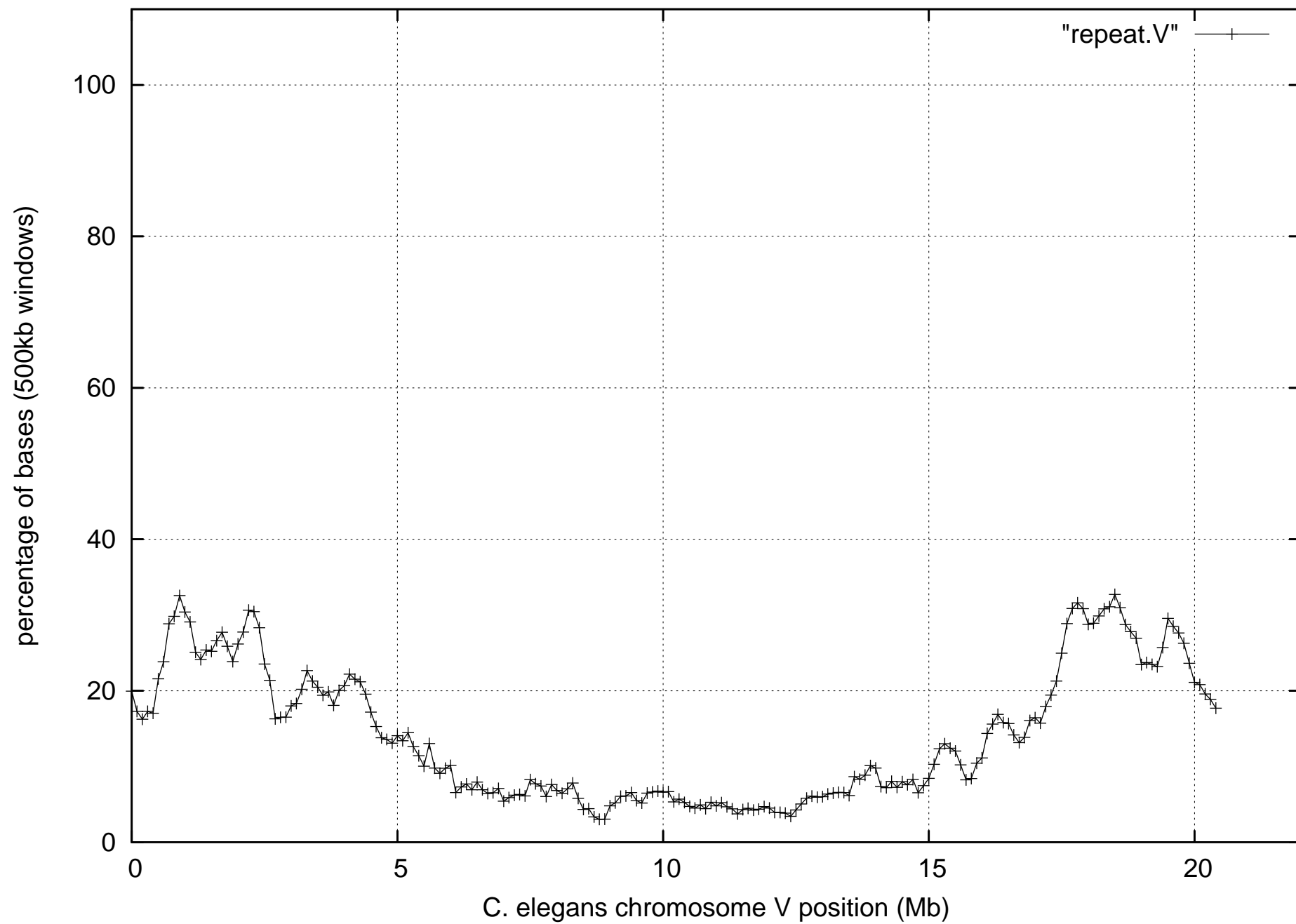

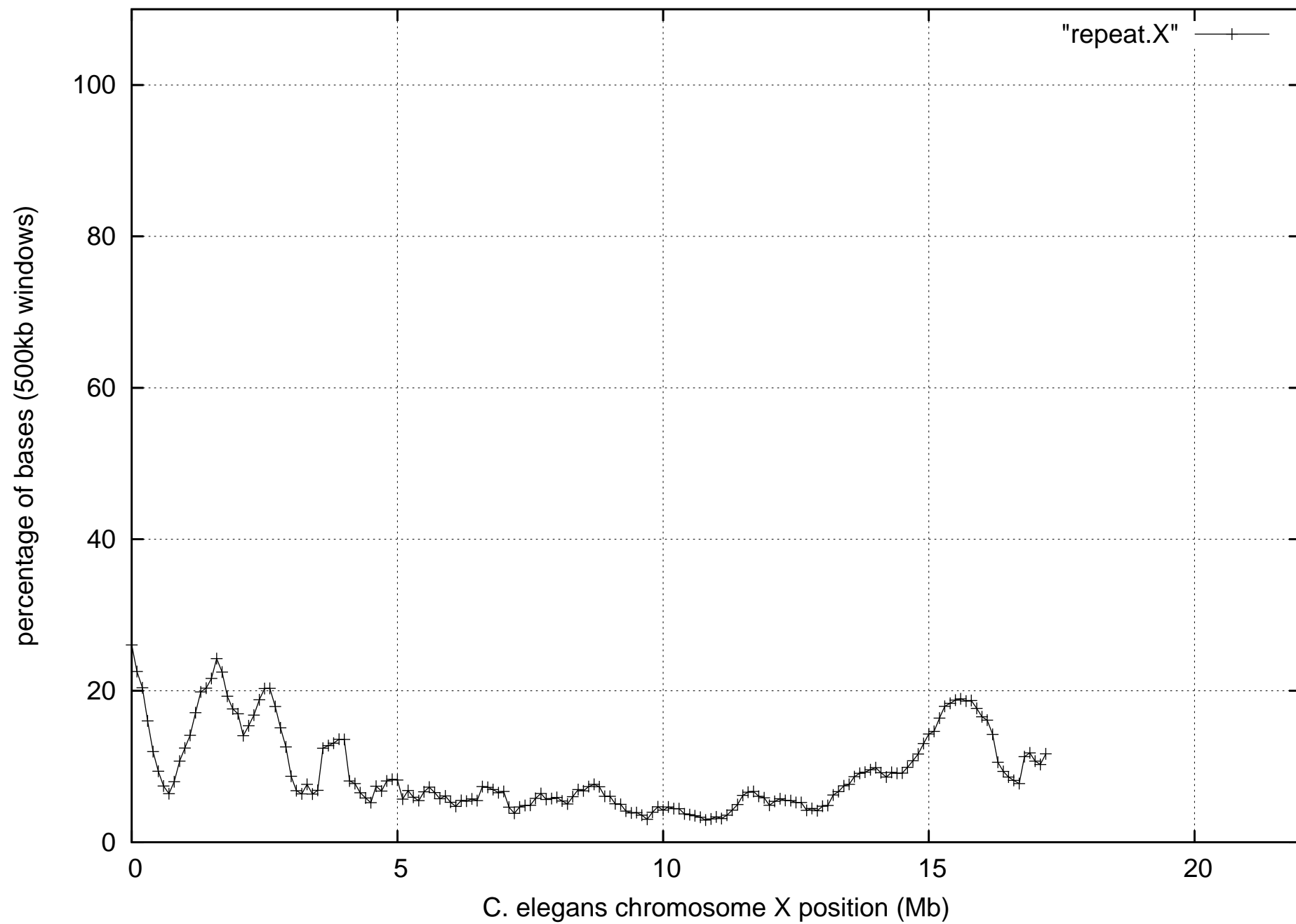

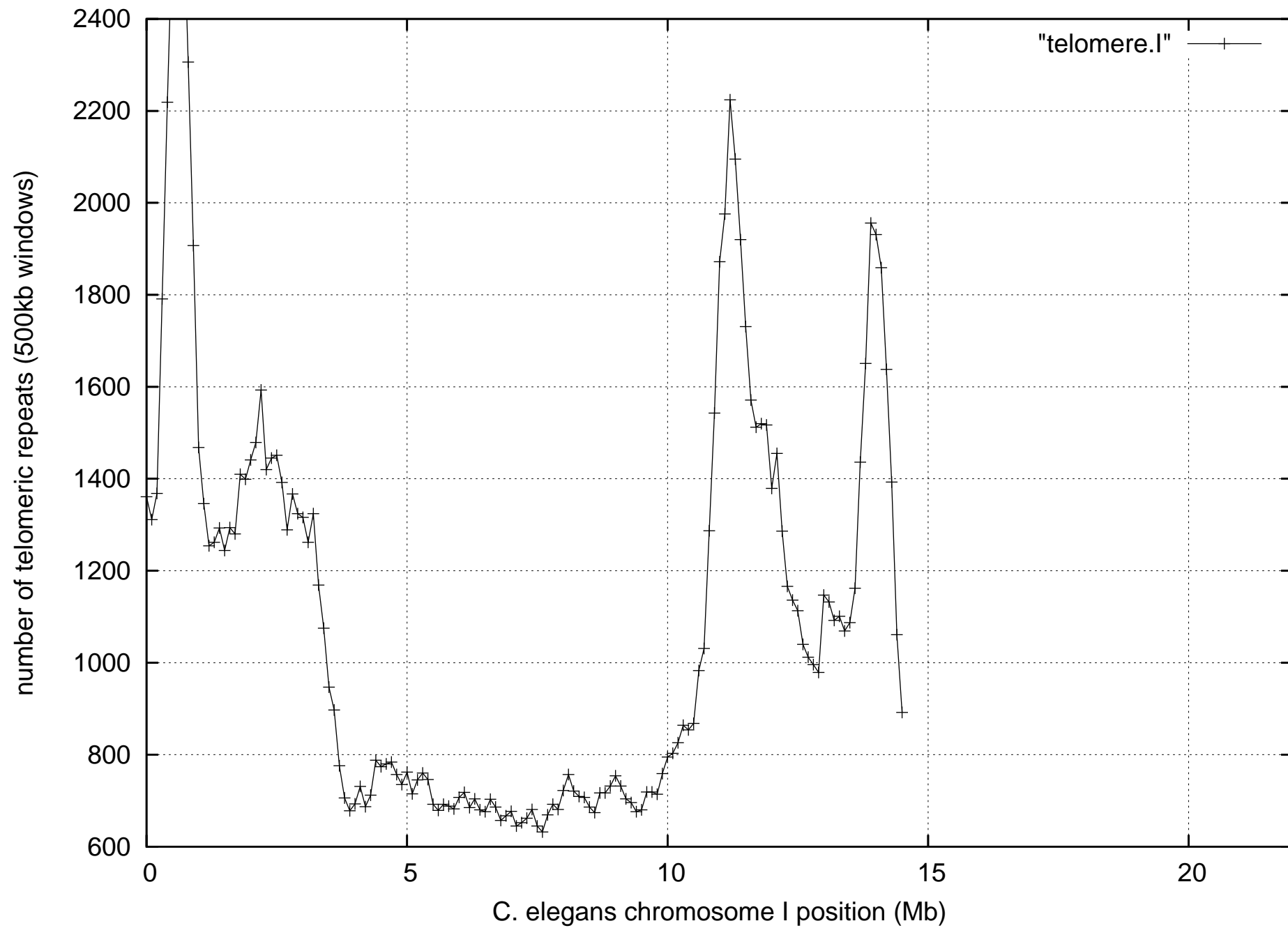

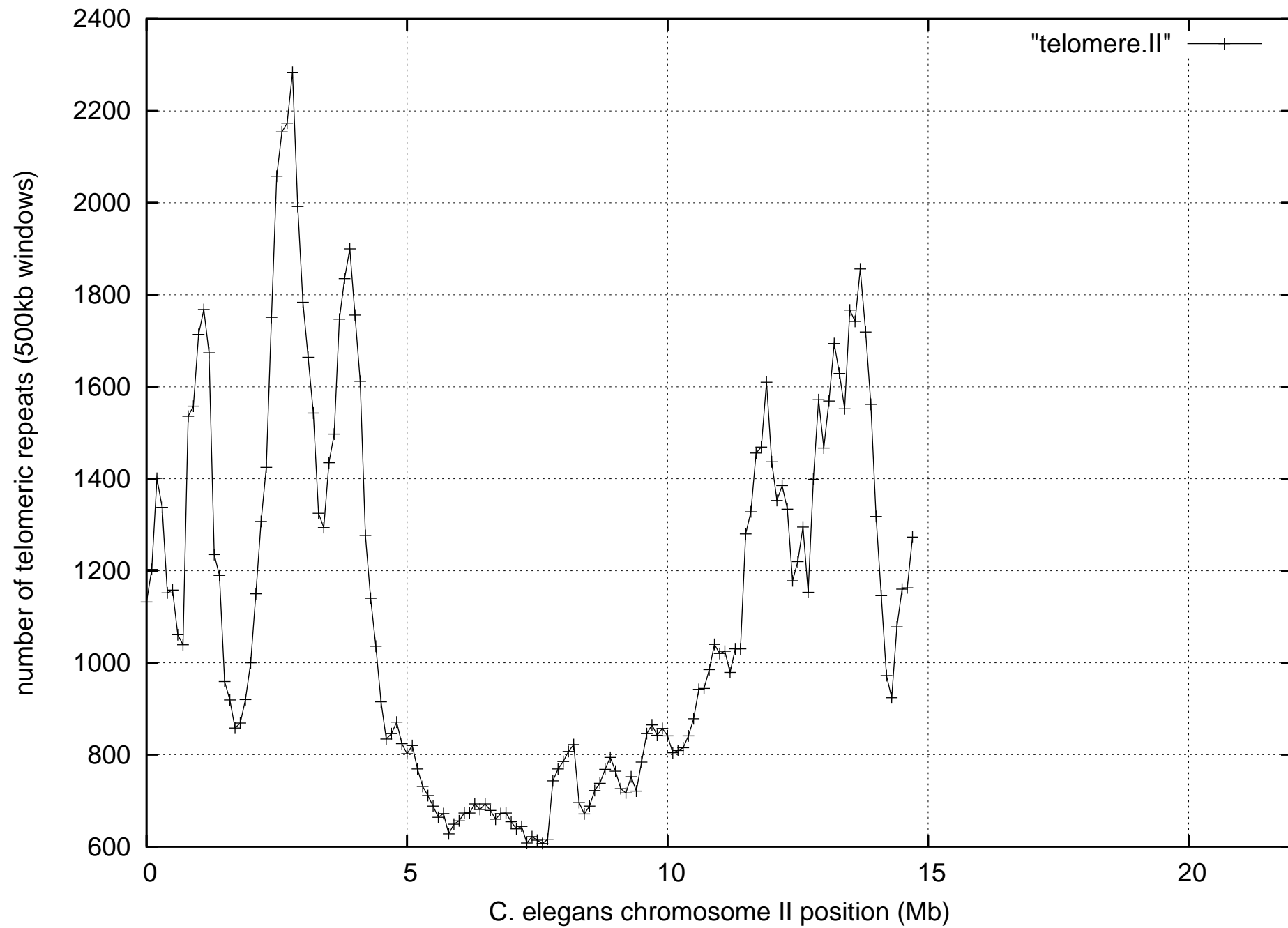

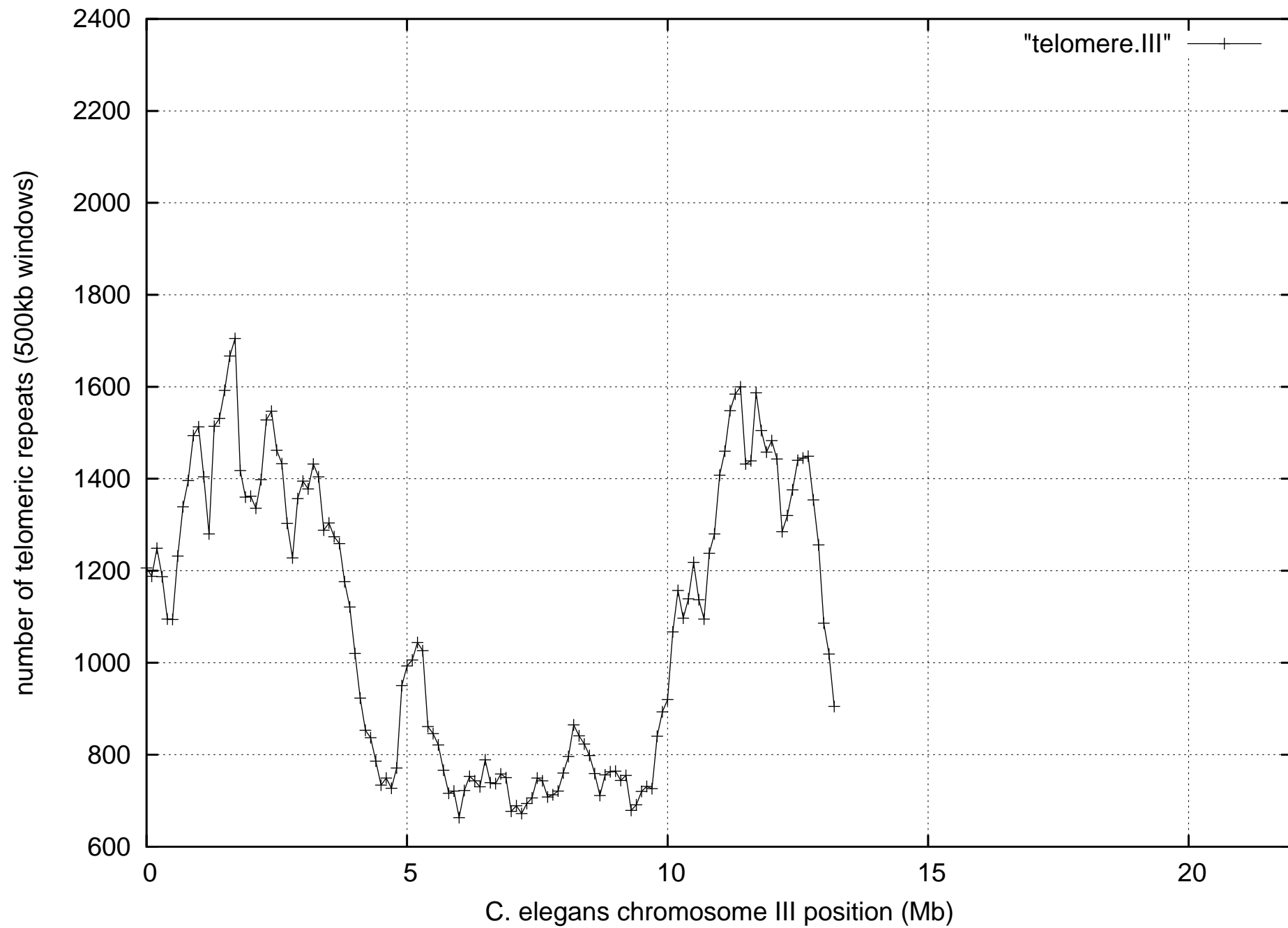

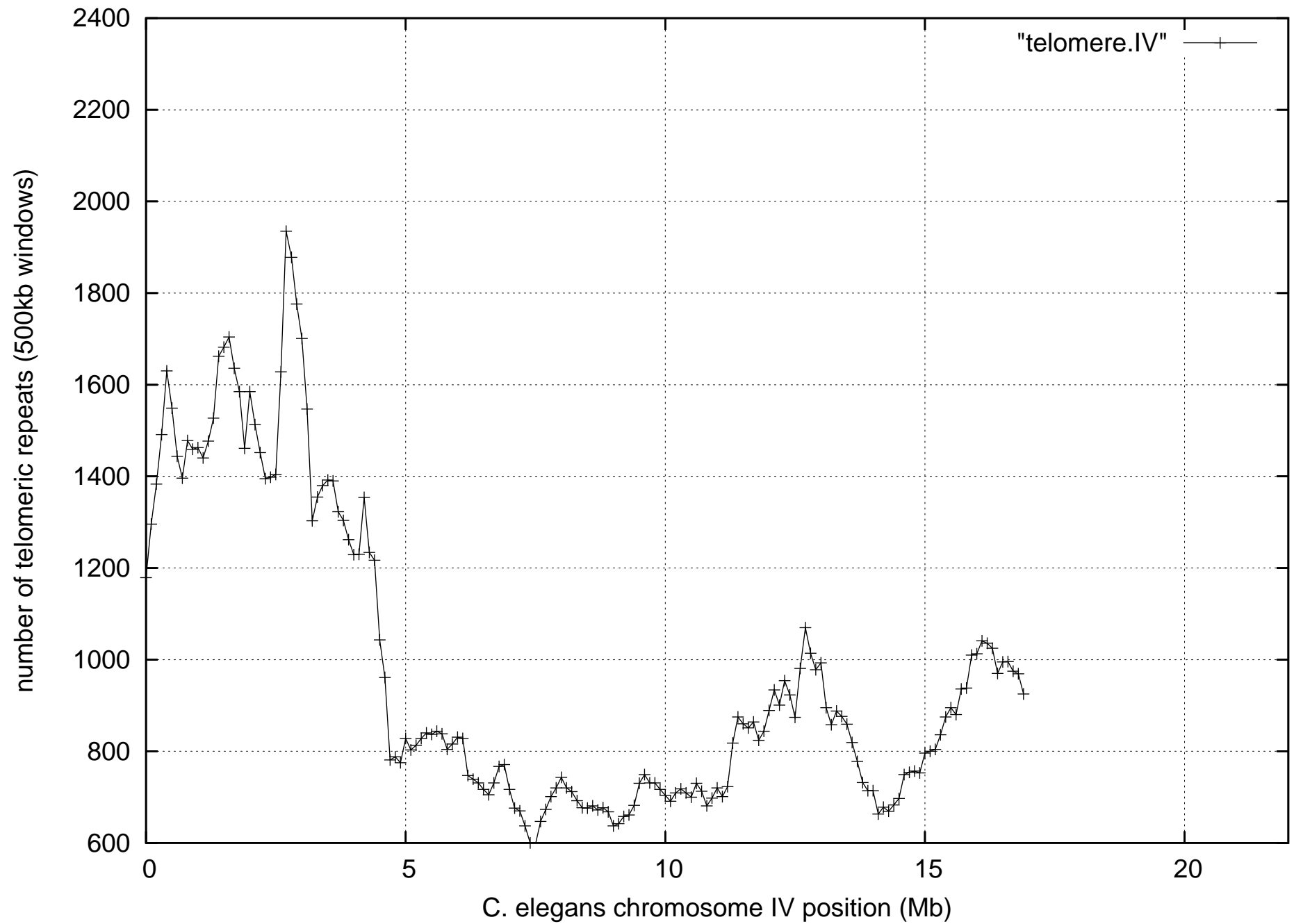

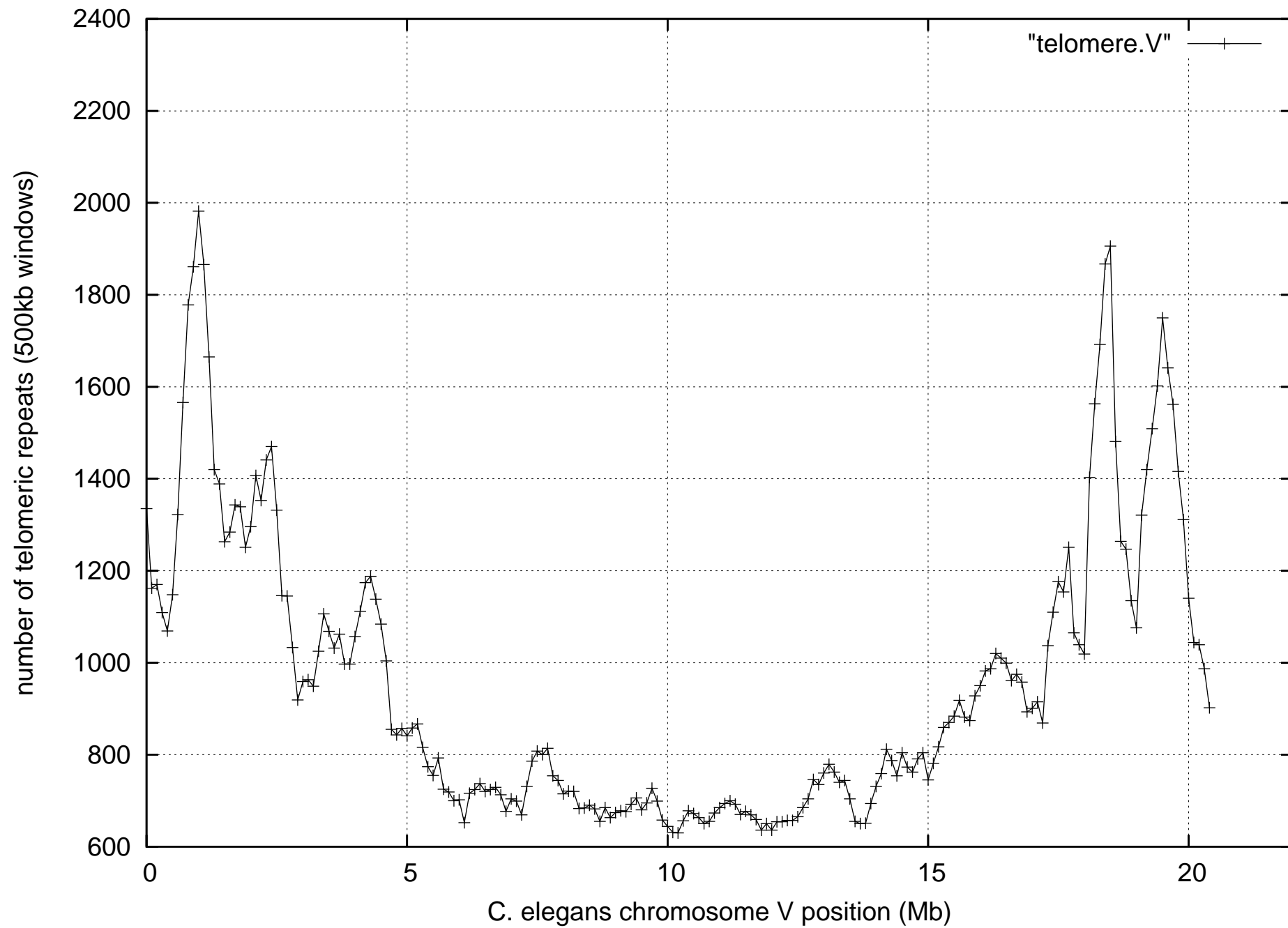

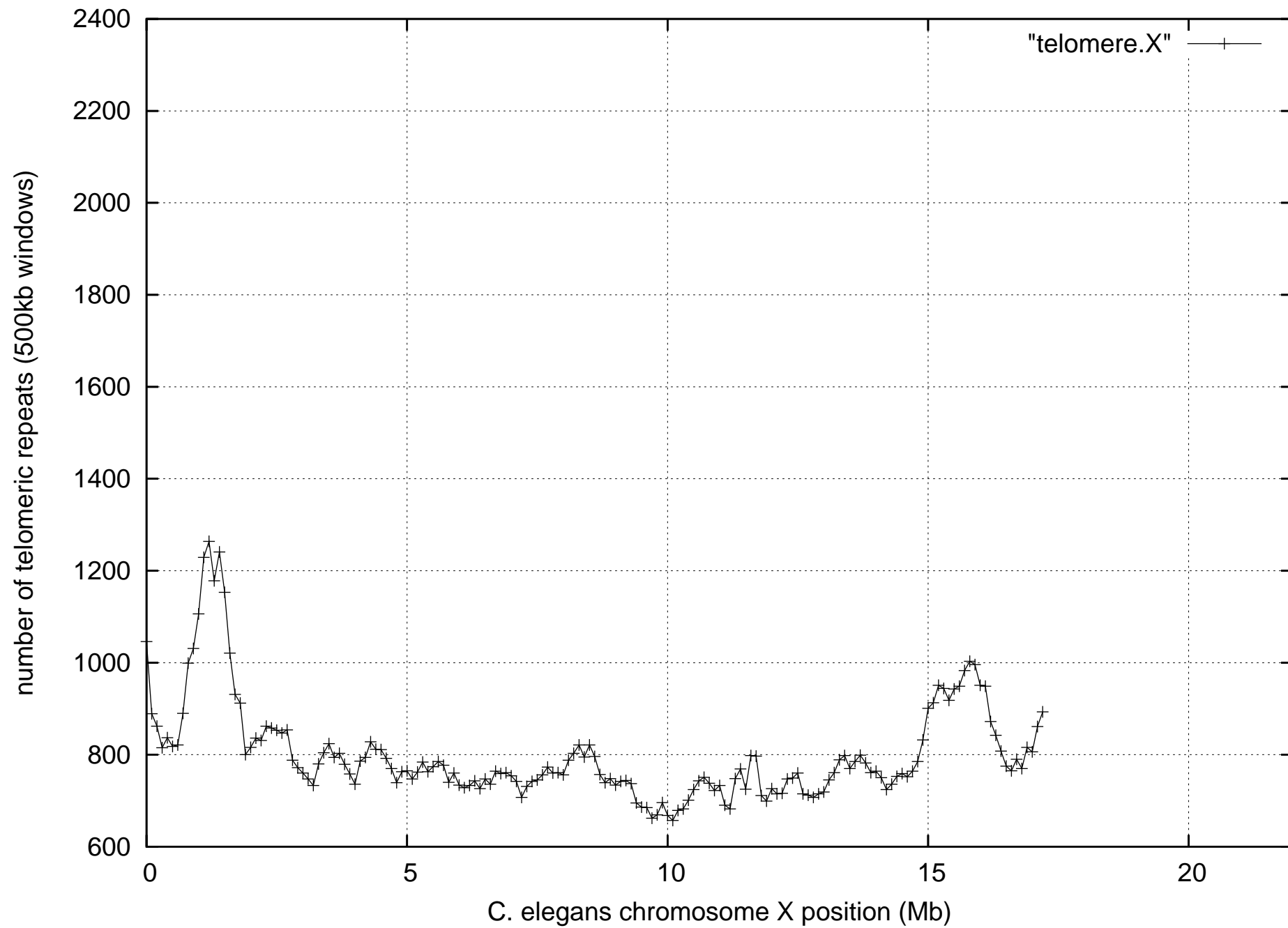

Supplement: Figure S2 — Graphs of each feature for each chromosome corresponding to the data provided in Dataset S4. (151 KB PDF) [file pbio.0050167.sg002.pdf]

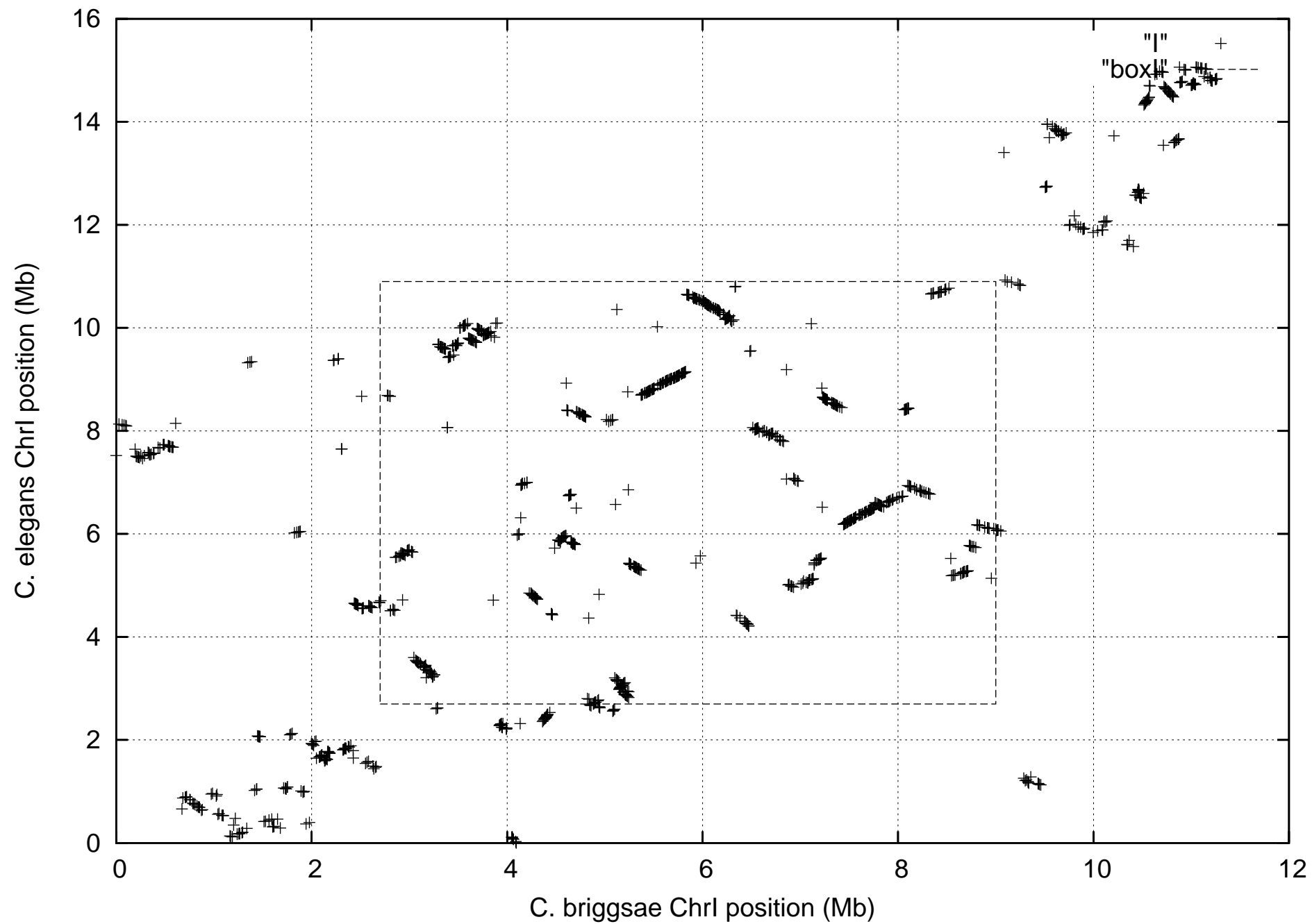

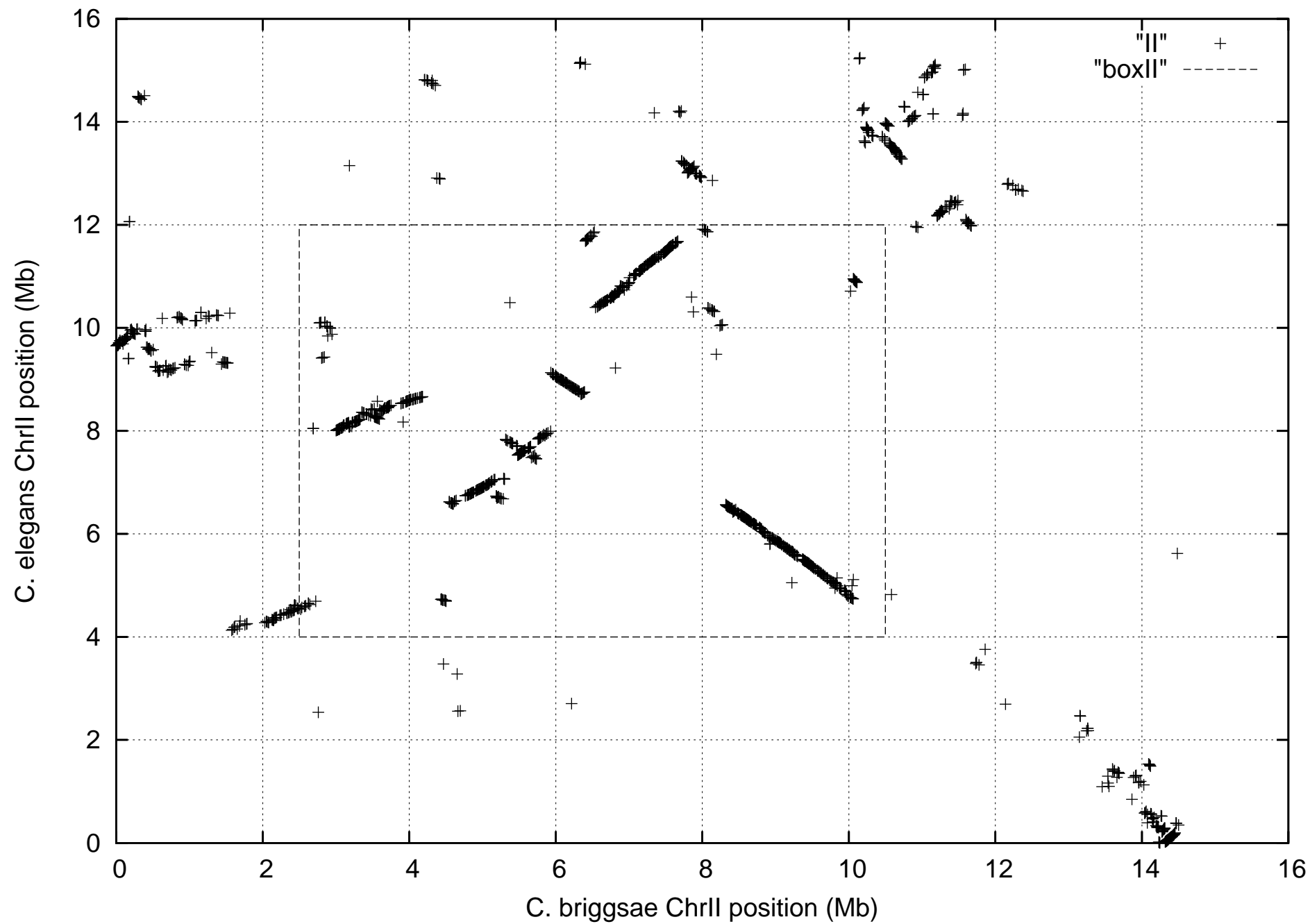

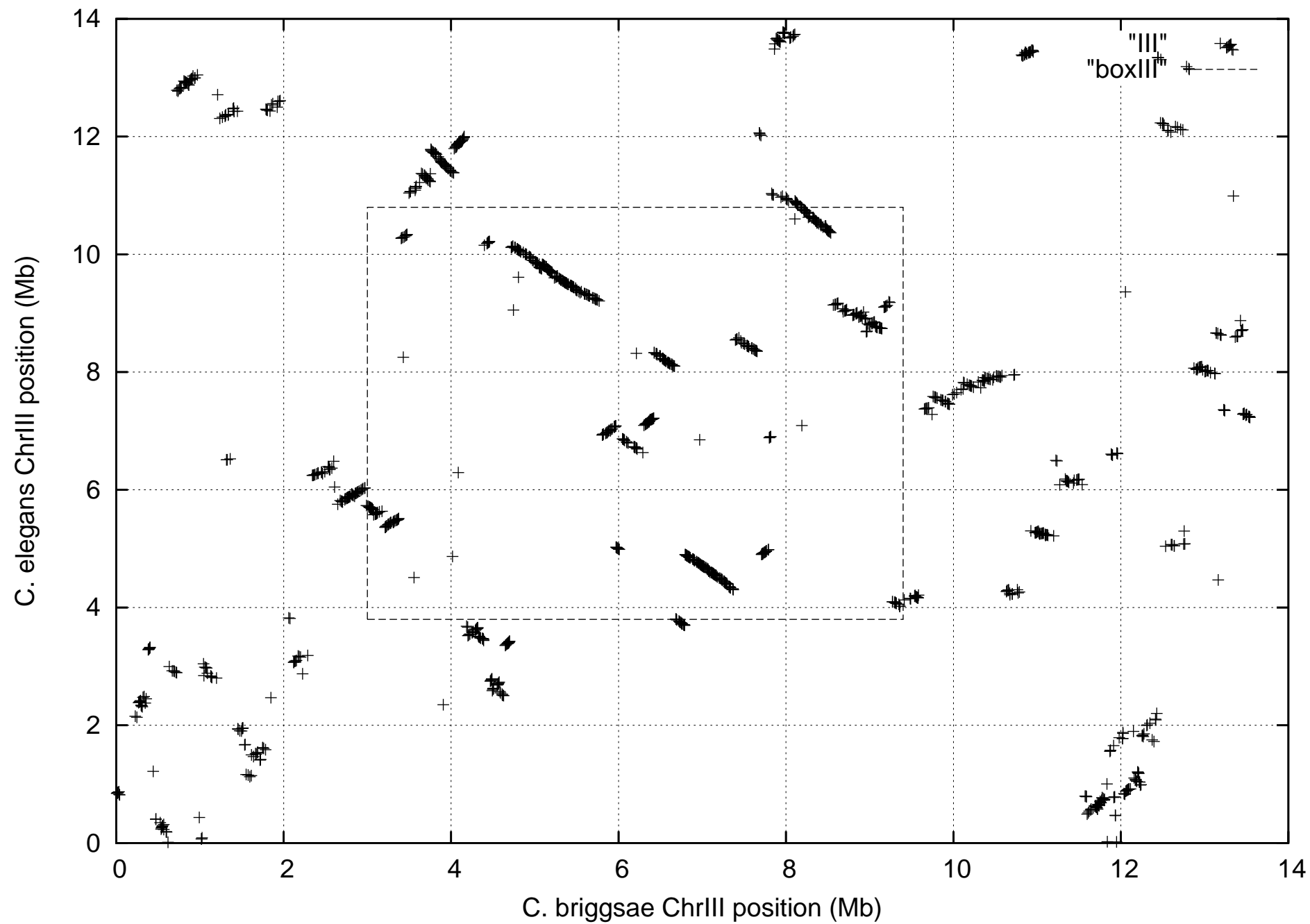

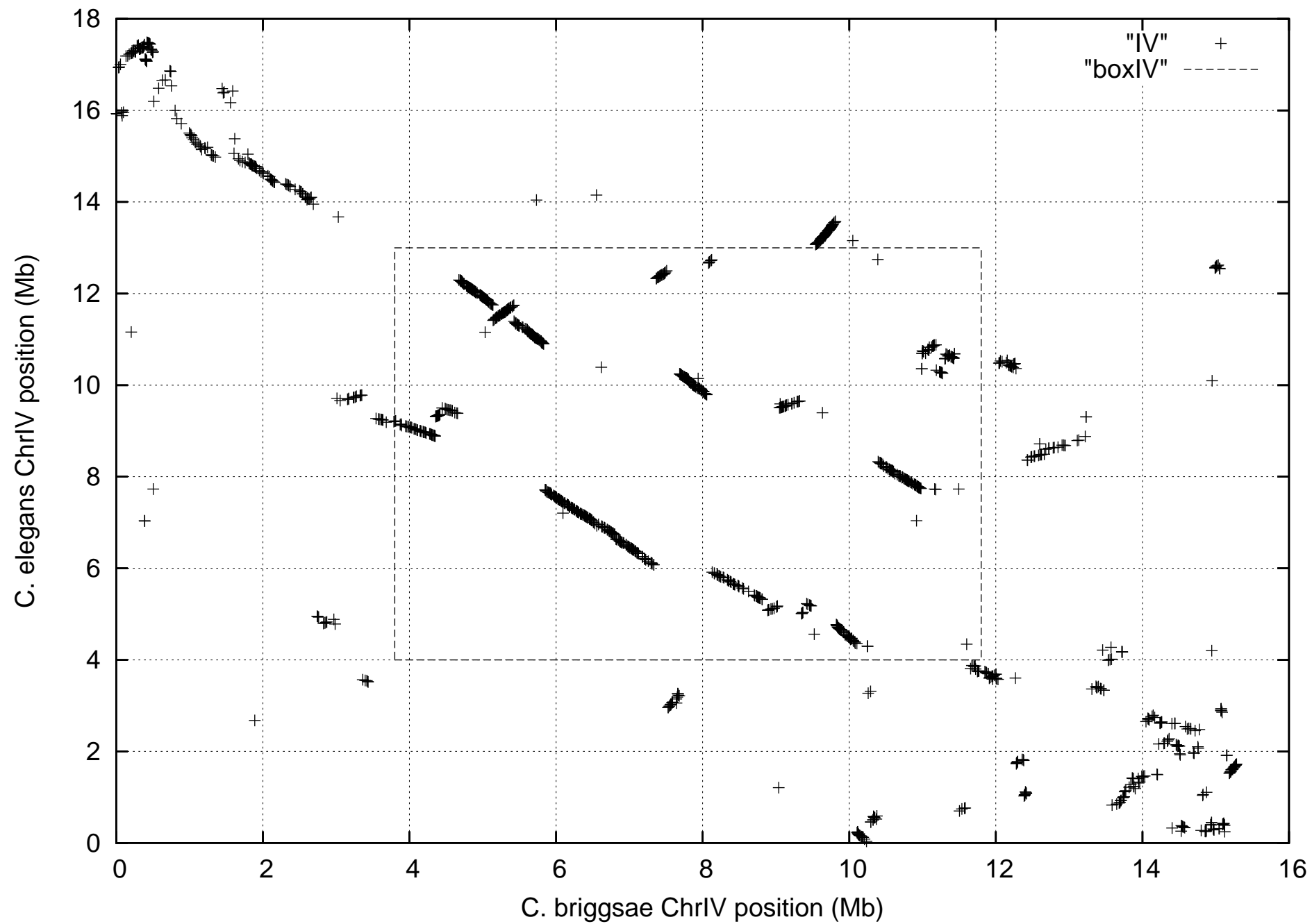

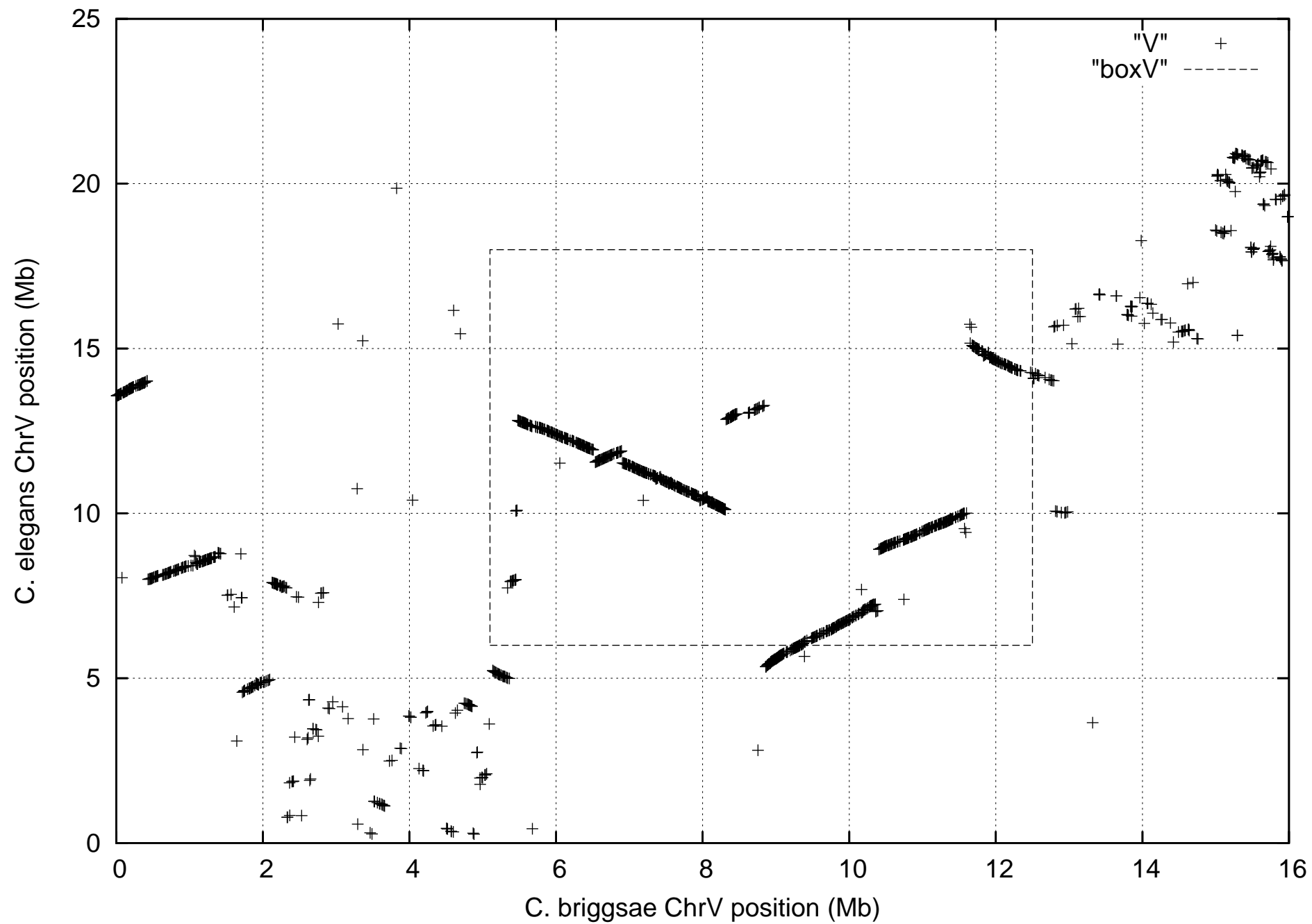

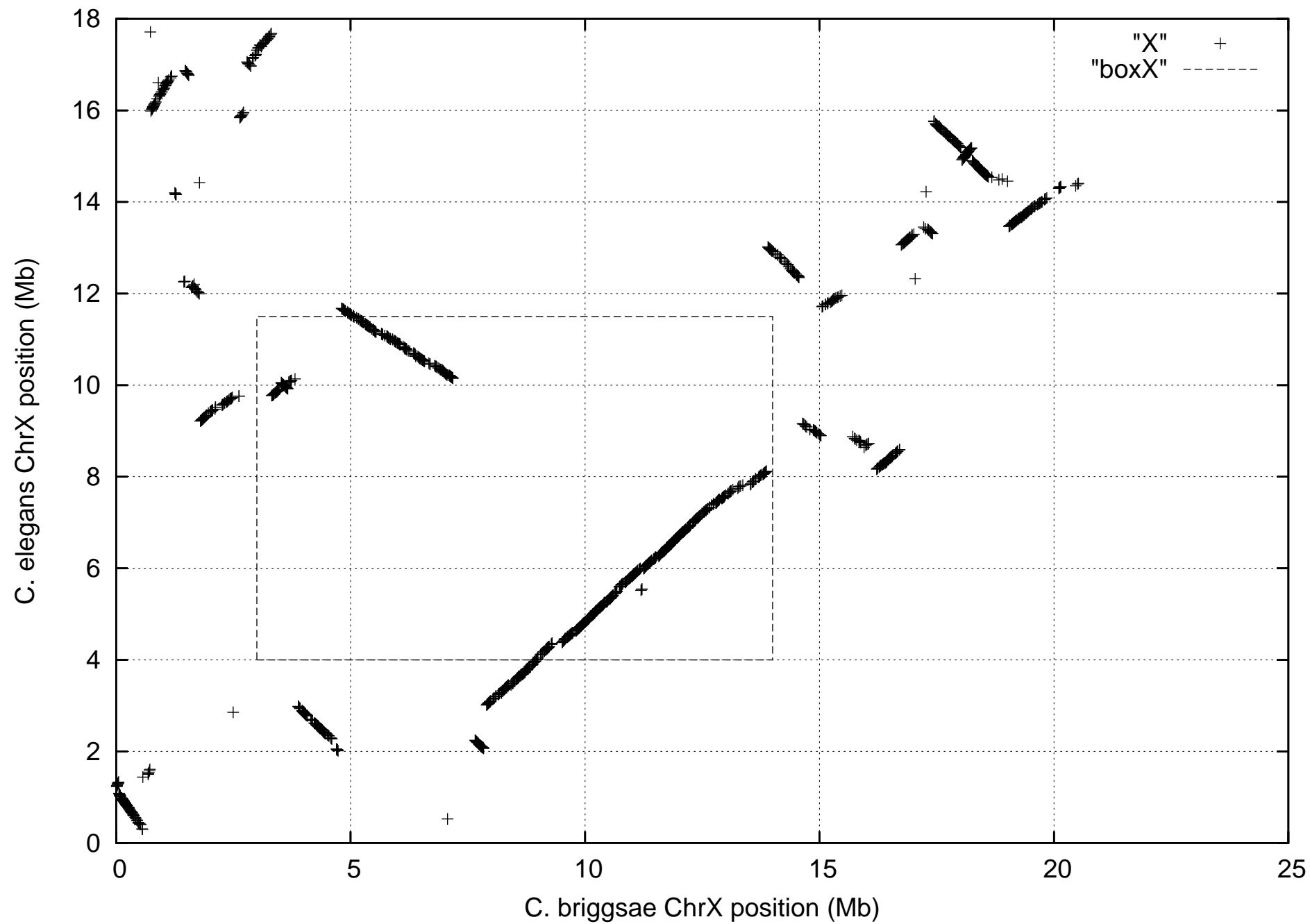

Supplement: Figure S3 — Graphical representations of the positions of 1:1 orthologs in both C. elegans and C. briggsae. Graphical representations are provided for all on-chromosome relationships. (150 KB PDF) [file pbio.0050167.sg003.pdf]
