# Supplementary material for: Comparison of C. elegans and C. briggsae Genome Sequences Reveals Extensive Conservation of Chromosome Organization and Synteny
Source: PLoS Biol. 2007 Jul 3;5(7):e167. doi: 10.1371/journal.pbio.0050167 (PMC1914384; doi:10.1371/journal.pbio.0050167)
Supplement: Figure S4 — Plot of the physical versus genetic map positions for C. elegans. (243 KB PDF) [file pbio.0050167.sg004.pdf]

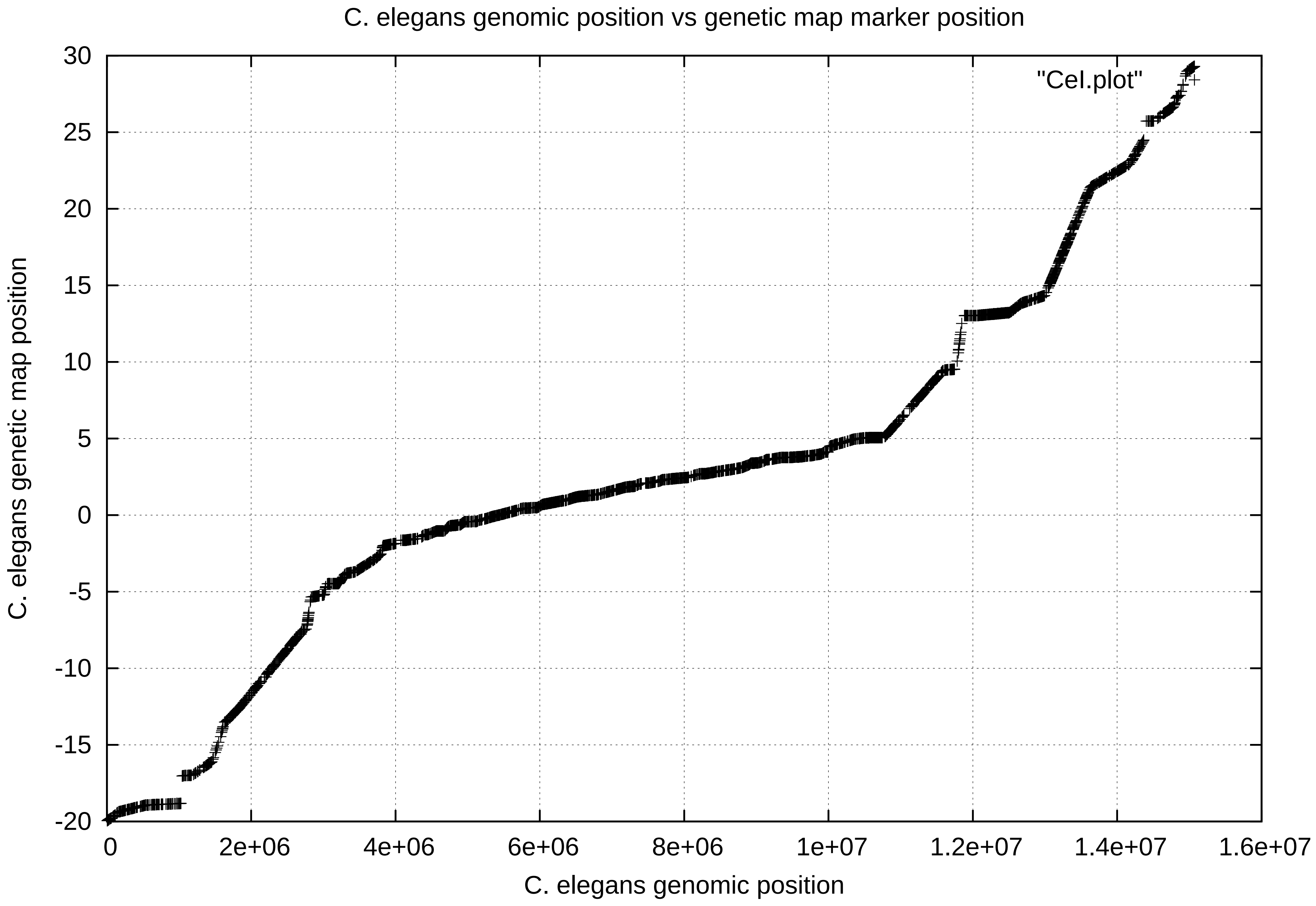

C. elegans genomic position vs genetic map marker position
"CeI.plot"
C. elegans genetic map position
C. elegans genomic position
30
25
20
15
10
5
0
-5
-10
-15
-20
0
2e+06
4e+06
6e+06
8e+06
1e+07
1.2e+07
1.4e+07
1.6e+07

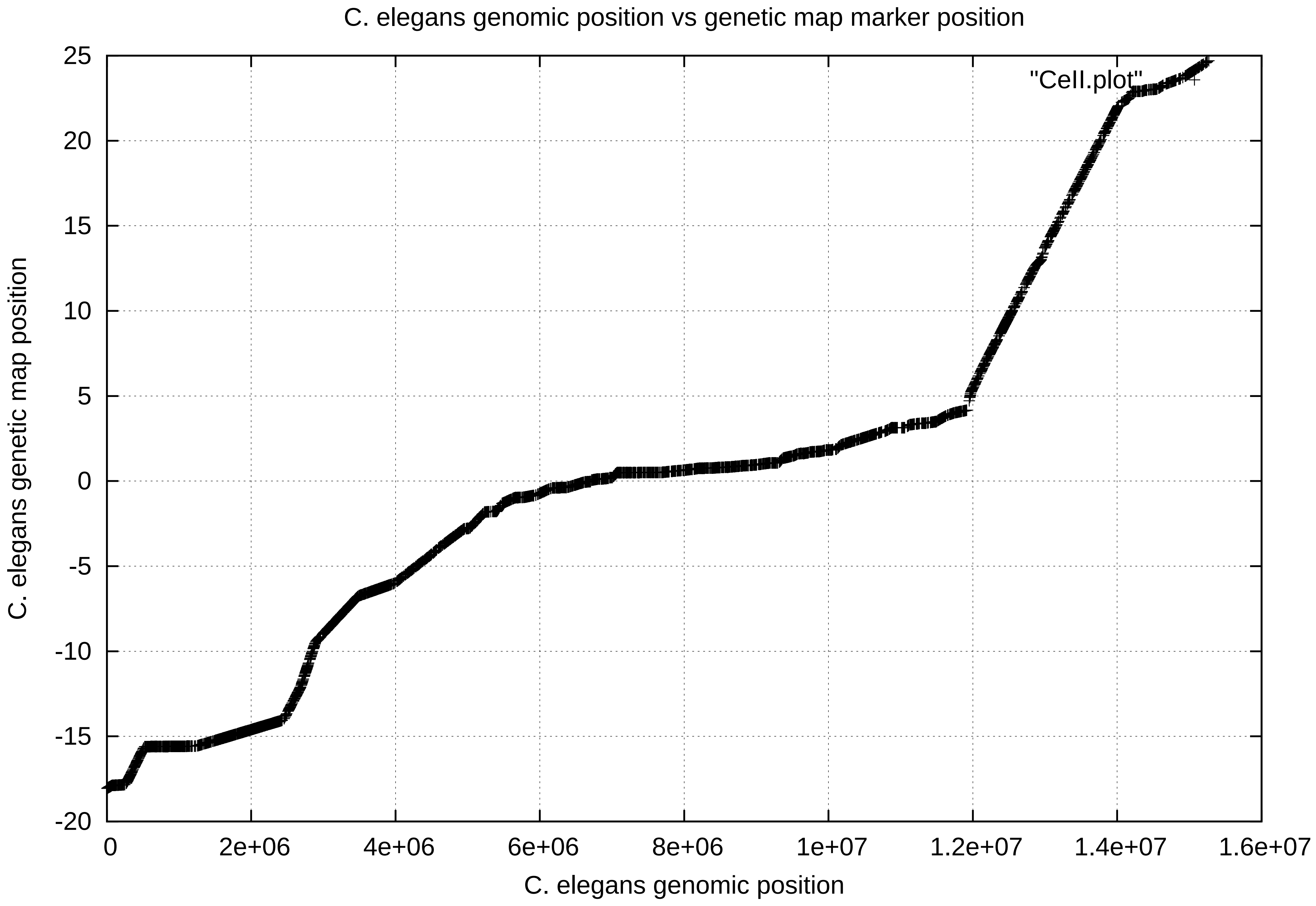

C. elegans genomic position vs genetic map marker position
"CeII.plot"
C. elegans genetic map position
C. elegans genomic position
25
20
15
10
5
0
-5
-10
-15
-20
0
2e+06
4e+06
6e+06
8e+06
1e+07
1.2e+07
1.4e+07
1.6e+07

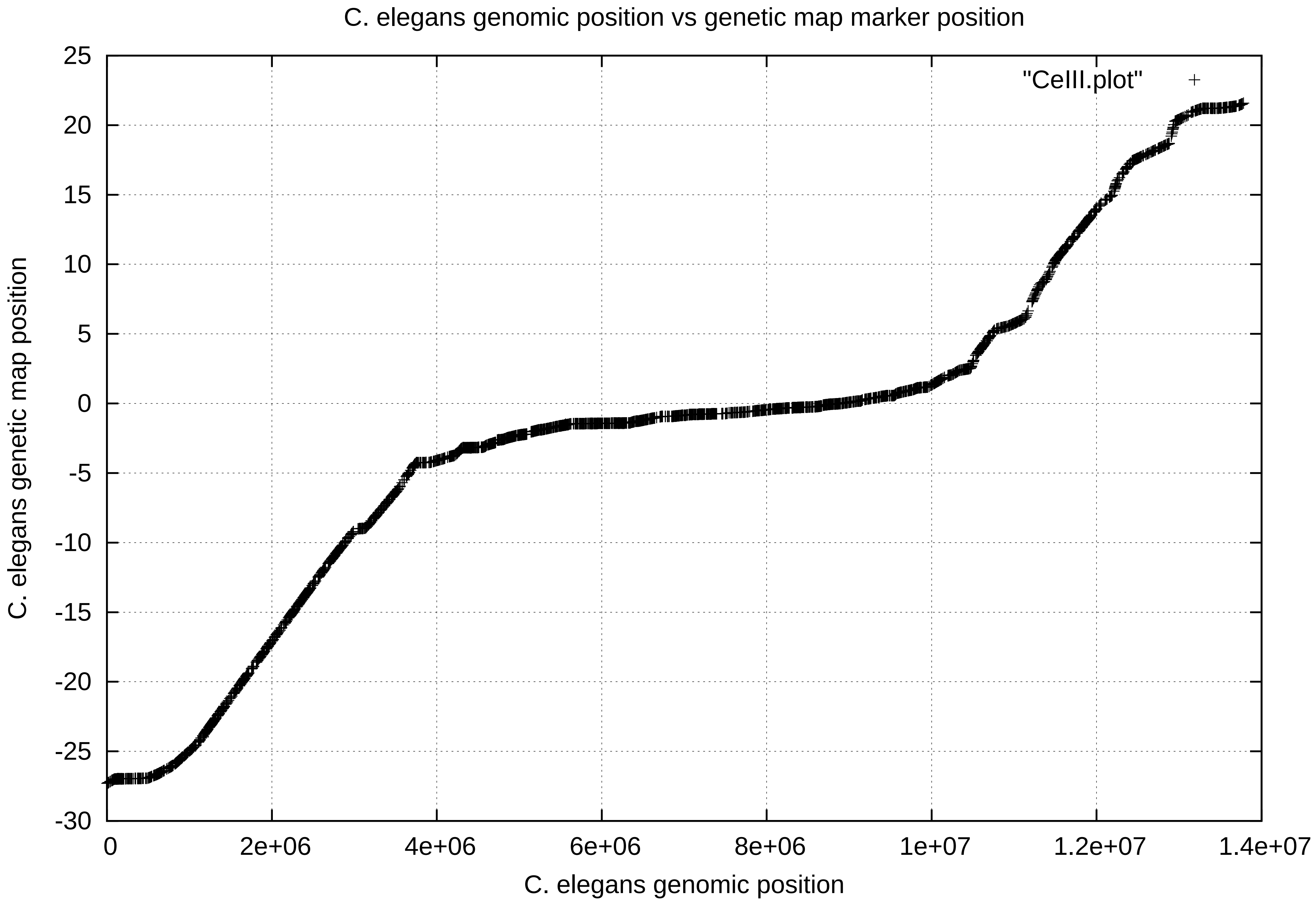

C. elegans genomic position vs genetic map marker position
"CeIII.plot"
C. elegans genetic map position
25
20
15
10
5
0
-5
-10
-15
-20
-25
-30
0
2e+06
4e+06
6e+06
8e+06
1e+07
1.2e+07
1.4e+07
C. elegans genomic position

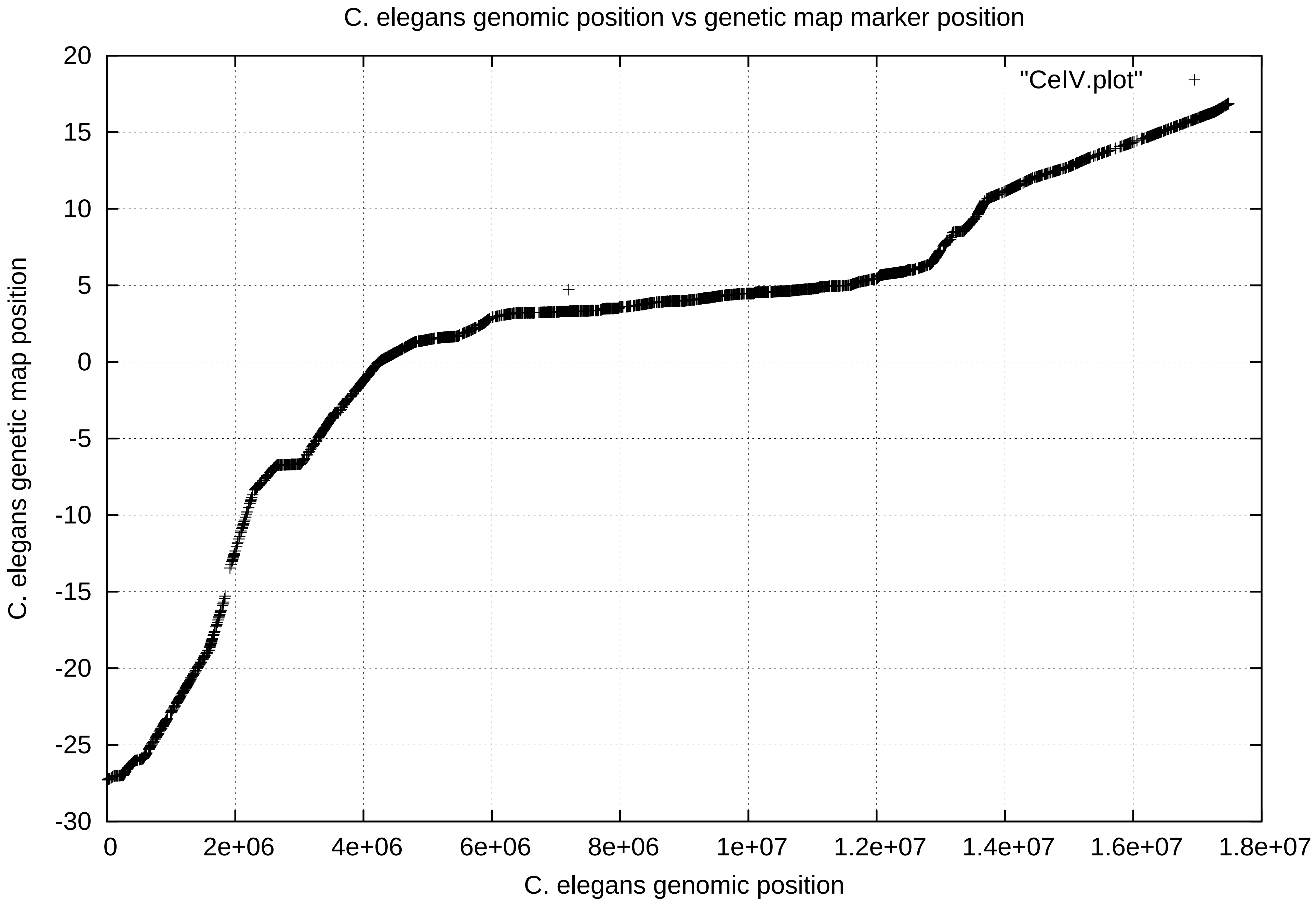

C. elegans genomic position vs genetic map marker position
"CeIV.plot"
C. elegans genetic map position
C. elegans genomic position
20
15
10
5
0
-5
-10
-15
-20
-25
-30
0
2e+06
4e+06
6e+06
8e+06
1e+07
1.2e+07
1.4e+07
1.6e+07
1.8e+07

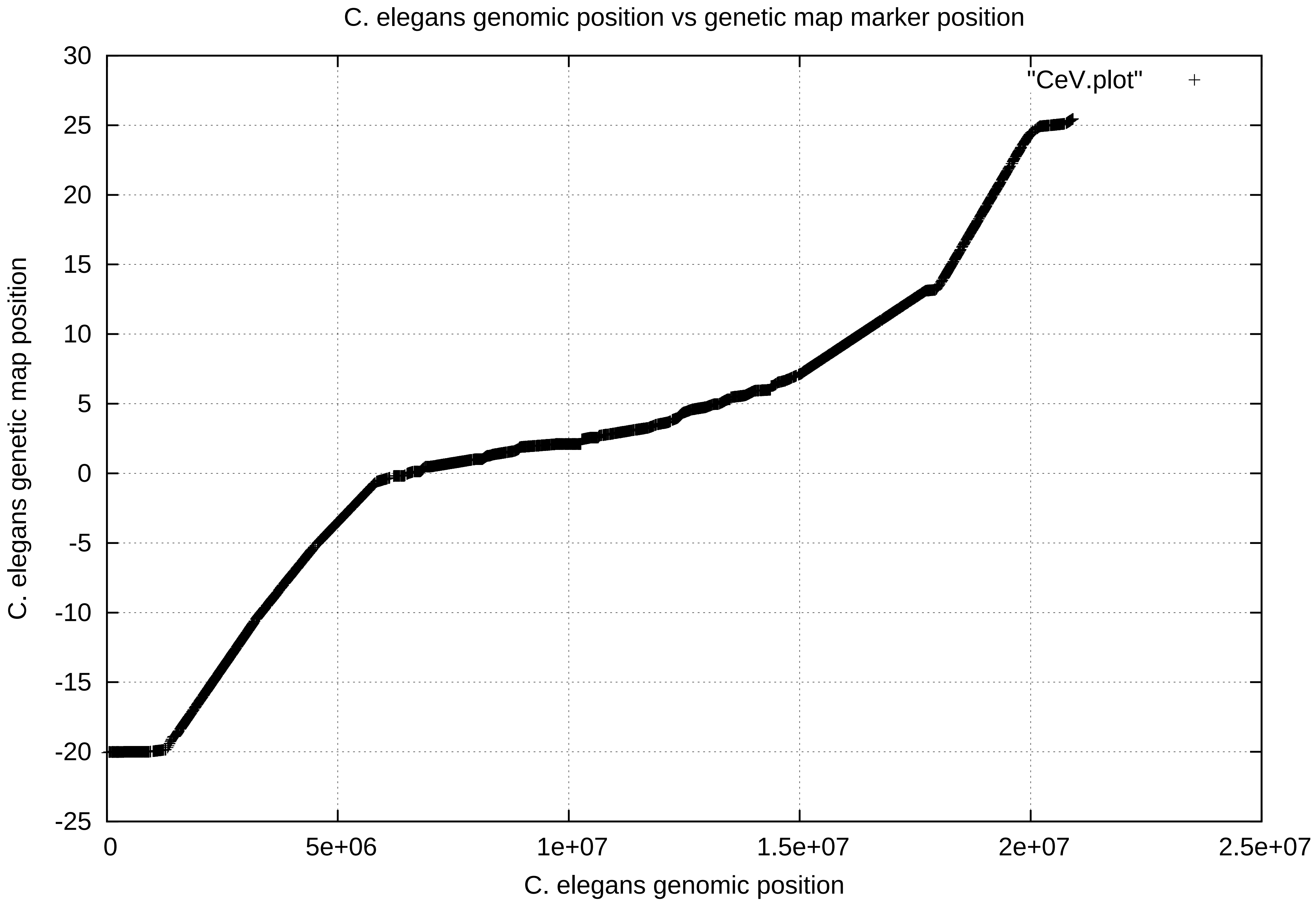

C. elegans genomic position vs genetic map marker position
"CeV.plot"
C. elegans genetic map position
C. elegans genomic position
30
25
20
15
10
5
0
-5
-10
-15
-20
-25
0
5e+06
1e+07
1.5e+07
2e+07
2.5e+07

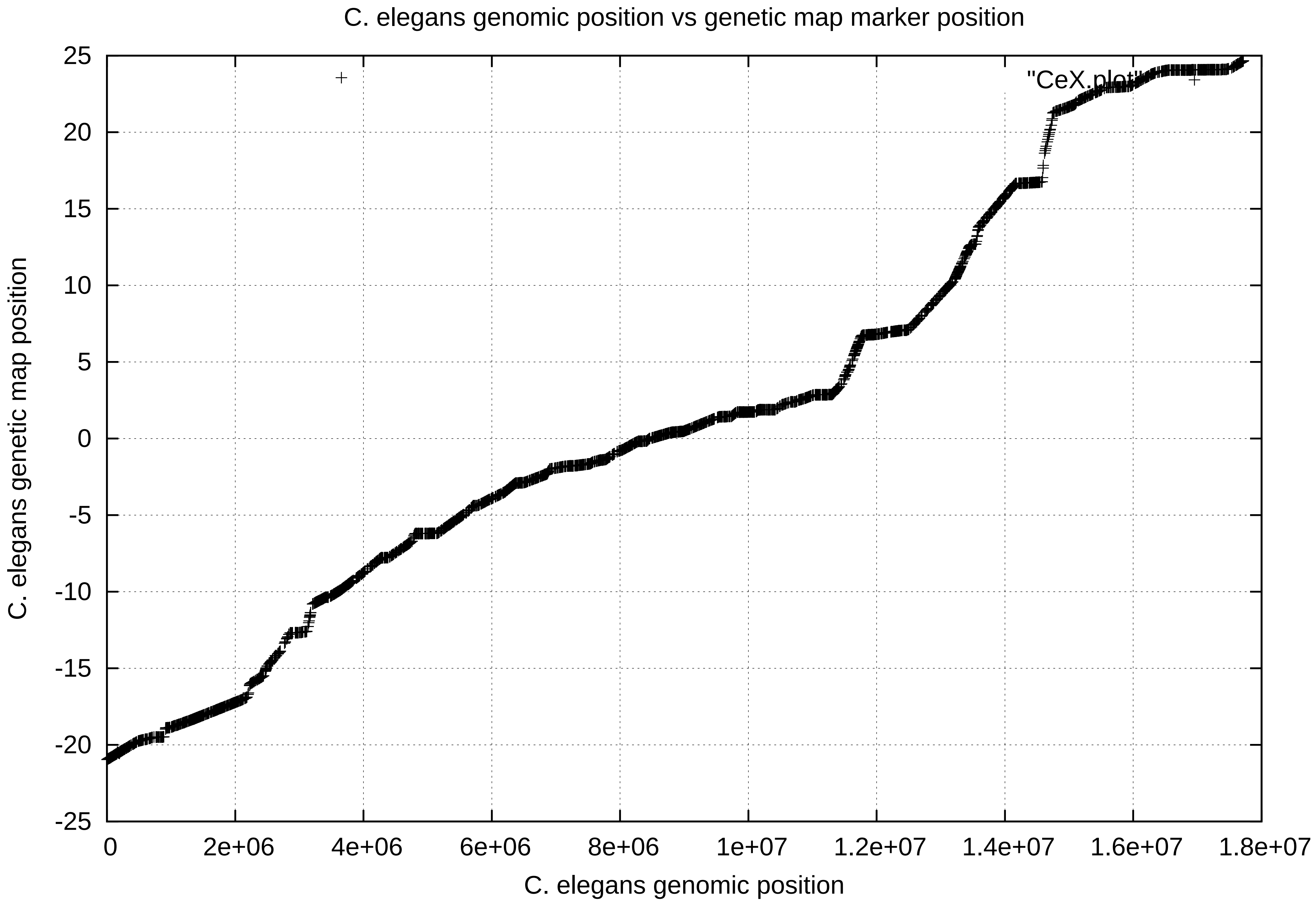

C. elegans genomic position vs genetic map marker position
"CeX.plot"
25
20
15
10
5
0
-5
-10
-15
-20
-25
C. elegans genetic map position
0
2e+06
4e+06
6e+06
8e+06
1e+07
1.2e+07
1.4e+07
1.6e+07
1.8e+07
C. elegans genomic position
